# Genome-wide analyses of LINE–LINE-mediated nonallelic homologous recombination

**DOI:** 10.1093/nar/gku1394

**Published:** 2015-01-22

**Authors:** Michał Startek, Przemyslaw Szafranski, Tomasz Gambin, Ian M. Campbell, Patricia Hixson, Chad A. Shaw, Paweł Stankiewicz, Anna Gambin

**Affiliations:** 1 Faculty of Mathematics, Informatics, and Mechanics, University of Warsaw, 2 Banacha street, 02-097 Warsaw, Poland; 2 Department of Molecular and Human Genetics, Baylor College of Medicine, One Baylor Plaza, Houston, TX 77030, USA; 3 Mossakowski Medical Research Centre, Polish Academy of Sciences, 5 Pawińskiego street, 02-106 Warsaw, Poland

## Abstract

Nonallelic homologous recombination (NAHR), occurring between low-copy repeats (LCRs) >10 kb in size and sharing >97% DNA sequence identity, is responsible for the majority of recurrent genomic rearrangements in the human genome. Recent studies have shown that transposable elements (TEs) can also mediate recurrent deletions and translocations, indicating the features of substrates that mediate NAHR may be significantly less stringent than previously believed. Using >4 kb length and >95% sequence identity criteria, we analyzed of the genome-wide distribution of long interspersed element (LINE) retrotransposon and their potential to mediate NAHR. We identified 17 005 directly oriented LINE pairs located <10 Mbp from each other as potential NAHR substrates, placing 82.8% of the human genome at risk of LINE–LINE-mediated instability. Cross-referencing these regions with CNVs in the Baylor College of Medicine clinical chromosomal microarray database of 36 285 patients, we identified 516 CNVs potentially mediated by LINEs. Using long-range PCR of five different genomic regions in a total of 44 patients, we confirmed that the CNV breakpoints in each patient map within the LINE elements. To additionally assess the scale of LINE–LINE/NAHR phenomenon in the human genome, we tested DNA samples from six healthy individuals on a custom aCGH microarray targeting LINE elements predicted to mediate CNVs and identified 25 LINE–LINE rearrangements. Our data indicate that LINE–LINE-mediated NAHR is widespread and under-recognized, and is an important mechanism of structural rearrangement contributing to human genomic variability.

## INTRODUCTION

Copy-number variation (CNV) contributes significantly both to human genetic variation as well as disease (1–3). Nonallelic homologous recombination (NAHR), occurring during meiosis, is the most common mechanism underlying the formation of recurrent CNVs in humans (4,5). The products of NAHR can be genomic deletions or reciprocal duplications, as well as inversions and inter- or intrachromosomal translocations (6,7). In the vast majority of rearrangements characterized thus far, NAHR occurred between large segments of the human genome that are present in more than one copy known as low-copy repeats (LCRs or segmental duplications). These LCRs are typically >10 kb in size and share >97% DNA sequence identity (4,6–8). However, in addition to these classically defined LCRs, other sequences have been observed to mediate apparent NAHR events. Specifically, rearrangements mediated by human endogenous retroviruses (HERVs) (9), a small subfamily of long retrotransposons comprising ∼0.8% of the human genome (9), suggest that the lower boundary on the length of the homologous region which is capable of mediating NAHRs might be as low as few kb. Likewise, other mobile DNA elements (10) may be potential substrates for NAHR. If true, this would indicate that a significantly higher fraction of the human genome is susceptible to NAHR-mediated rearrangements, as transposons make up as much as 44% of the reference human genome (11).

Since their initial discovery, studies have indicated that the presence of transposons, and particularly active transposons, has mutagenic effects on the genome of their host (12). The most frequently cited effects of transposons are the capability of disrupting a gene by insertion, disruption of promoter regions and the ability to misregulate gene expression by transposon-borne enhancers (13). This mutational activity of transposons is significant enough that it has been selected against over the course of evolution (14). Computational modeling approaches suggested that, in certain circumstances, the activity of transposons may be beneficial to the population by assisting the adaptation of the population to a new environment (15).

Another distinct class of the mutagenic effects of transposons, perhaps comparable in scale to that of *de novo* insertions (16) is that caused by their high self-similarity coupled with abundance in the genome. These features create a large number of non-allelic, homologous sites in the genome that can mediate recombination events (17,18). Cases of LINE–LINE/NAHR have been reported previously (19), and indeed some of them linked to disease (20–23). Moreover, previous studies of genomic architectural features that stimulate and potentially catalyze pathogenic microdeletions and tandem duplications have found that repetitive elements are enriched at deletion breakpoints (24). Interestingly, comparative analysis of human and chimpanzee genomes, verified by wet-lab analyses (25), identified 73 human-specific LINE recombination-associated deletion (55 of them classified as NAHR events).

Here, we analyzed the contribution of LINE retrotransposons, a much more abundant family of retrotransposons than HERVs, to NAHR in humans. We also performed an analysis of human genome susceptibility to LINE-mediated NAHR deletions, duplications, inversions and translocations using the Baylor College of Medicine (BCM) Medical Genetics Laboratory (MGL) clinical database of CNVs. We found that LINE–LINE-mediated NAHR occurs more frequently than previously thought and indeed on a genome-wide scale. Finally, we provide several novel bioinformatic procedures and algorithms for the study of NAHR.

## MATERIALS AND METHODS

### Identification of LINE pairs potentially mediating NAHR

We downloaded the reference DNA sequence from the hg19 assembly of the human genome along with coordinates of all LINE elements as denoted by the UCSC *RepeatMasker* track (26). Locations of centromeres were obtained from the *Gap* UCSC (27,28) genome browser track. The sequences of 124 150 LINE elements longer than 1 kb were extracted, along with a 3-kb flanking sequence. These sequences were then pairwise-aligned using the BLAST algorithm (29) with the low-complexity sequence masking disabled. We found 3 642 718 496 statistically significant High-scoring segment pairs (HSPs), with the *E*-value computed by BLAST being <10^−50^, which were further filtered to eliminate self-alignments. Moreover, we removed cases, in which the alignment extended outside of the LINE into the flanking regions, suggesting that the duplicated sequence may not be the result of LINE–LINE recombination, but rather that the LINE element was a part of a larger LCR. We also excluded alignments shorter than 1000 bp, those with the identity <92%, and pairs that would result in intrachromosomal CNVs >10 Mbp (as such CNVs are unlikely to be observed in a living organism). Alignments were classified into types (deletion, duplication, inversion or translocation) based on whether the matching LINE pair maps to the same chromosome, on each element's respective orientation, and on whether the potential NAHR event spans a centromere. Pairs of LINEs on different chromosomes, were marked as potential translocation substrates. The remainder (pairs mapping on the same chromosome) were marked as either deletion/duplication substrates (if directly oriented and mapping on the same side of the centromere) or inversion substrates (if not directly oriented, regardless of their position with respect to the centromere).

**Figure 1.**
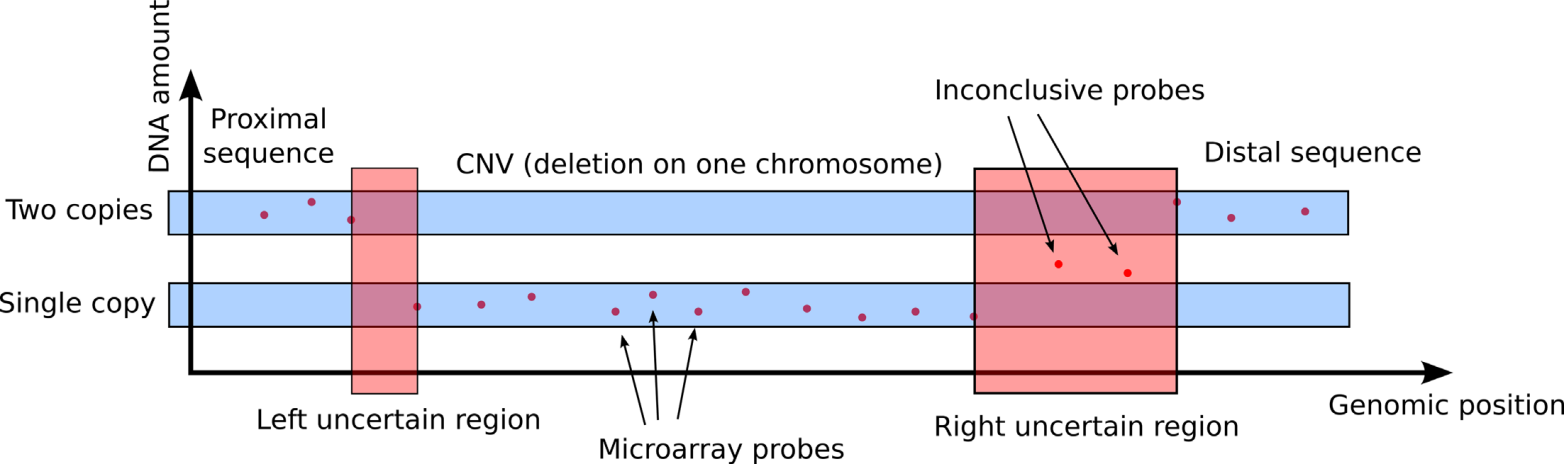
Schematic representation used to define the *breakpoint uncertainty regions* for analyses. The proximal breakpoint is predicted to map within the left red area, the distal one within the right red area. A similar approach was used for duplications.

We subsequently intersected the directly-oriented LINE–LINE pairs with our clinical database of CNVs. This database consists of 398 468 CNVs that were identified in 36 285 patients who underwent oligonucleotide chromosomal microarray analysis (CMA) at the Medical Genetics Laboratories at BCM. Moreover, some of the CNVs were determined to be pathogenic or potentially pathogenic by the clinical cytogeneticist reviewing the case. All DNA samples were anonymized for further study, and no clinical information is unavailable. The precise breakpoint of each CNV was unknown; we narrowed their putative locations to the regions between two adjacent oligo probes showing definitive difference in log_2_ ratio. We refer to these intervals as breakpoint uncertainty regions (cf. Figure 1).

The set of CNVs was then filtered to exclude the cases where the uncertainty regions at both ends of the CNV were located in or contained directly-oriented paralogous LCRs (DP-LCRs). We assumed that in those cases the NAHR might have been mediated by these DP-LCRs, rather than LINEs specifically. After DP-LCR filtering, 358 160 CNVs remained and were compared to the database of possible LINE–LINE/NAHR pairs computed previously. The analysis yielded 112 520 potential CNVs. The parameters were further constrained to include only full- and nearly full-length LINEs (>4 kb and aligning over >4 kb of their length) with >96% sequence identity, yielding 516 CVNs having log_2_ ratio <−0.6 or >0.5.

### Clinical CMA

DNA was prepared from peripheral blood using the Puregene DNA isolation kit (Gentra Systems, Minneapolis, MN, USA) according to the manufacturer's instructions. CMA was performed with gender-matched controls; labeling, hybridization and scanning procedures as well as computational analysis have been described previously described (30). Briefly, BCM MGL oligonucleotide arrays contain both genome-wide backbone probe coverage and enhanced probe resolution within the exons and introns of manually curated known and putative disease genes.

### Subjects

**Figure 2.**
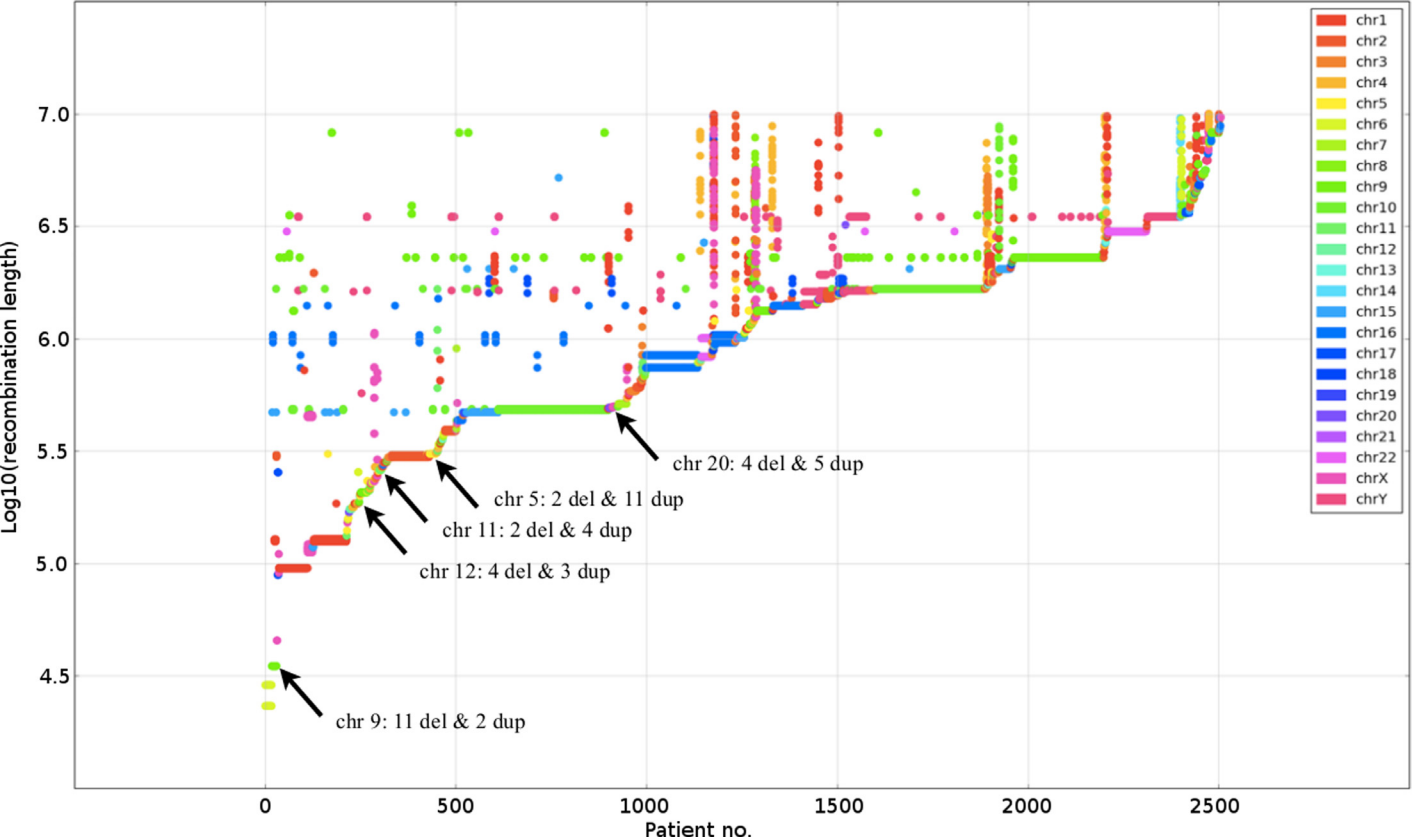
Scatterplot of patients with CNVs where a pair of LINEs predicted to mediate NAHR lies in both *breakpoint uncertainty regions*. Only cases where the alignment between LINEs is >4 kb with over 96% identity are shown. Patients are sorted by the length of their shortest CNV; one patient may possess multiple CNVs. Cases selected for PCR confirmation are highlighted with arrows.

Deidentified DNA samples from 44 individuals harboring potential LINE–LINE/NAHR CNVs, 21 deletions and 23 duplications, from five different genomic regions (Figure 2) were obtained from unrelated subjects identified by CMA (CMA oligonucleotide versions V7.1, V7.2, V7.4, V7.6, V8.1, V8.3, V9.1) (31,32). Additionally, DNA samples were obtained from six healthy individuals, following informed consent (BCM IRB protocol H33409).

### Long range PCR and DNA sequencing

**Figure 3.**
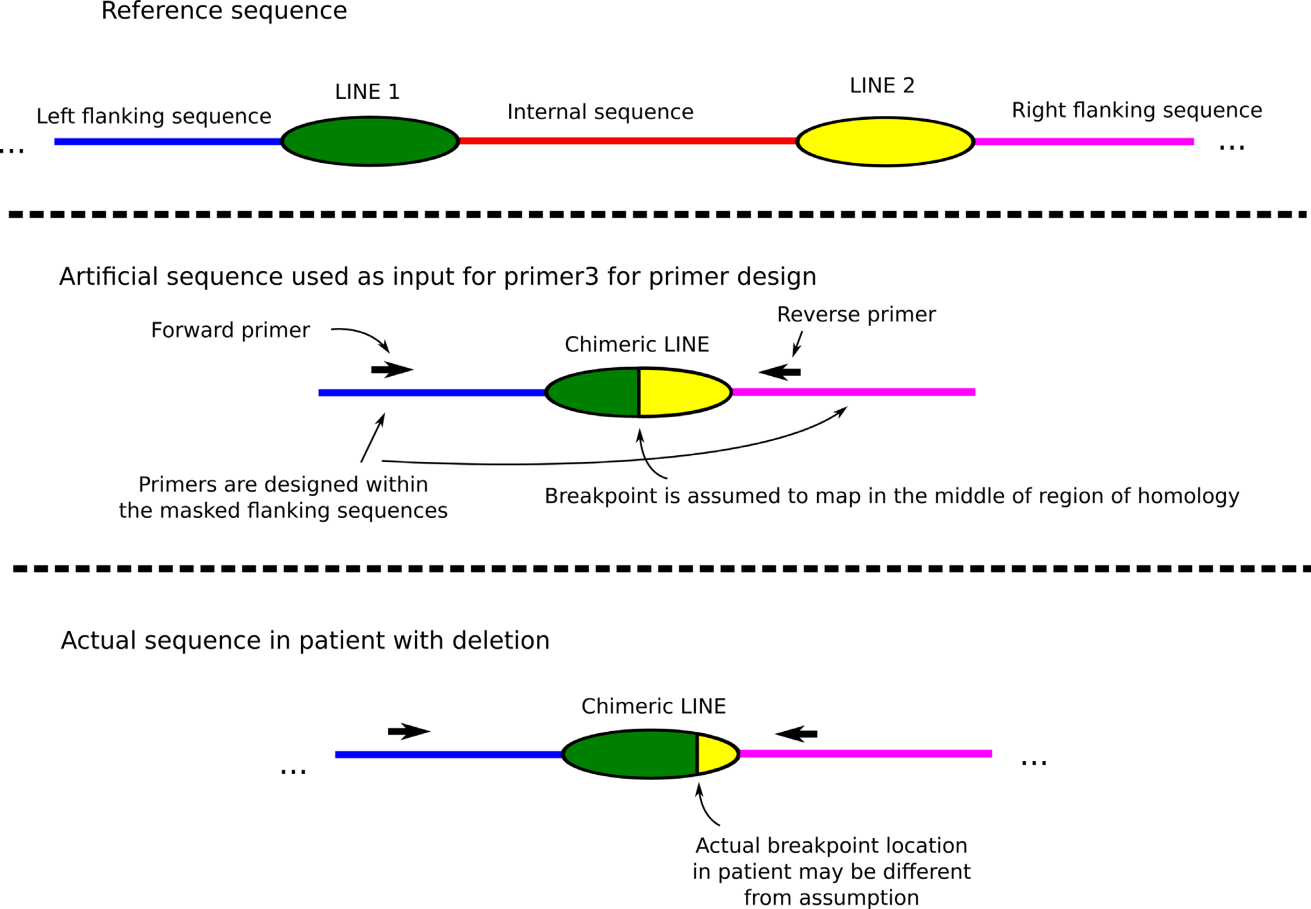
Artificial sequences computed for primer design for detection of chimeric LINE sequences. Shown on figure is the process for deletion, with duplications and inversions being handled in similar fashion.

Long-range PCR (LR-PCR) primers flanking LINE elements were automatically designed using custom software, including code from Primer3 (http://primer3.sourceforge.net/) (33). The program automatically generates a hybrid LINE sequence (assuming that the breakpoint maps within LINE–LINE homology region) along with unique flanking sequence for all possible NAHR rearrangements (deletion, duplication, inversion or translocation) (Figure 3). These hybrid sequences were then analyzed by a custom Primer3 script to obtain LR-PCR primers.

LR-PCR amplification of 7–15 kb fragments was performed using LA *Taq* Polymerase (TaKaRa Bio USA, Madison, WI, USA) following the manufacturer's protocol. Briefly, we used 25 μl reaction mixtures containing 100 ng genomic DNA, 0.4 mM dNTPs, 0.2 μM of each primer, and 1.25 U of LA Taq polymerase mix. PCR conditions were: 94°C for 1 min, followed by 30 cycles at 94°C for 30 s and 68°C for 12 min, and 72°C for 10 min. PCR products were treated with ExoSAP-IT (USB, Cleveland, OH, USA) to remove unconsumed dNTPs and inactivate primers. The treated amplicons were then sequenced by the Sanger method (Lone Star Labs, Houston, TX, USA) using the initial primers and primers specific for both unmasked proximal and distal copies of the LINEs.

### LR-PCR analyze of healthy subjects

From the set of computationally predicted LINE–LINE flanked loci, we also selected 95 directly-oriented LINE pairs with high homology parameters to study potential LINE–LINE-mediated deletions and duplications, and 95 inverted pairs to study inversions. We designed PCR primers as described above (one set of 95 primer pairs for deletions and a matching set for duplications, and one set for inversions). LR-PCR reactions (with the same parameters as described earlier) were run on a mixture of DNA samples (8 × 50 ng) from eight healthy subjects not known to suffer from genetic disease. Evidence for CNV was determined by visualizing a band following gel electrophoresis, and comparing the expected amplicon length (computed during the design of primers) to the observed length of the DNA amplicon.

### Validation of PCR approach

To rule out the possibility of presented results being an artifact of PCR reaction (caused by stalling, and the stalled products annealing to source DNA, resulting in a PCR-generated recombination product) we have repeated the PCR reaction on selected patients with different primer sets (see Supplementary Figure S1). The obtained amplicons have been Sanger sequenced and, in all cases, the junction sites were the same as in the original experiments (cf. Supplementary Figures S2 and S3).

### Array CGH analysis of healthy subjects

Genomic CNVs were analyzed using a custom-designed genome-wide LINE–LINE-targeted 4 × 180k comparative genomic hybridization (CGH) microarray (Agilent Technologies, Santa Clara, CA, USA). The array was designed using a set of custom scripts, written in the Python programming language. Probes were selected from the database of 26 million Agilent high-density oligonucleotide probes. In addition to backbone probes used for calibration, each LINE from the set of the directly oriented LINE–LINE/NAHR pairs was flanked with five oligonucleotide probes on each side to detect CNVs with both breakpoints mapping within LINE elements. For each array, one healthy individual's DNA was labeled with Cy3 and different, sex-matched healthy individual's DNA was labeled with Cy5. The labeling and hybridization procedures were performed according to manufacturer's protocols (Agilent Technologies, Santa Clara, CA, USA).

### DNA sequence analysis

Genomic sequences defined by coordinates identified in the array CGH experiments, were downloaded from the UCSC genome browser (NCBI build 37, May 2009, http://www.genome.ucsc.edu) and assembled using the Sequencher v4.8 software (Gene Codes, Ann Arbor, MI, USA). Interspersed repeat sequences were identified using RepeatMasker UCSC (http://www.repeatmasker.org) track.

### Search for motifs associated with genomic variability

We performed a *de novo* study of motifs associated with recombination with the MEME software suite (34,35), using the LINEs identified as NAHR-mediating in this study and from the literature. We also used other, randomly selected LINE elements as background. Additionally, we performed a search for known motifs associated with recombination in the NAHR-mediating LINEs using MAST software (36).

### Hidden Markov model for breakpoints identification

**Figure 4.**
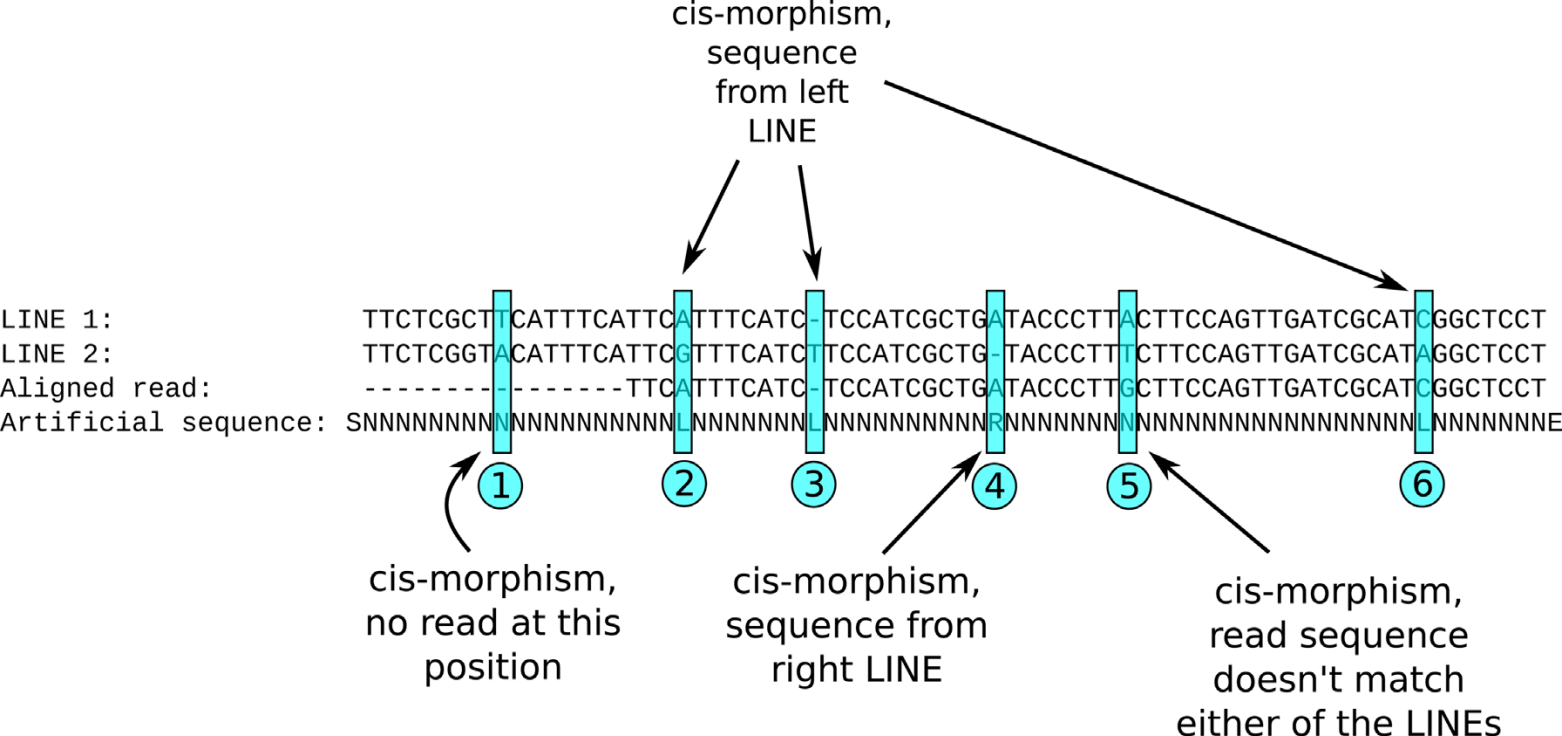
Construction of input sequence for estimation of NAHR breakpoint location. In artificial sequence, the S and E are special markers, for beginning and end of the sequence, L means that the observed sequence seems to come from the left (first) LINE, R means it comes from the right (second) one, N means that the source LINE cannot be determined from this location.

**Figure 5.**
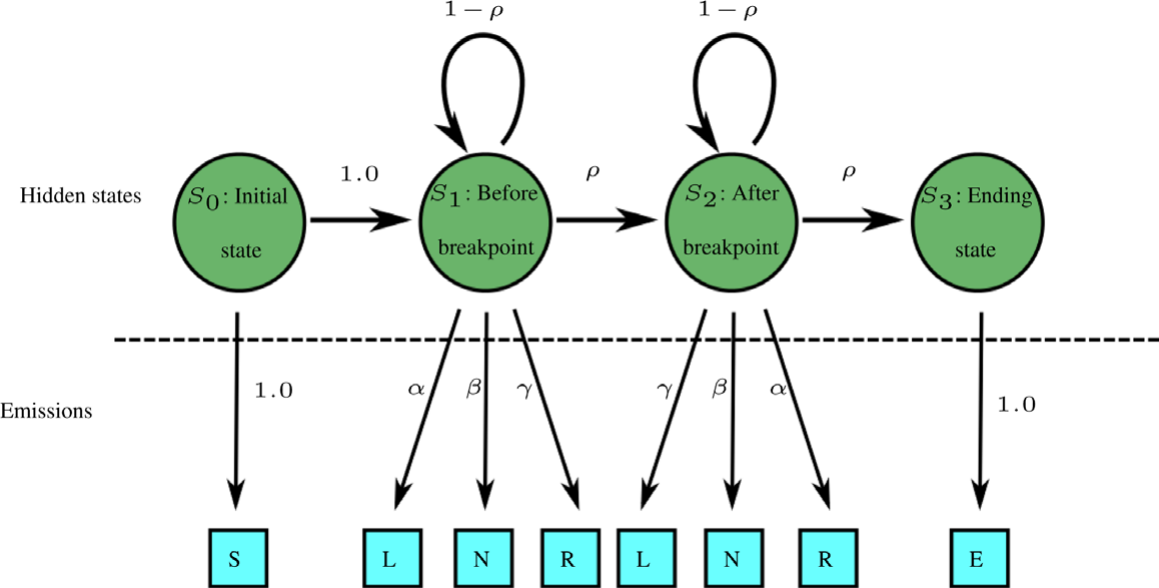
Hidden Markov model used for estimation of breakpoint location. The NAHR site maps at the point of *S*_1_ → *S*_2_ transition. The prior and posterior values of *α, β, γ, ρ* can be found in Supplementary Data.

For each pair of LINEs, a consensus sequence was computed, and a custom version of the Needleman–Wunsch algorithm (37), modified to compute a semi-global alignment, was used to align the Sanger reads to the consensus. An artificial sequence containing the information about sequence *cis*-morphisms was computed for each case (Figure 4). Then, the sequences were analyzed with a hidden Markov model (Figure 5) (38) trained using a custom version of the Baum–Welch algorithm (39) (see SI for algorithm details). The input sequence consists of the letters S, N, R, L and E and is constructed as shown in Figure 4)

The model with parameters obtained from the Baum–Welch algorithm were then used to compute the posterior probabilities of transition from the *S*_1_ state to *S*_2_ at all locations, which correspond to the probability that the NAHR cross-over event occurred at each location. The posterior probabilities were computed using a custom version of the forward-backward algorithm (40). Briefly, the modification involved replacement of the observation matrices corresponding to the L and R emissions with an affine combination of matrices for L and R with weights based on the PHRED quality score (41,42) of the sequence from which the L or R signals originated. In most cases, the posterior probability analysis identified a single location for the breakpoint. The computed locations were later confirmed by visual inspection using the Sequencher software.

### Enrichment of LINE pairs in CNV breakpoint regions

To assess whether the breakpoint uncertainty regions of CNVs identified in patients by clinical CMA are enriched for LINE pairs predicted to facilitate NAHR, we filtered the CNV database to remove duplicate entries (different patients with CNVs in the same region) and CNVs where the uncertainty regions of both breakpoints contained matching LCRs. This filtering prevents us from inadvertently counting CNVs segregating in the healthy population as independent events. We then computed the expected number, *ε*, of CNVs whose breakpoint uncertainty regions would contain a given LINE pair. This was calculated as the fraction of reference sequence that the proximal breakpoint uncertainty region occupies times the fraction that the distal uncertainty region occupies. Using our least stringent criteria for selection as a deletion/duplication mediating LINE pair, the average number of CNV breakpoint uncertainty regions expected to match is much less than one (*ε* = 0.058). We subsequently calculated the enrichment ($\mathcal {E}(l, {\rm id})$) of matching CNVs as a function of minimum LINE pair homology length *l* and sequence identity id:

\begin{equation*} \mathcal {E}(l, {\rm id}) = {{\#\rm{matched\_CNVs}(l, {\rm {\rm id}})}\over {\epsilon \cdot \#\mathrm{LINE\_pairs}(l, {\rm id})}} \end{equation*}

where $\#\mathrm{matched\_CNVs}(l, {\rm id})$ is the number of CNVs matched by LINE pairs with homology length of *l* or more and identity id or more, *ε* is the expected number of matching CNVs per LINE, and $\#\mathrm{LINE\_pairs}(l, {\rm id})$ is the total number of LINE pairs with homology of *l* or more identity id or more. The above formula is a simplified version of the actually used algorithm, which, also took into account border effects of CNVs lying near the edges of chromosome and centromeres. The resulting plot of the function $\mathcal {E} ()$ for varying values of *l* and *id* is shown in Figure 7.

## RESULTS

### Regions potentially prone to LINE–LINE-mediated NAHR

**Figure 6.**
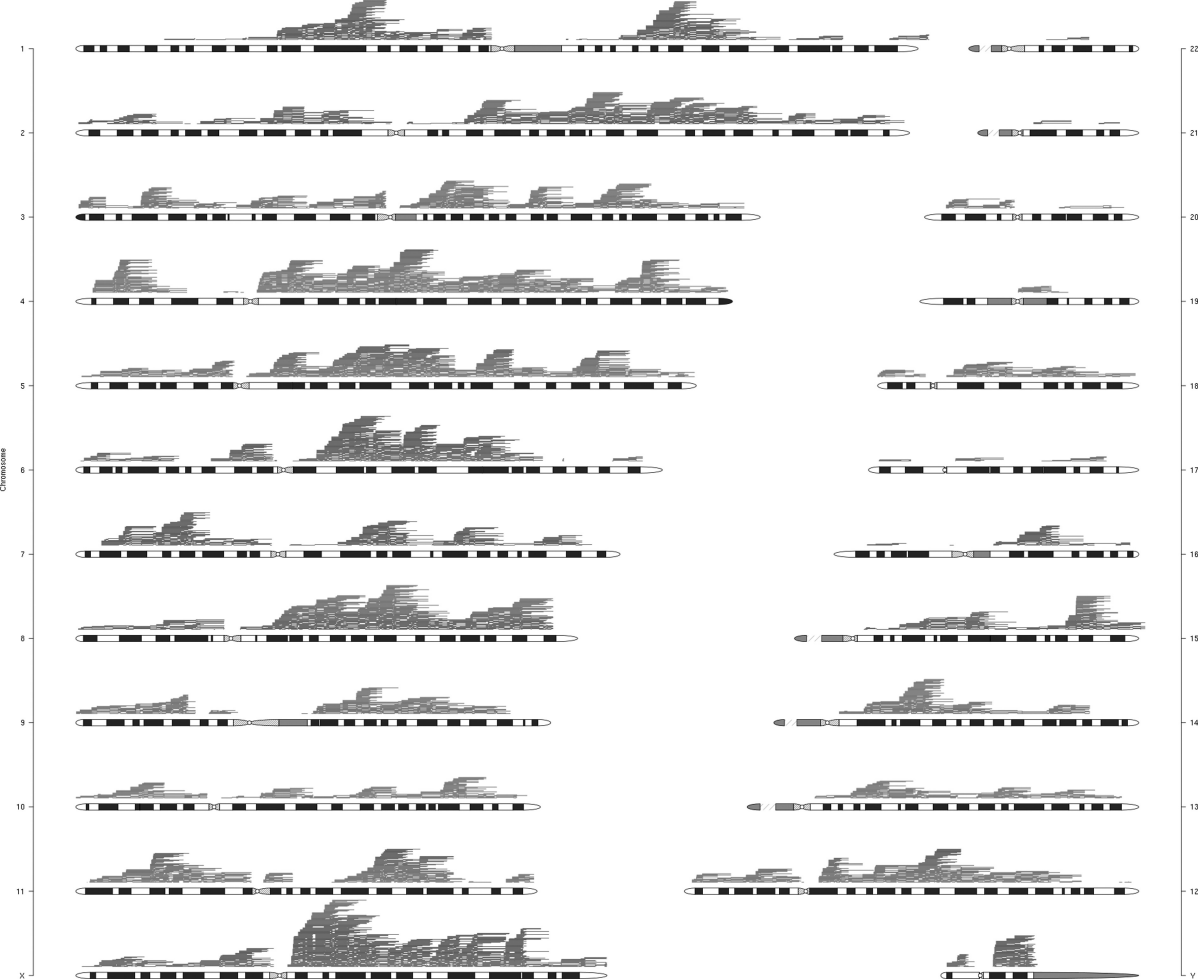
Ideogram showing potential susceptibility of human genome to LINE–LINE-mediated NAHR. Each horizontal line corresponds to one potentially NAHR-mediating LINE pair. The LINE elements map at the ends of the lines, whereas the segment covers the potentially deleted or duplicated regions. For clarity of the figure, inversions and translocations are not shown.

Because of the relative abundance of transposons in the human genome compared to LCRs, they have the potential to mediate NAHR between a wider array of loci, thus potentially posing a significant contribution to genetic instability. From our bioinformatic analyses of the genome, we found 416 180 potential deletion/duplication, 415 581 inversion, and 59 678 570 translocation sites. Figure 6 indicates the genomic regions potentially susceptible to deletions or duplications due to LINE–LINE mediated NAHR events. Our analyses suggest that 82.8% of the human genome is potentially susceptible to such events, with most of the genome being overlapped by multiple pair combinations.

Of note, the number of different potentially NAHR-mediating LINE–LINE pairs encompassing a given genomic locus (and thus, the probability of its variation in copy number) is dependent on the *square* of the density of homologous LINEs in its vicinity. Therefore, stochastically occurring clusters of LINE elements greatly increase the predicted instability of the genome in a given area.

### Analyses of LINE–LINE CNV breakpoints observed among individuals tested by clinical CMA

To further investigate LINE–LINE mediated NAHR, we cross-referenced our predicted LINE–LINE/NAHR susceptibility regions with a database of CNVs identified in patients tested in our clinical diagnostic lab. We focused additional attention on loci where two or more distinct patients had CNVs overlapping the same pair of LINE elements. In 44 cases, we successfully amplified the junction fragment of the putative LINE–LINE mediated CNV. Using Sanger sequencing, the location of each NAHR site was narrowed to a homology region between single base mismatches, one belonging to the proximal LINE and the other to the homologous distal LINE. In most cases, it was possible to pinpoint a single breakpoint location for a given patient both manually and using the probability plots obtained from the hidden Markov model (Supplementary Data). The NAHR breakpoints mapped to 80 ± 53 bp (excluding outliers) indicative of the highly identical nature of the LINE–LINE pairs (Supplementary Table S1). In 15 cases, the segment of perfect identity was much longer (2.4 ± 0.7kb). In two cases, the breakpoint could be determined to the basepair. Genomic coordinates of all identified breakpoints are listed in Supplementary Table S1.

### Enrichment of predicted NAHR-mediating LINE-LINE pairs in CNV breakpoint uncertainty regions

**Figure 7.**
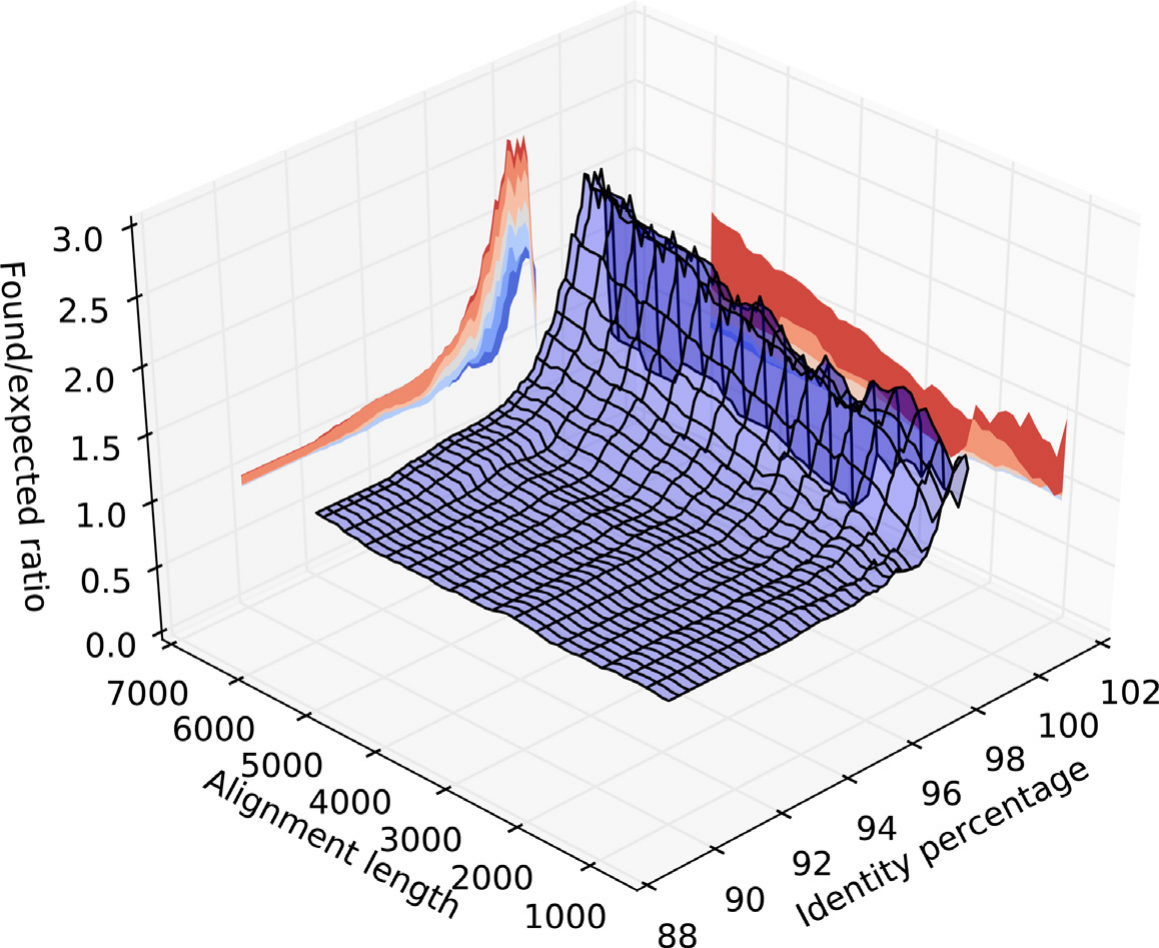
A plot of the observed/expected ratio of CNV breakpoint uncertainty regions containing LINE pairs fulfilling various homology length and identity criteria. LINE pairs with identity 96% are enriched at CNV breakpoints, suggesting that such pairs facilitate NAHR. Meanwhile, there is no sharp restriction on minimal length of the homology.

We also observed a significant enrichment in LINE/LINE pairs matching particular criteria in the breakpoint uncertainty regions of CNVs identified among patients tested in our diagnostic laboratory (Figure 7). Interestingly, the data suggest that an identity percentage of 97–98% or more between the homologous sequences is necessary to mediate NAHR, with 96% being the absolute minimum. On the other hand, it appears that there is no such sharp cutoff associated with the length of the homologous sequence, as homology of <1 kb appears to be enough to increase the likelihood of NAHR occurring. Thus, even fragmented copies of LINE elements may be capable of mediating NAHR.

### NAHR hotspots in flanking LINE elements

**Figure 8.**
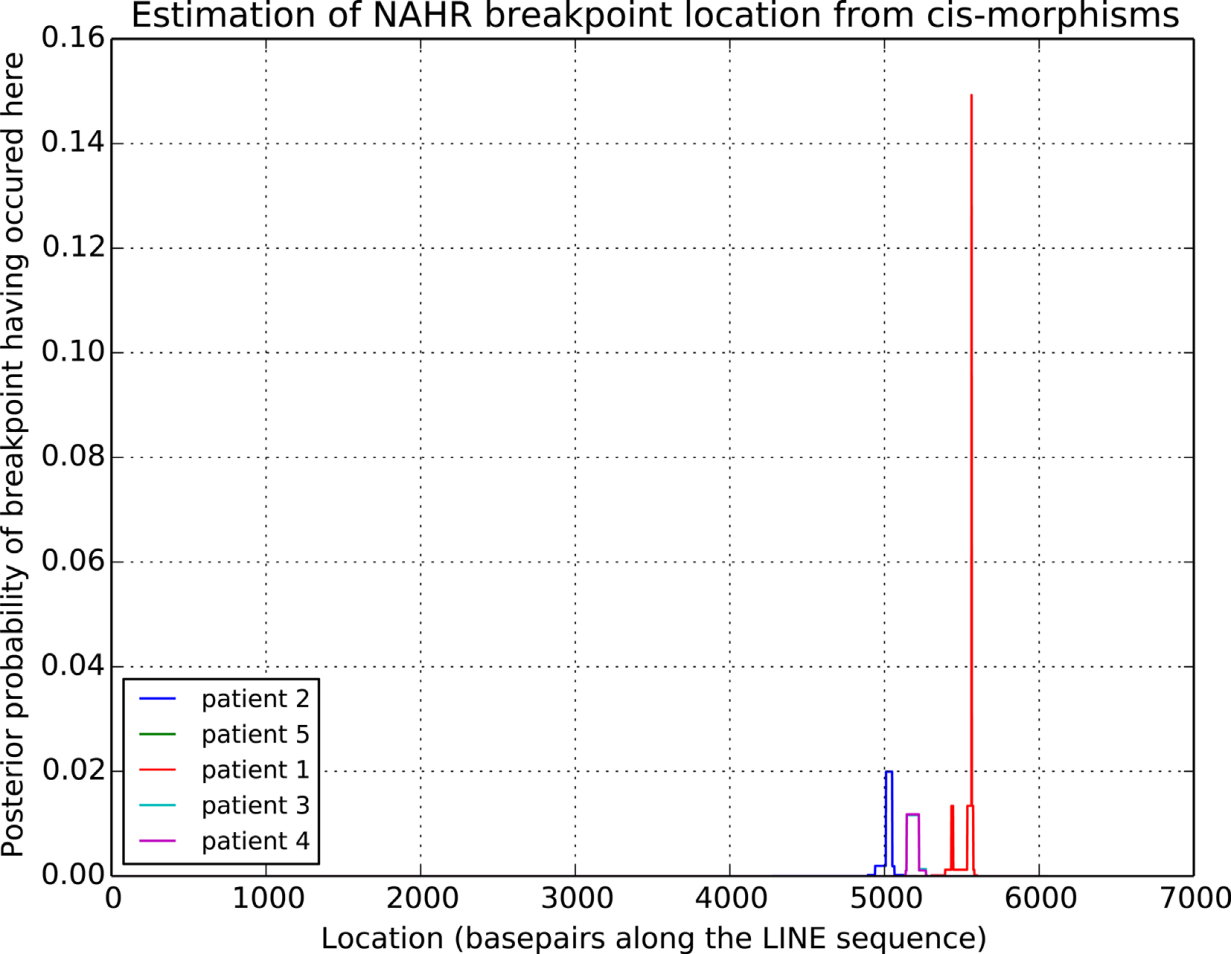
Estimated NAHR breakpoint location probabilities from the hidden Markov model for duplications between LINEs on chromosome 20 (see SI for precise locations). Three distinct NAHR loci were identified among the tested patients.

In some cases, the precise NAHR cross-over site in a given LINE–LINE pair varied between patients, for example, in the case of duplication on chromosome 20 (see Figure 8, and Supplementary Data additional loci), indicating independent *de novo* events. In all cases, the NAHR sites either mapped between the same two *cis*-morphisms or were clustered together, typically within 500 bp of each other. This observation suggests that inside the LINE elements there exist NAHR-facilitating motifs which make some regions of the LINE more prone to recombination than others. We performed computational analyses of potential hotspot motifs, including the canonical recombination motif associated with PRDM9 binding (43). We were unable to identify significant enrichments near the identified breakpoints for any of motif. However, it should be emphasized that the methods of motif discovery based on sequence similarity are not applicable to highly homologous sequences such as the LINE elements.

### aCGH analysis of healthy individuals

To address the contribution of LINE–LINE-mediated NAHR on genome variation in the healthy population, we performed high-resolution array CGH with probes flanking the LINE elements that we computationally predicted to contribute to genome instability on peripheral blood DNA from six healthy individuals. We identified 13 potential CNVs mediated by the LINE pairs predicted in our computational analysis (Figure 6). The CNVs identified in control individuals were small, each <25 kb, including the deleted or duplicated segment of LINE. At two loci, the CNVs involved intronic sequences of RefSeq genes. Each CNV identified in the healthy subjects overlapped similarly sized deletions or duplications in the Database of Genomic Variants, suggesting their widespread occurrence.

**Figure 9.**
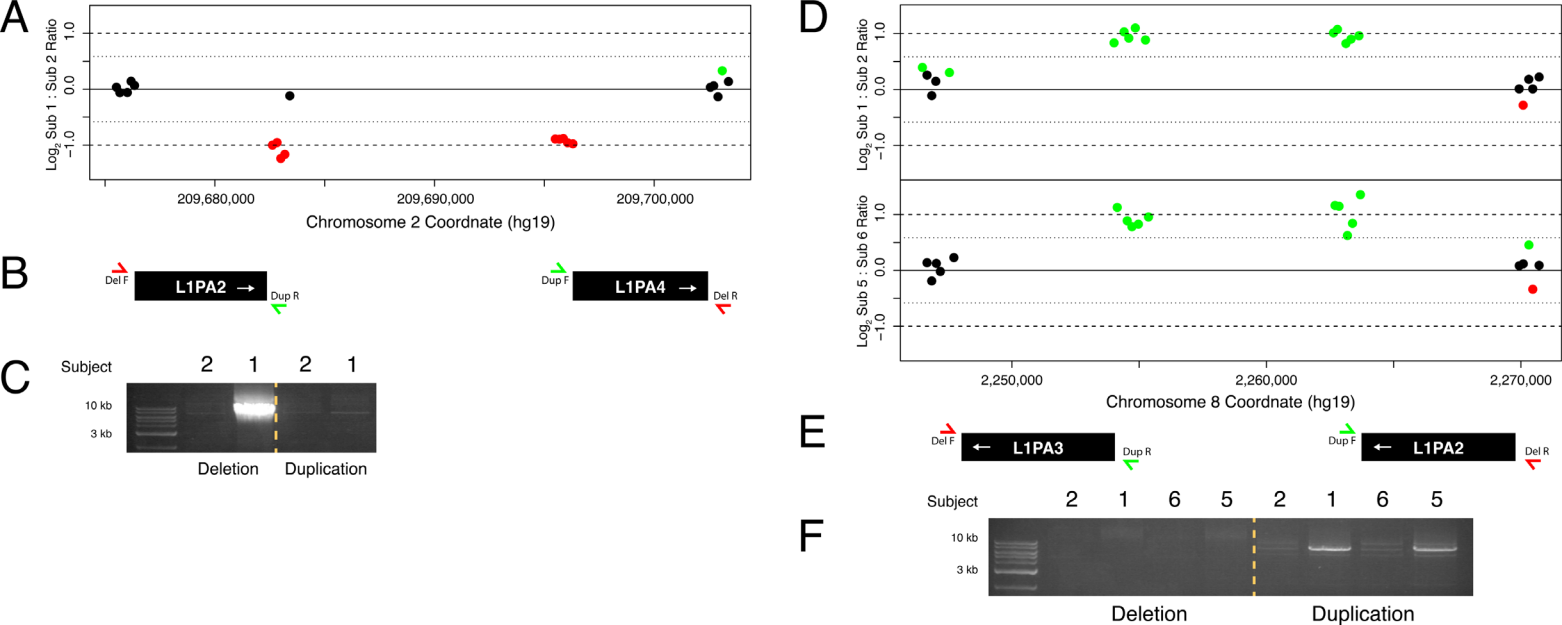
Molecular validation of predicted LINE–LINE CNVs identified among healthy individuals by array CGH. (**A**) Array CGH data indicates a CNV at 2q34 in subject 1 or 2. (**B**) Schematic representation of the L1PA elements that mediate the CNV and LR-PCR primers testing for the CNV. (**C**) LR-PCR identifies the presence of a deletion in subject 1. (**D**) Array CGH data indicates a CNV at 8p23.3 in subject 1 or 2 and subject 5 or 6. (**E**) Schematic representation of the L1PA elements that mediate the CNVs and LR-PCR primers testing for the CNVs. (**F**) LR-PCR identifies the presence of homozygous duplications in subjects 1 and 5.

Because genomic DNA from the healthy individuals was hybridized together, the aCGH data alone were insufficient to differentiate a deletion in one individual from a homozygous duplication in the other. For two loci (2q34 and 8p23.2) where the genomic architecture surrounding the CNV was conducive to unique primer design and long range PCR, we validated the CNVs molecularly (Figure 9). We used two pairs of primers at each locus to test for both deletions and duplications separately. The PCR revealed a heterozygous deletion in subject 1 at 2q24 and homozygous duplications in subjects 1 and 5 at 8p23.2. These data suggest that small LINE–LINE mediated CNVs are present in the normal population and are common enough to be present in the homozygous state. Thus, such CNVs likely contribute to normal genetic variation.

### LR-PCR analysis of healthy patients

In addition to array CGH analysis of healthy subjects, we also analyzed pooled genomic DNA samples from eight healthy subjects using LR-PCR. The analysis of 95 LINE–LINE pairs with strong homology parameters produced three to four bands of expected size (but lower intensity), for deletions, duplications and inversions each. After testing each DNA sample individually using repeat LR-PCR, similar weaker intensity bands were present in two to three individuals. Sanger sequencing of the products was inconclusive, potentially indicating PCR artifacts due to the repetitive nature of the LINE elements at the breakpoints.

## DISCUSSION

Here, we have performed a genome-wide study to determine the extent and frequency of NAHR events between LINE elements and assess the impact of such events on the genome and their potential to cause genetic disease in humans. Our results indicate that LINE–LINE-mediated NAHR does occur frequently and on a genome scale. It should be noted that the putative destabilizing influence of TE-mediated NAHR on the human genome has been previously underestimated, partially due the technical limitations of next generation sequencing methods used in studies of the human genome. Because of the abundance of TEs, structural variations with endpoints in TEs pose a particular challenge during mapping or *de novo* assembly. Such variations would much more frequently remain undetected compared to those arising due to LCR-mediated NAHR (44,45). This limitation is expected to be overcome using the massively parallel sequencing reads with lengths larger than the size of TEs.

Previous studies in yeast model organism indicated a similar mechanism of NAHR mediated by repetitive Ty elements. Ty elements are a small family of LTR retrotransposons, occurring at about 35 copies per haploid genome (46). Most of the chromosomal aberrations in yeast occur as a result of Ty–Ty recombination. Certain conditions were shown to stimulate NAHR between Ty elements such as dysfunctional replicative polymerase, partial deficiency in homologous recombination, DNA damage checkpoint response, or proximity of Ty element of chromosomal breaks (47–51). Given that enzymes mediating DNA repair and replication are well conserved in evolution it is very likely that similar mechanisms preventing NAHR between LINE elements are present in human cells.

We have molecularly confirmed apparent NAHR events occurring between elements with as little as 4 kb of DNA sequence homology. Furthermore, our statistical analyses (Figure 7) showed that LINE pairs with as little as 1 kb of homology are enriched at CNV breakpoint uncertainty regions.

Although we did not confirm all CNVs molecularly, the distribution of homology length and percentage of the putatively mediating LINE elements is consistent with previous reports of NAHR, suggesting that this indeed is the causative mechanism. The linear decrease of enrichment with decreasing length of the homologous fragment suggests both that the mechanism remains the same for longer and shorter fragments and that the probability of NAHR occurring is proportional to the length of the involved fragments. The lowest identity percentage of LINEs enriched at CNV breakpoints seems to be 95–96% for long fragments, and 96–97% for the short fragments. This in turn suggests that even for long DNA fragments, it might be a shorter sub-fragment with higher identity percentage than the average (over the whole LINE) that mediated the NAHR event, and that the actual identity percentage threshold for NAHR might be higher than the traditionally accepted 96%. Rather, in cases where it appears that a fragment pair with low identity percentage has mediated NAHR, instead a shorter subsequence with higher identity percentage was actually involved. However, it should be noted that this warrants further research, as the analysis we performed is not sufficient to draw conclusions with high confidence.

Our analyses of the breakpoints within sequenced individuals showed variability in the exact breakpoint locations, which suggests that at least some of the CNVs arose as independent events, rather than being inherited from a common ancestor. Moreover, we molecularly confirmed deletion and reciprocal duplication events at the same locus. These data demonstrate the destabilizing influence which the LINE elements exert upon the human genome, with NAHR as the most likely mechanism of this influence, rather than DNA replication errors. Thus, LINE elements contribute to human genetic variability by promoting NAHR in addition to well-described mechanisms of active retrotransposition (16,52). As we did not perform a study to differentiate between NAHR events arising during meiosis versus mitosis of germline cells in ancestor, we assume that the meiotic NAHR seems to be the most likely mechanism for the changes observed. A recent study, however, indicated that mitotic NAHR between LINE elements may be an under-recognized alternative mechanism (17). Moreover, apparently mitotic LCR-mediated NAHR events have also been reported in the literature (53,54).

The scale of such influence appears to be considerably higher than the influence of HERV elements (9), It is apparent that LINE elements should be considered as one of the major genomic features responsible for NAHR events, in addition to those already known (7). We estimate that each healthy individual carries on average three different LINE-mediated NAHR CNVs.

On the other hand, in contrast to LINE and HERV elements, *Alu*–*Alu*-flanked (55) genomic rearrangements have been reported much more often in the literature (21,24,56–59). They have been initially also attributed to NAHR (60); however, currently they are thought to be rather the products of DNA replication errors, such as fork stalling and template switching (FoSTeS)/microhomology-mediated break-induced replication (MMBIR) (61), or homeologous recombination (HeR)-mediated mechanism (58).

The clustering of NAHR breakpoints in particular hotspots within LINE elements suggests that at least some LINEs may carry a recombination-promoting sequence motif or structure; however, our current analysis failed to identify a statistically significant enrichment of any recombination motifs within the LINE elements tested in this study. Such clustering was proposed by previous studies of NAHR (24,62,63).

Our findings may have significant consequences for population genetics studies concerning the role of transposons in evolution, particularly in differences between transposon behavior in sexual versus asexual species. One particularly interesting possibility is that transposons could have been co-opted during evolution of sexuality to spread recombination sites through the genome. Another noteworthy aspect is that most population genetics models of activity of transposons assume that transposons exert a mutagenic influence only through active transposition. Here, we show that even inactive transposons may contribute to genome instability through NAHR. Such influence should be taken into account during population genetics modeling.

Summarizing, we have performed a genome-wide study of potential of LINE elements that mediate NAHR. The presence of numerous LINE–LINE-mediated rearrangements, both in clinical cases and in healthy individuals, indicates that NAHR mediated by LINEs occurs much more frequently than previously appreciated. We also provide several new bioinformatic algorithms for the study of NAHR (and particularly transposon-mediated NAHR). A database of genomic locations vulnerable to LINE–LINE NAHR has been computed and is included in Supplementary Data.

## SUPPLEMENTARY DATA

Supplementary Data are available at NAR Online.

SUPPLEMENTARY DATA
