# Supplementary material for: Genome-wide analyses of LINE–LINE-mediated nonallelic homologous recombination
Source: Nucleic Acids Res. 2015 Jan 22;43(4):2188–98. doi: 10.1093/nar/gku1394 (PMC4344489; doi:10.1093/nar/gku1394)
Supplement: SUPPLEMENTARY DATA [file supp_43_4_2188__index.html]

Genome-wide analyses of LINE–LINE-mediated nonallelic homologous recombination — Genome-wide analyses of LINE–LINE-mediated nonallelic homologous recombination — SUPPLEMENTARY DATA 

# Genome-wide analyses of LINE–LINE-mediated nonallelic homologous recombination

## SUPPLEMENTARY DATA

**Files in this Data Supplement:**

- SUPPLEMENTARY DATA
