# Supplementary material for: Genome-wide analyses of LINE–LINE-mediated nonallelic homologous recombination
Source: Nucleic Acids Res. 2015 Jan 22;43(4):2188–98. doi: 10.1093/nar/gku1394 (PMC4344489; doi:10.1093/nar/gku1394)
Supplement: SUPPLEMENTARY DATA [file supp_gku1394_nar-02523-d-2014-File003.pdf]

Published online *Nucleic Acids Research, , Vol. , No.* ***1–47***
doi:10.1093/nar/gkn000

Genome-wide analyzes of LINE-LINE-mediated nonallelic homologous recombination - supplementary information

Michał Startek [1,*], Przemyslaw Szafranski [2,*], Tomasz Gambin [2], Ian M. Campbell [2], Patricia Hixson[2], Chad A. Shaw [2], Paweł Stankiewicz [2,†], and Anna Gambin [1,3†]

[1]Faculty of Mathematics, Informatics, and Mechanics, University of Warsaw, 2 Banacha street, 02-097 Warsaw, Poland [2]Department of Molecular and Human Genetics, Baylor College of Medicine, One Baylor Plaza, Houston, Texas 77030, USA [3]Mossakowski Medical Research Centre, Polish Academy of Sciences, 5 Pawińskiego street, 02-106 Warsaw, Poland

REVERIFICATION OF NAHR BREAKPOINT LOCATION IN INDEPENDENT PCR REACTIONS TO RULE OUT THE POSSIBILITY OF A PCR ARTIFACT OCCURRING

We elected to perform multiple PCR amplifications across deletion breakpoints on chromosomes 5 and 20 in different patients with the primer sets different than in the original experiments (Fig. S1). We repeated the amplifications four times and Sanger sequenced the obtained amplicons. In all cases, the junction fragments were the same as in the original experiments (Figs S2 and S3). The product bands were single, strong, and of the expected sizes (as they were with the original primers) as opposed to weaker and diffused bands that would be expected from artifactual amplifications. These data strongly indicate that the amplifications are PCR primer specific and do not represent artifacts caused by mis-priming with nascent LINE amplicons. In addition, the presence of the analyzed nonmosaic CNVs was initially identified using an independent method - array CGH (Fig. S4). Moreover, we have previously reported multiple cases of constitutional CNVs mediated by LINE, HERV, or Alu repetitive elements (e.g. (1, 2, 3, 4, 5, 6, 7, 8))

*Authors contributed equally to this work.
†To whom correspondence should be addressed. Email: pawels@bcm.edu, aniag@mimuw.edu.pl

© The Author(s)
This is an Open Access article distributed under the terms of the Creative Commons Attribution Non-Commercial License (http://creativecommons.org/licenses/by-nc/2.0/uk/) which permits unrestricted non-commercial use, distribution, and reproduction in any medium, provided the original work is properly cited.

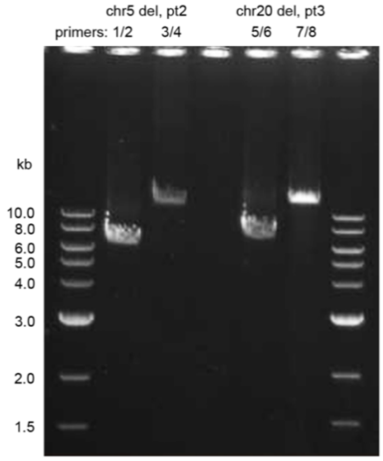

Figure S1. Results of the repeated PCR amplifications of junction fragments in patients with LINE-LINE-mediated genomic deletions on chromosomes 5 in patient 2 and 20 in patient 3 with the original (old 1/2 and 5/6) and new (3/4 and 7/8) primers, respectively. The PCR products amplified with the new primers were ~7 kb longer than the original amplicons.

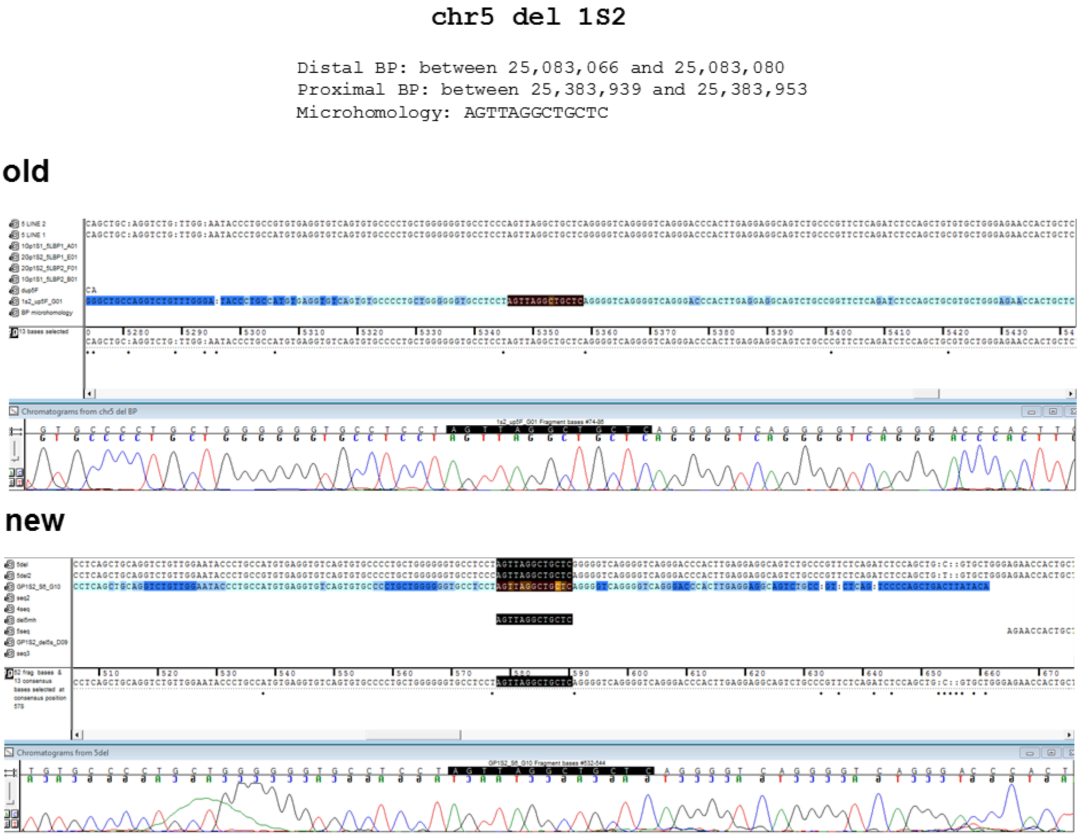

Figure S2. Comparison of the DNA sequences across the breakpoint junctions amplified with the original and new, different primers in patient 2, with deletion on chromosomes 5. The newly obtained breakpoint position is identical to the one sequenced for the original manuscript submission.

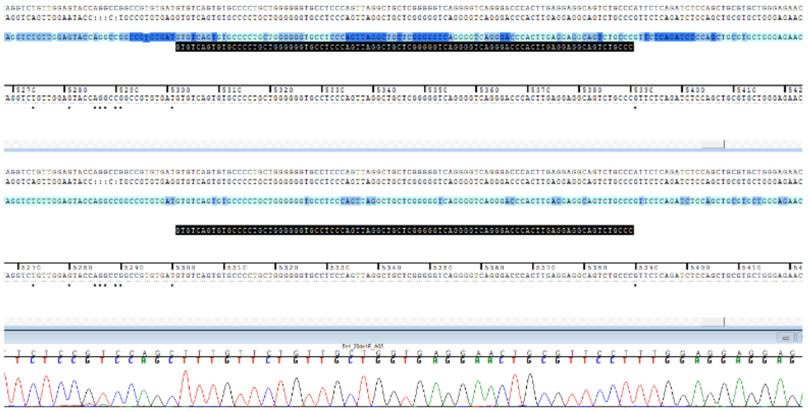

Figure S3. Comparison of the DNA sequences across the breakpoint junctions amplified with the original and new different primers in patient 3, with deletions on chromosomes 20. Again, the newly obtained breakpoint position is identical to the one sequenced for the original manuscript submission.

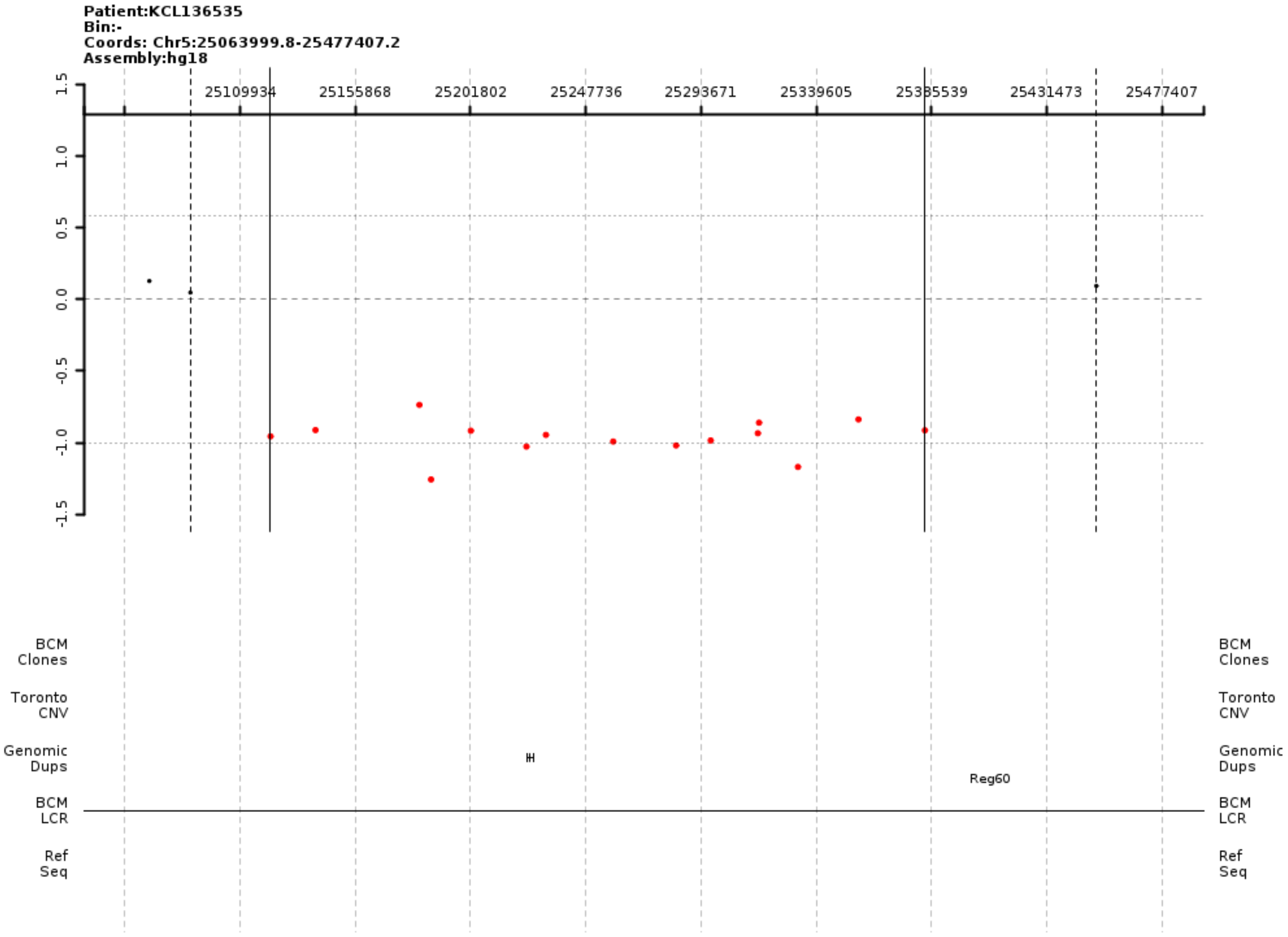

Figure S4. Array CGH plot of the investigated constitutional deletion on chromosome 5 in patient 2.

DATABASE OF POTENTIAL SITES OF LINE-LINE NAHR

The database is available for download at `http://bioputer.mimuw.edu.pl/~mist/LINEs/matching_LINE_pairs.csv.xz` (1.4GB, md5 checksum: 8cc5273038f45f9899667f067e67e686)
(warning: the above file uncompresses to over 15 GB!)

The much smaller, partial database containing only sites of intrachromosomal NAHR may be found at `http://bioputer.mimuw.edu.pl/~mist/LINEs/matching_LINE_pairs_intrachromosomal.csv.xz` (60MB, md5 checksum: 795ec6b64dbc5211676d9b9fd3475d38)

The files may be uncompressed using the standard Unix xz utility, or, on Microsoft Windows, with `http://www.7-zip.org/` software.

The above files are in CSV format, with the following columns:

aln_len	Length of the BLAST alignment between the two mediating transposons, only cases where this is greater than 1000 are included
distance	Genomic distance between the pair of mediating transposons in case of intrachromosomal rearrangements, meaningless otherwise.
eval	The E-value of BLAST alignemnt
gap_openings	Number of gap openings in the BLAST alignemnt.
hsp1s	Start of first BLAST HSP in genomic coordiantes
hsp1e	End of first BLAST HSP in genomic coordiates
hsp2s	Start of second BLAST HSP in genomic coordiantes
hsp2e	End of second BLAST HSP in genomic coordiates
idperc	Identity percentage of BLAST alignment, only cases where this is greater than 92.0 are included
matches	Number of mismatches in BLAST alignemnt
orientation	Orientation of the transposon pair. 1 is directly oriented, -1 is inverted.
q_end, q_start, query_id, s_end, s_start score, subject_id	internal
te1s	Start of the first interacting transposon [0]
te1e	End of the first transposon
te1_chr	Name of the chromosome containing the first transposon
te2s	Start of the second interacting transposon
te2e	End of the second transposon
te2_chr	Name of the chromosome containing the second transposon
type	Type of NAHR event that's suspected to be made possible by the transposons. Valid values: DELDUP, INVERSION, TRANSLOCATION

All listed coordinates are with respect to the HG19 genome assembly.

[0]Note: the HSP may be smaller then the transposon itself, it may also extend outside of the transposon. Cases where the HSP extends *significantly* outside of the transposon are not included, as they indicate the duplication is a part of a larger LCR, and not the result of transposition

LINES IN THE HUMAN GENOME

There are 1,498,692 LINE elements annotated in the HG19 assembly of the human genome. Most of these are short, fragmentaric copies, with shortest of them being 11 bases long. We decided to focus our analysis on the longer elements: over 4000 base pairs for wet-lab analysis, and over 1000 base pairs for bioinformatics.

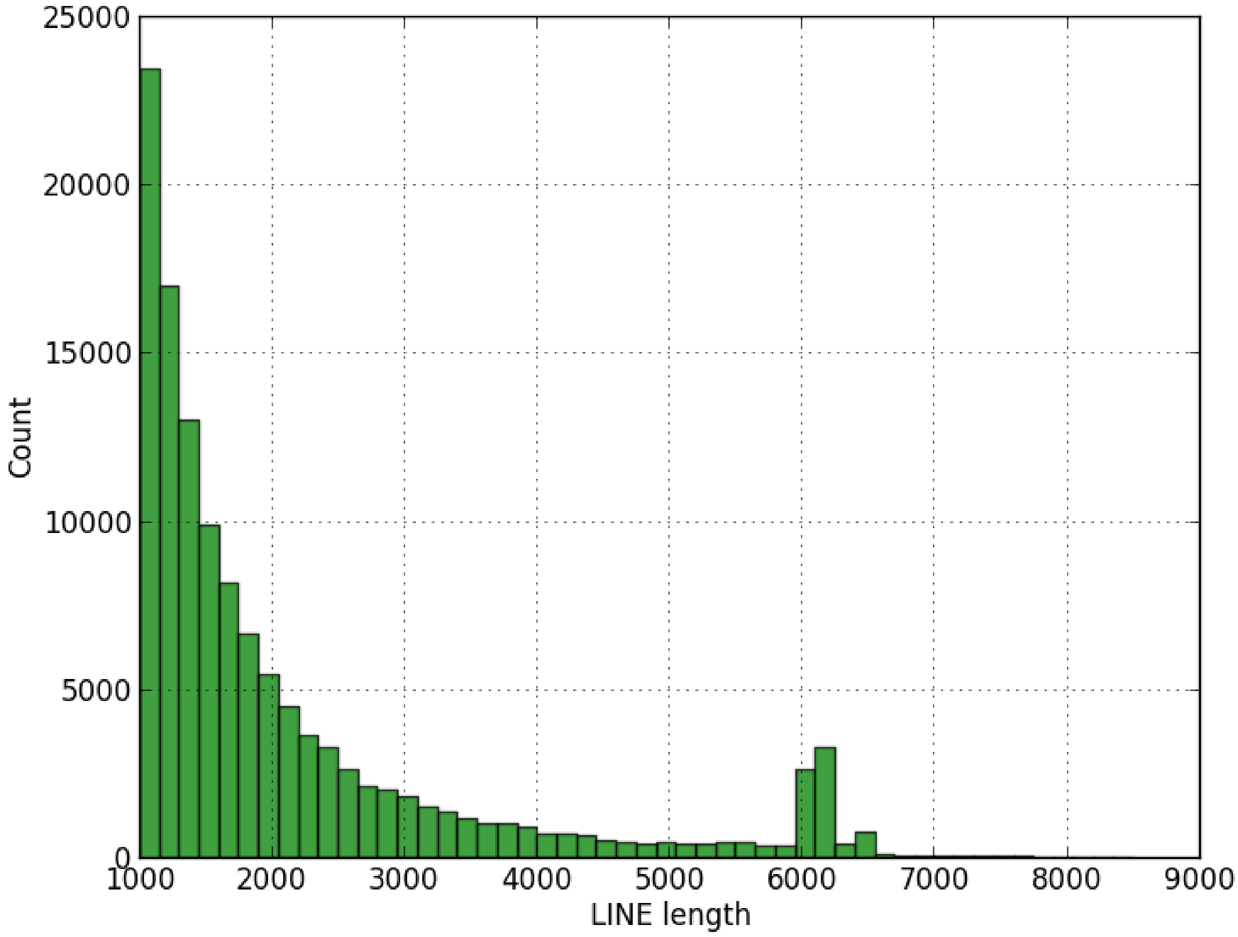

Figure S5. Histogram of of LINE element lengths greater than 1kb found in the human genome. The cluster around 6 kb corresponds to full-length LINE elements.

ALGORITHMIC PREDICTION OF BREAKPOINTS FROM SEQUENCING

For each pair of LINEs, a consensus sequence was computed, and a custom version of the Needleman-Wunsch algorithm (9) modified to compute a semi-global alignment was used to align the Sanger reads to the consensus. An artificial sequence containing the information about sequence *cis*-morphisms was computed for each case (Fig. S6). Then, the sequences were analyzed with a Hidden Markov Model (10) trained using a custom version of the Baum-Welch algorithm (11). The HMM has 5 hidden states: $S_0, S_1, ..., S_4$, the input alphabet is $\{S, N, L, R, E\}$, and the structure of the HMM is shown on Figure S7 The modified algorithm differs from the standard version in that it enforced the following constraints during training:

- $\mathbb{P}(S_1 \rightarrow S_2) = \mathbb{P}(S_2 \rightarrow S_3)$: ensures the model does not favour placement of breakpoints near the beginning or end of alignments because the training data happens to be skewed as such
- $\mathbb{P}(S_1 \text{ emits } N) = \mathbb{P}(S_2 \text{ emits } N)$,
  $\mathbb{P}(S_1 \text{ emits } L) = \mathbb{P}(S_2 \text{ emits } R)$,
  $\mathbb{P}(S_1 \text{ emits } R) = \mathbb{P}(S_2 \text{ emits } L)$: assumes that SNVs with respect to the reference sequence, which would make the source LINE ambiguous (such as Fig. S6, location 5), or even suggest the wrong LINE (location 6) are equally likely to occur on either side of the breakpoint.

The prior and posterior values for chain parameters are as follows:

Table 1.

Parameter name	Prior value	Posterior value
α	0.1	0.00924896713296794
β	0.89	0.9899202188834337
γ	0.01	0.0008308139835982798
ρ	0.05	0.00035456442623037844

The model with parameters obtained from the Baum-Welch algorithm were then used to compute the posterior probabilities of transition from the S_1 state to S_2 at all locations, which correspond to the probability that the NAHR cross-over event occurred at each location. These were computed using a custom version of the forward-backward algorithm (12), in which the observation matrices corresponding to the L and R emissions were replaced with an affine combination of matrices for L and R with weights based on the PHRED quality score (13, 14) of the sequence from which the L or R signals originated. The posterior probabilities were calculated, and in most cases a single location of the breakpoint was obtained. The computed locations were later confirmed by visual inspection using Sequencher software.

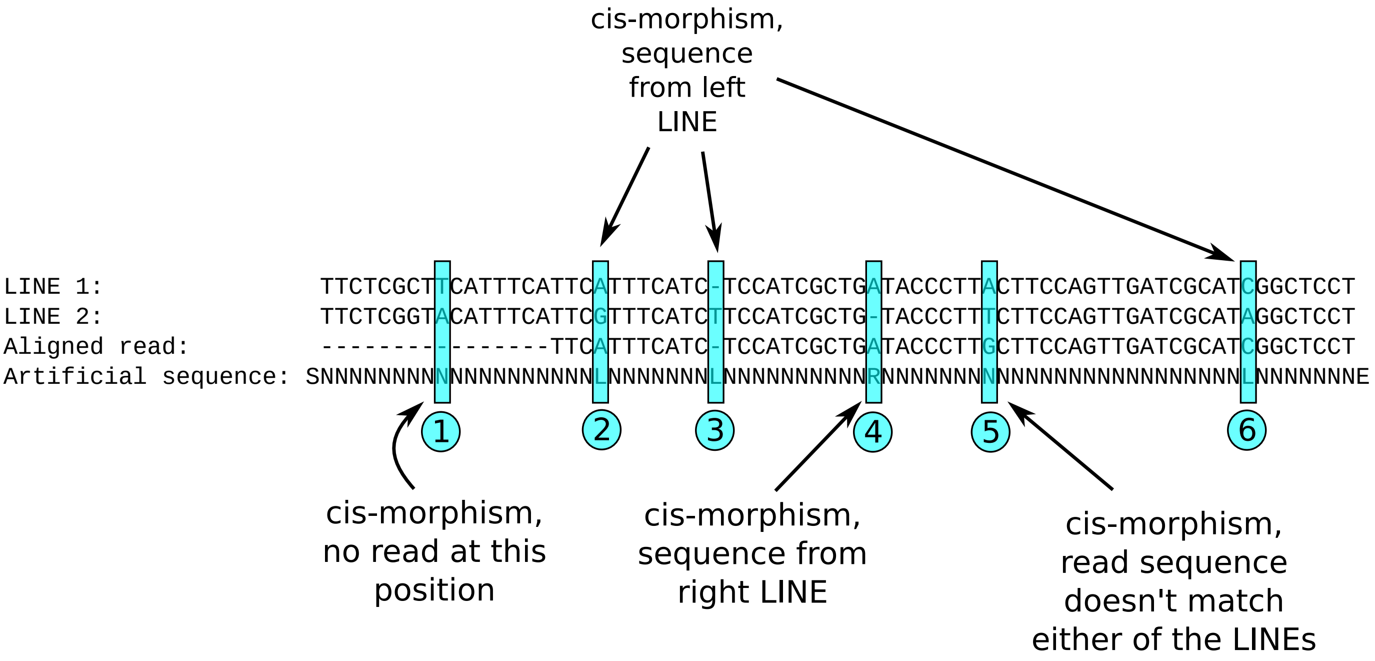

Figure S6. Construction of input sequence for estimation of NAHR breakpoint location. In artificial sequence, the S and E are special markers, for beginning and end of the sequence, L means that the observed sequence seems to come from the left (first) LINE, R means it comes from the right (second) one, N means that the source LINE cannot be determined from this location.

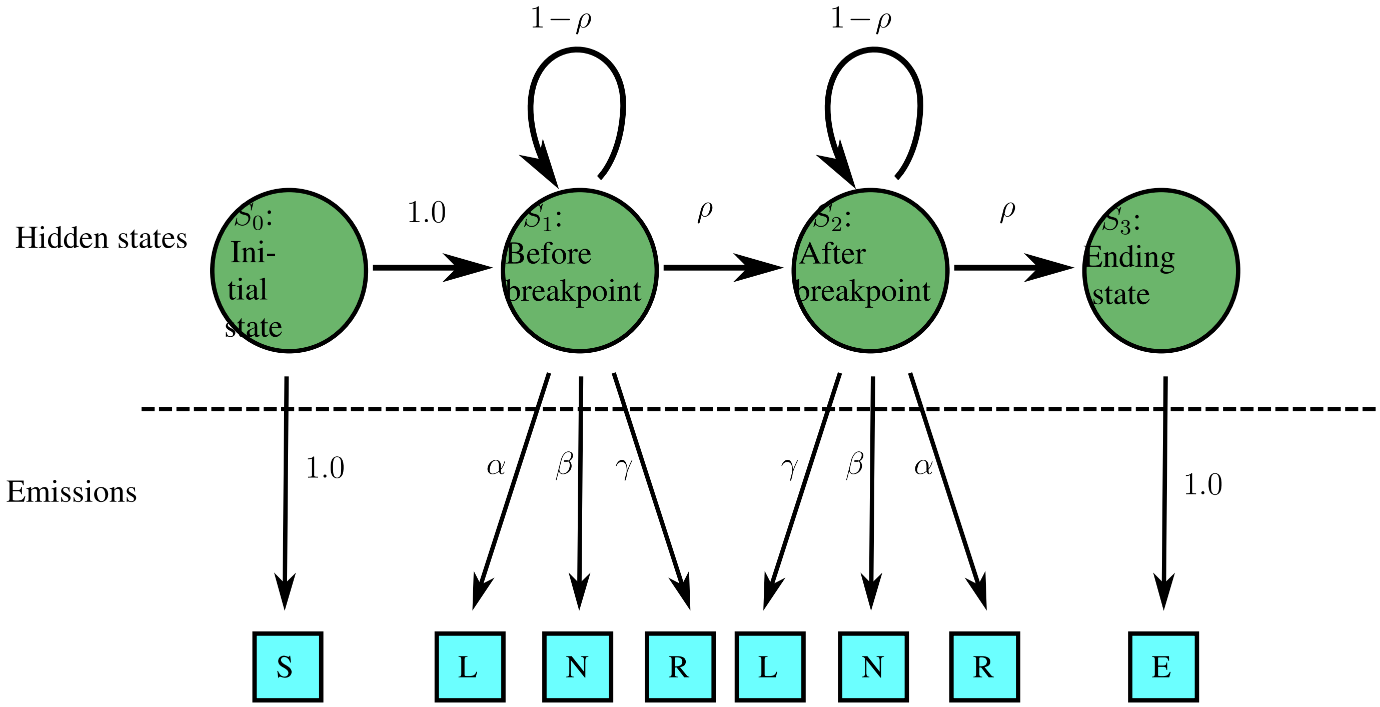

Figure S7. Hidden Markov model used for estimation of breakpoint location. The NAHR site maps at the point of $S_1 \to S_2$ transition. The prior and posterior values of α,β,γ,ρ can be found in Table 1.

ALGORITHMICALLY PREDICTED LOCATIONS OF FOUND BREAKPOINTS

Deletions, chromosome 5

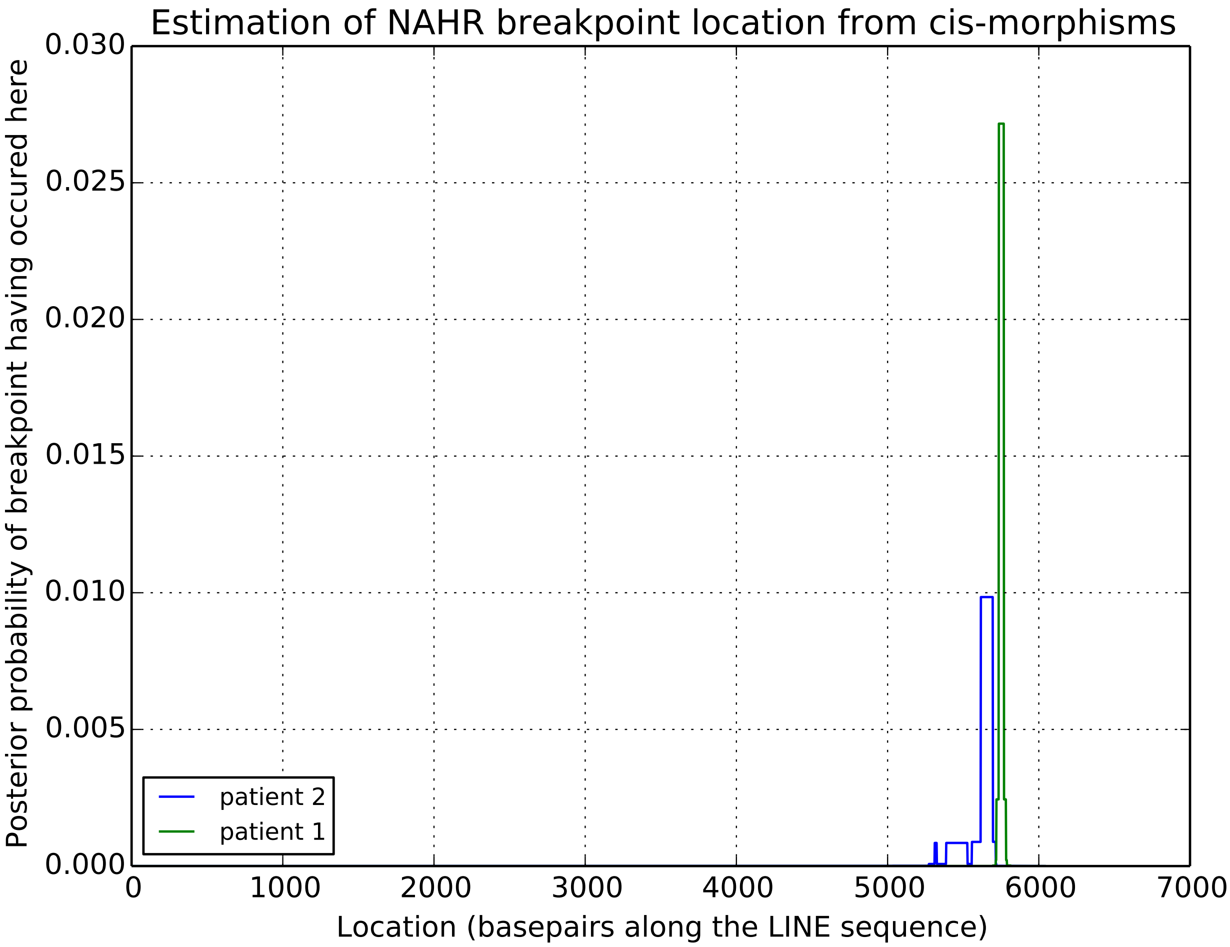

Deletions, chromosome 9

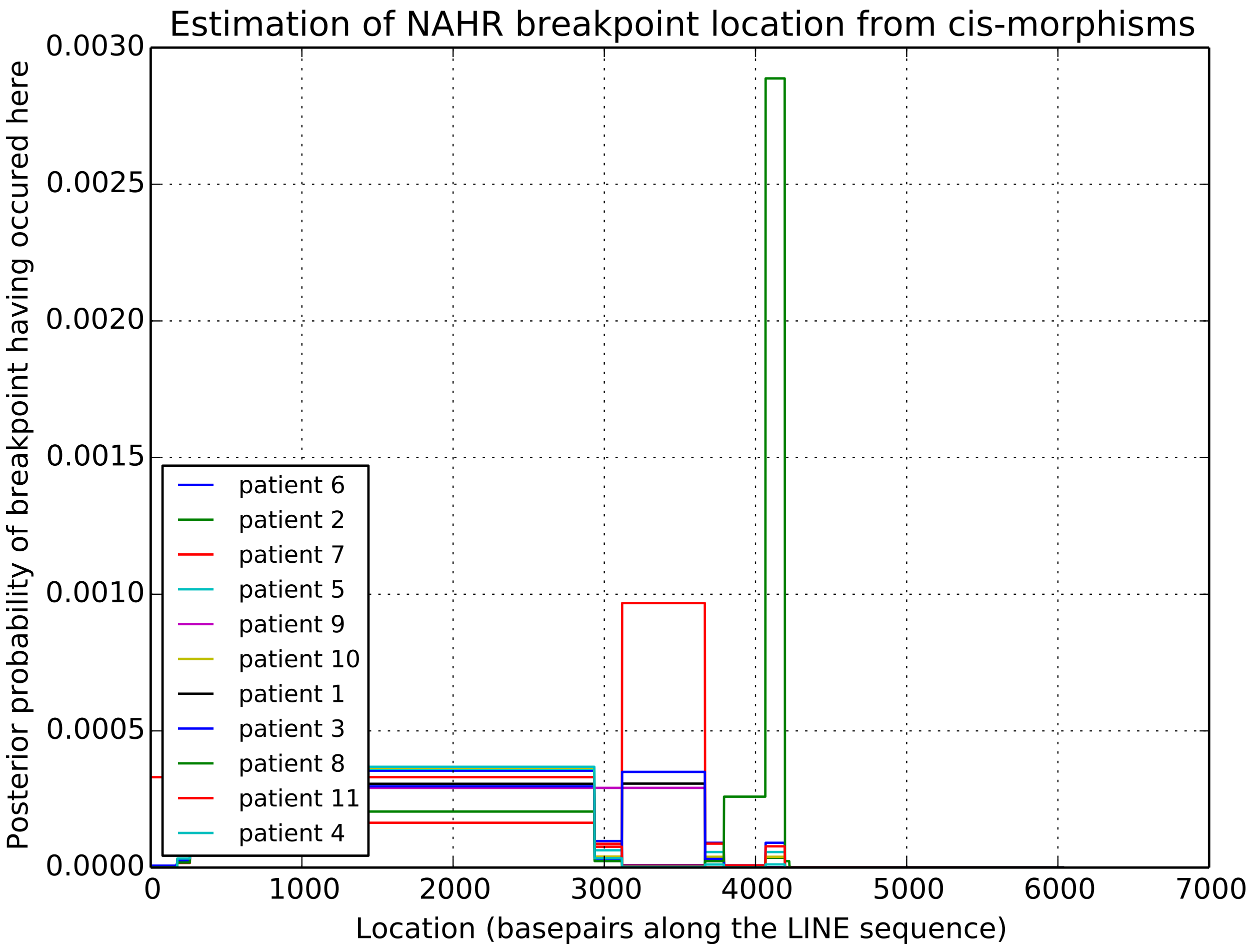

Deletions, chromosome 11

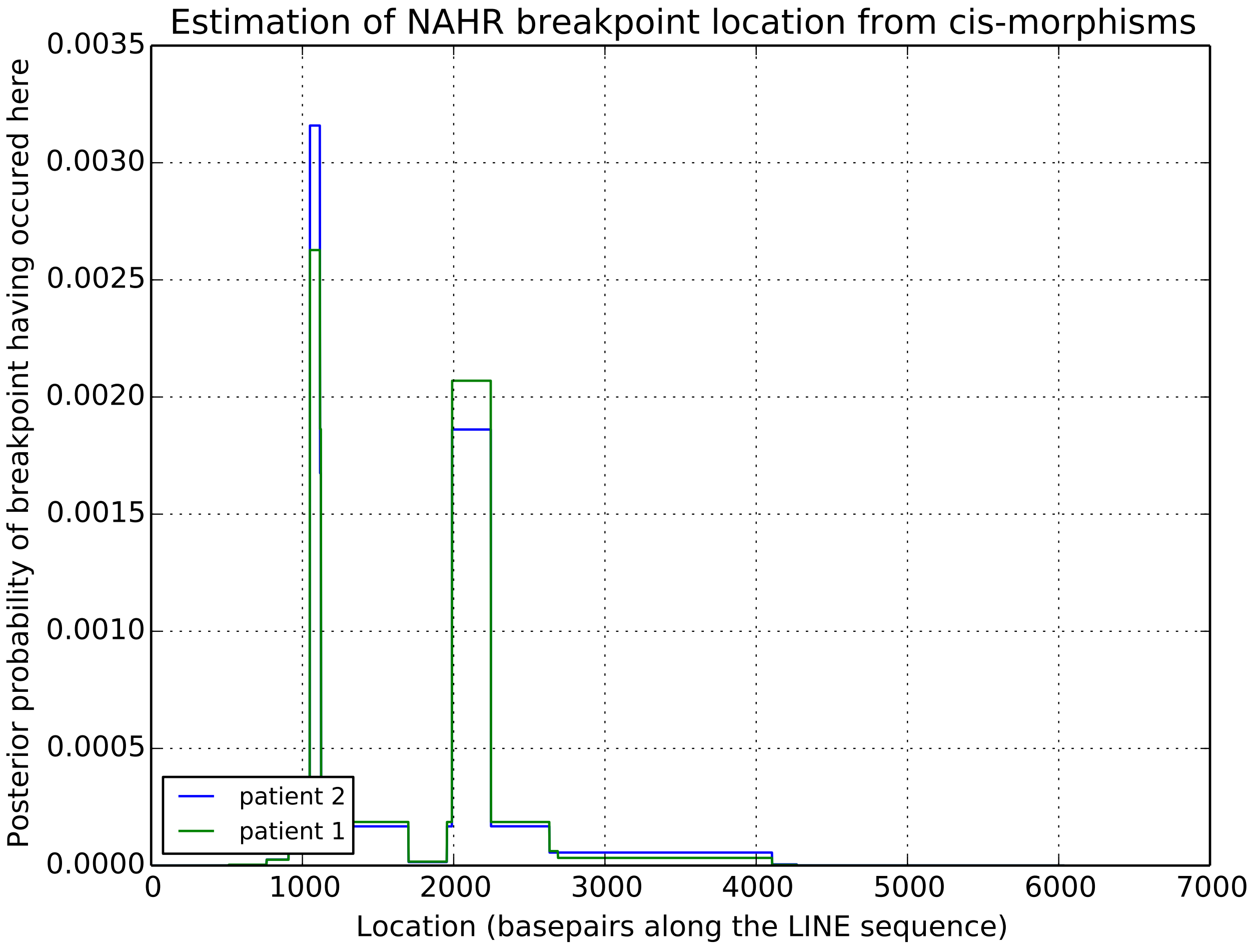

Deletions, chromosome 12

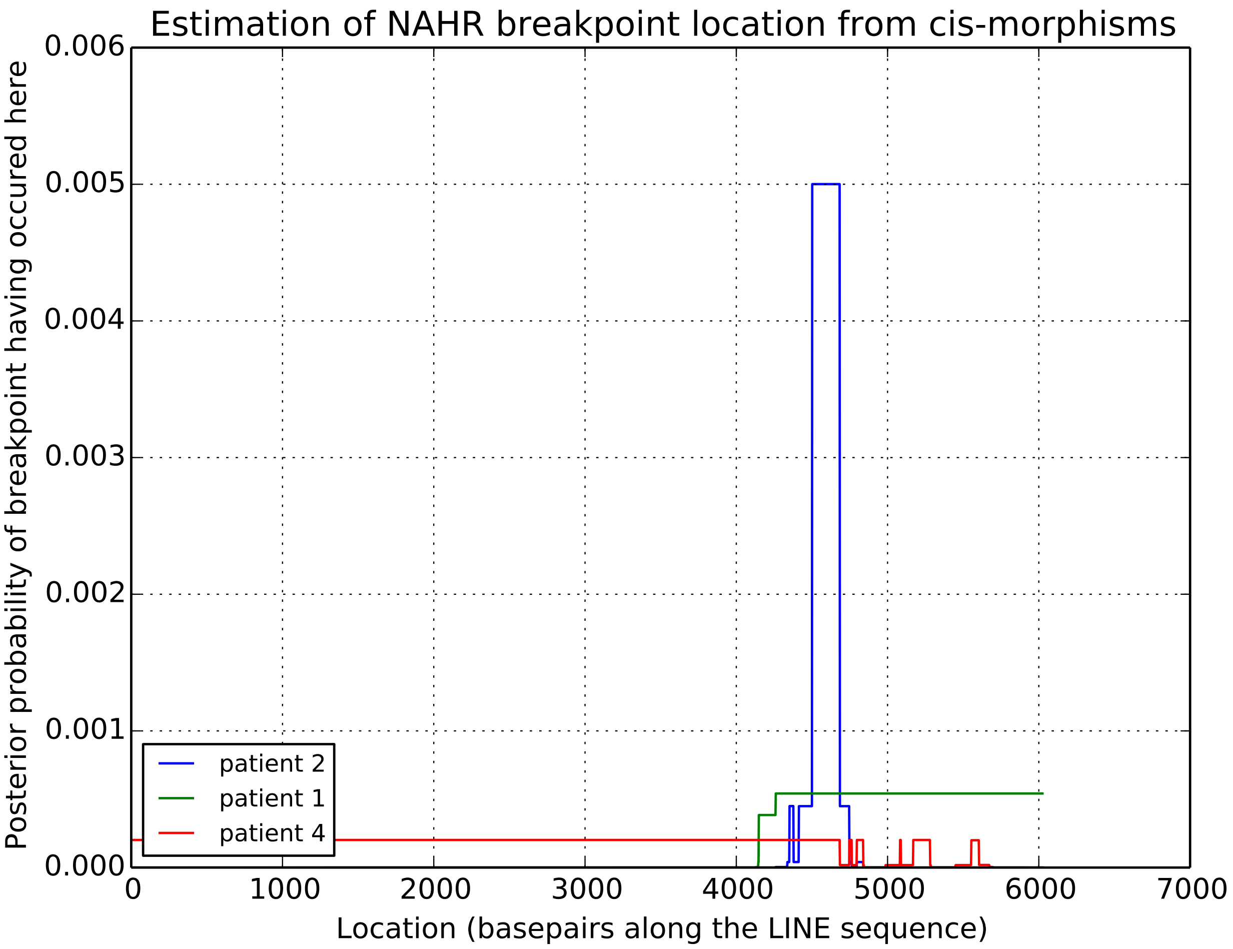

Deletions, chromosome 20

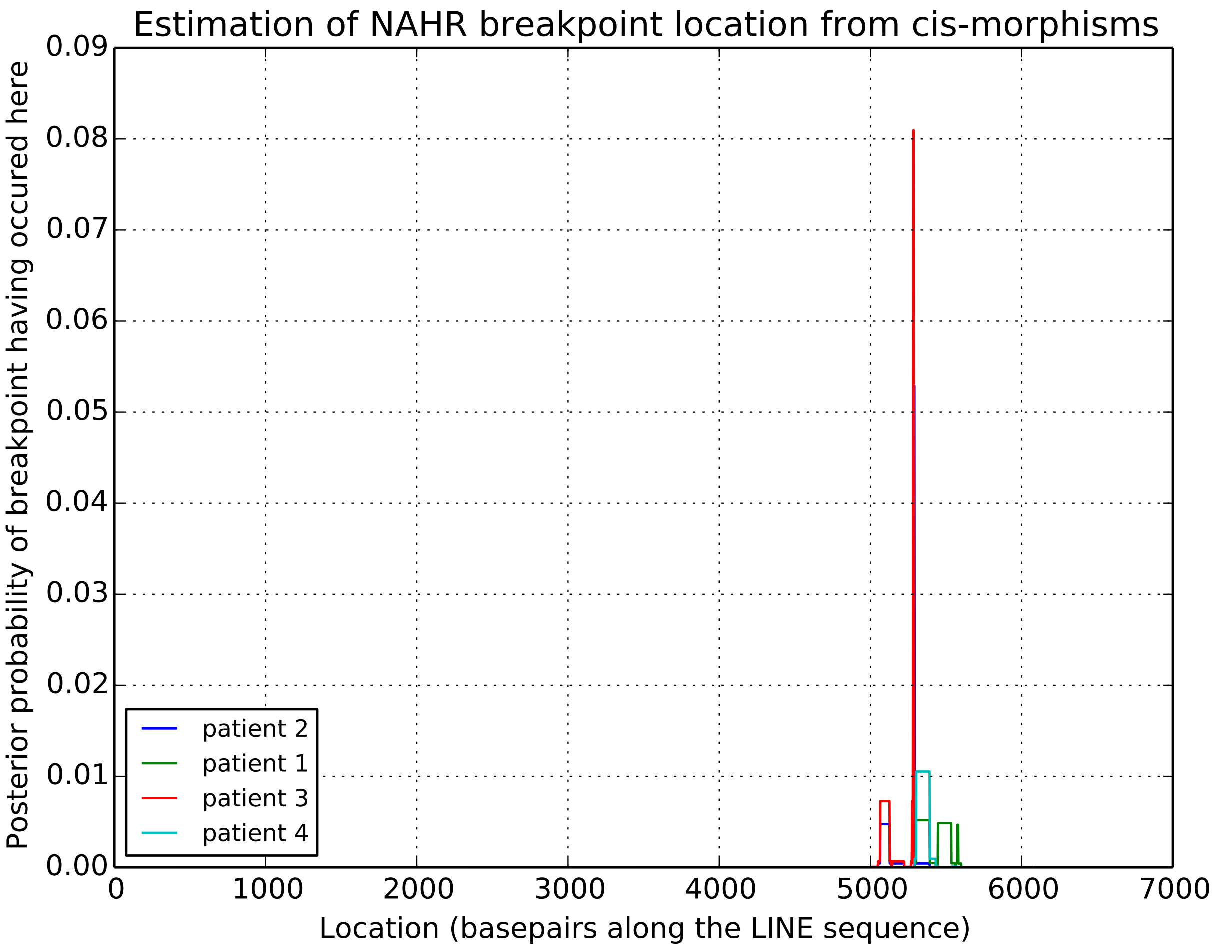

Duplications, chromosome 5

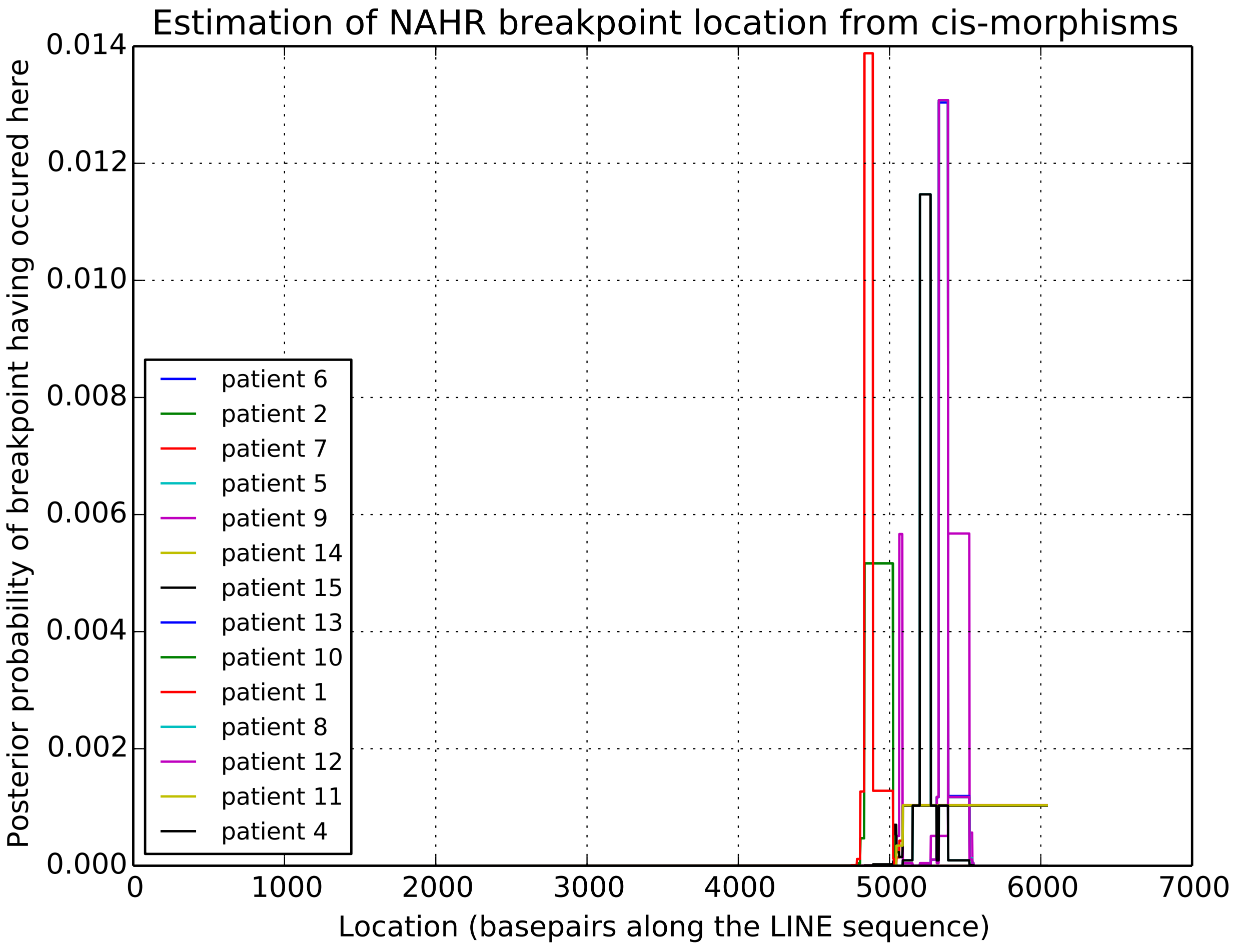

Duplications, chromosome 9

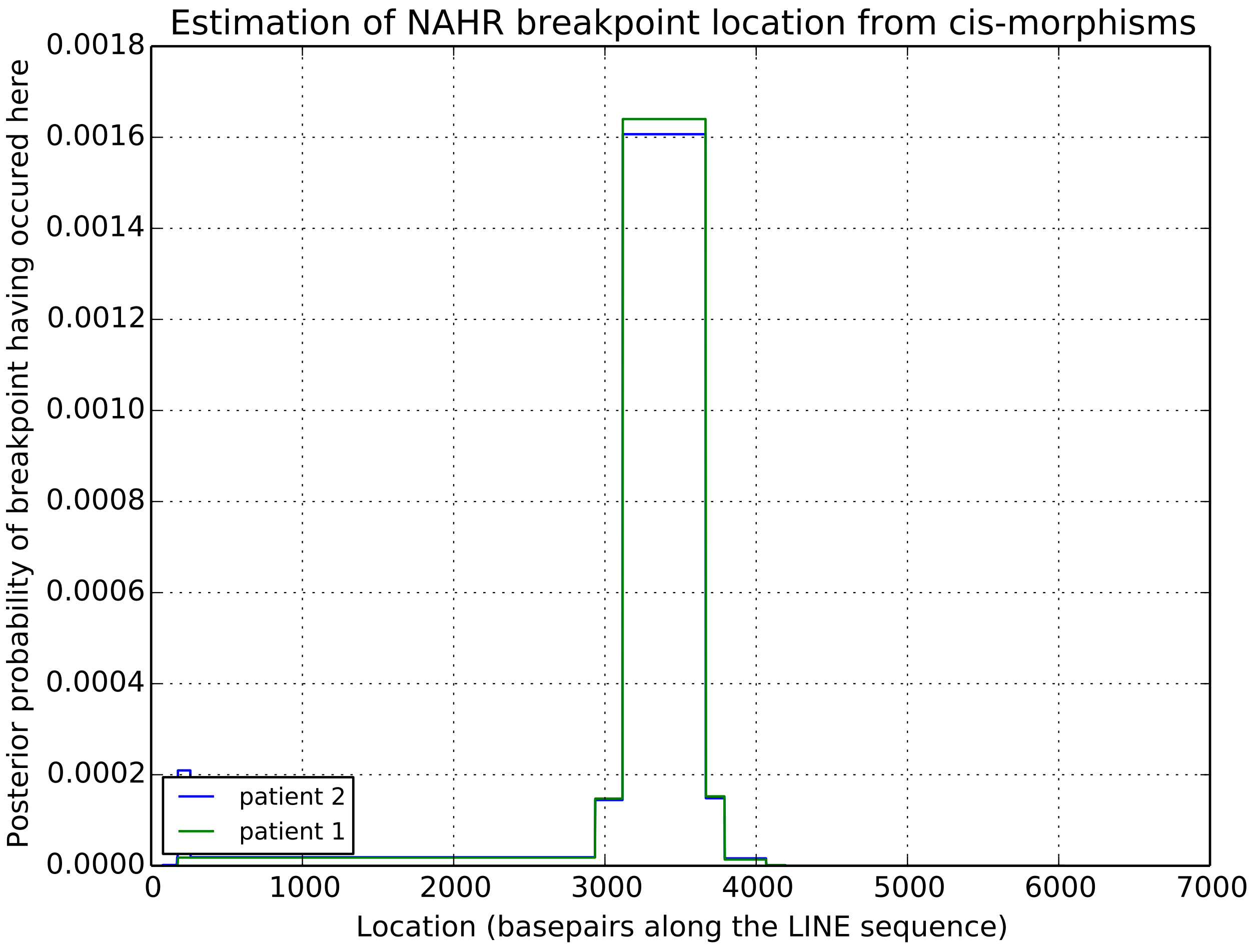

Duplications, chromosome 11

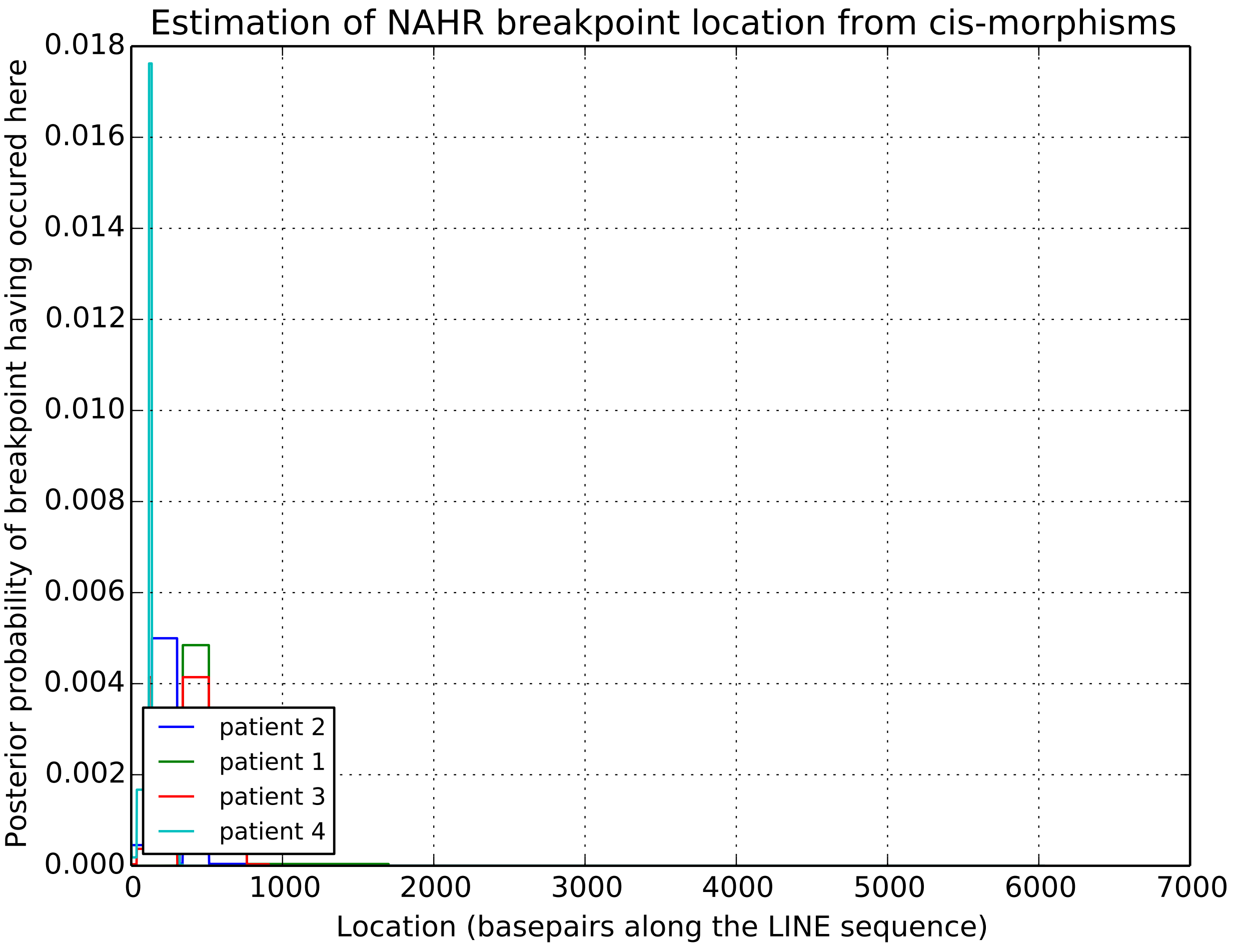

Duplications, chromosome 12

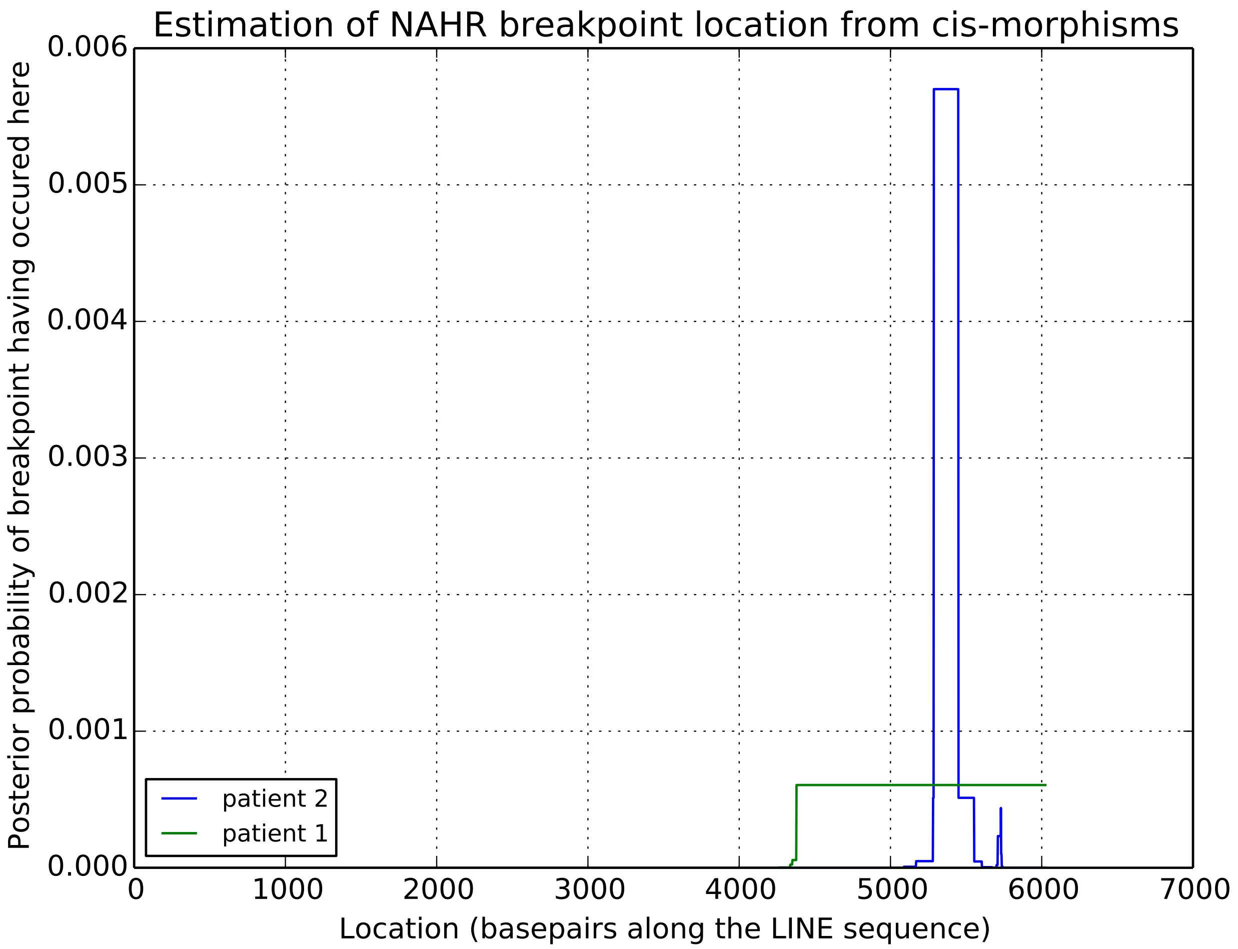

Duplications, chromosome 20

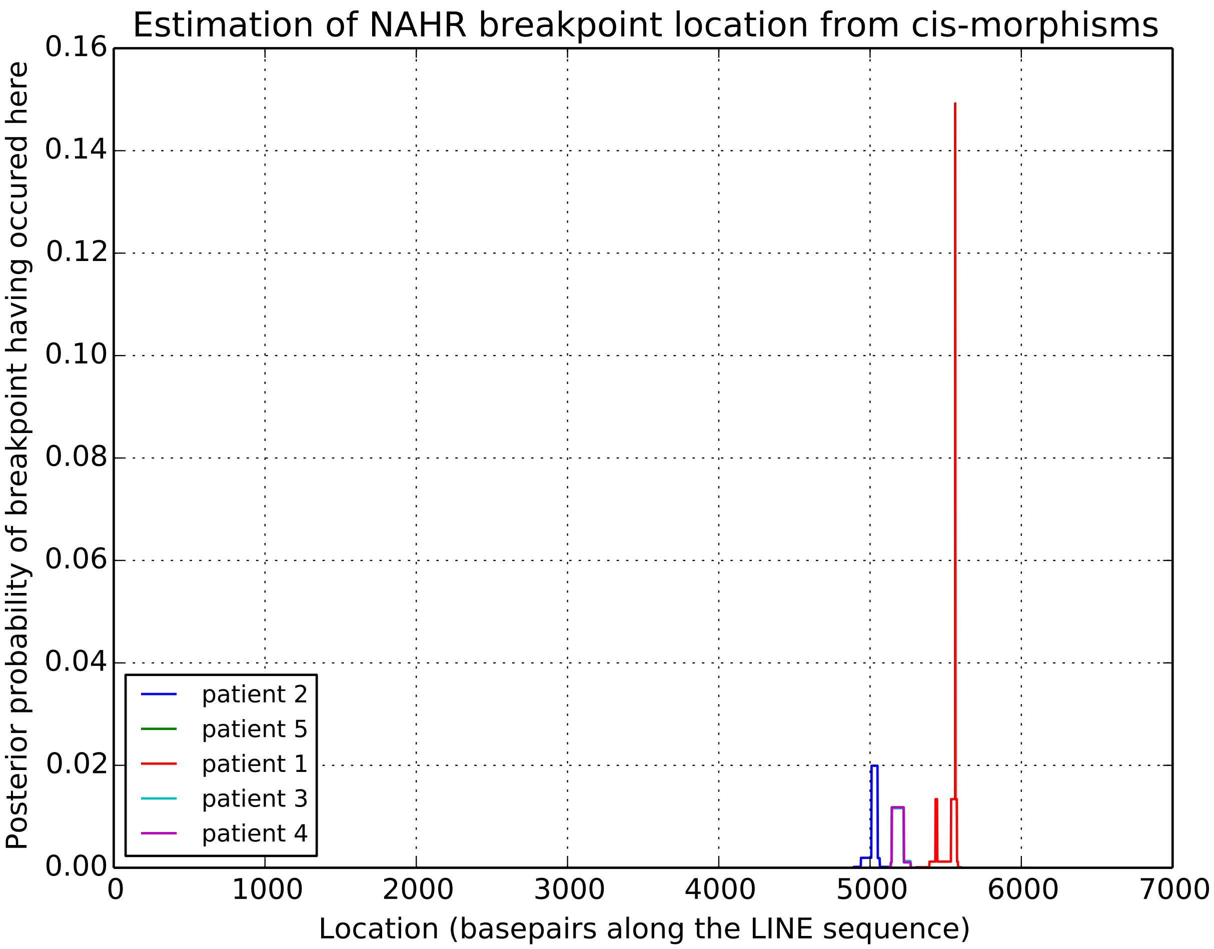

MANUAL VERIFICATION OF NAHR BREAKPOINT LOCATIONS

Below is the summary for manual double-check of the algorithmically found breakpoint locations.

Deletions

chr5 del patient 1

proximal BP: between 25,384,363 and 25,384,397
distal BP: between 25,083,490 and 25,083,524
microhomology: TCAGAAAAGCGCAGTATTCGGGTGGGAGTGACC

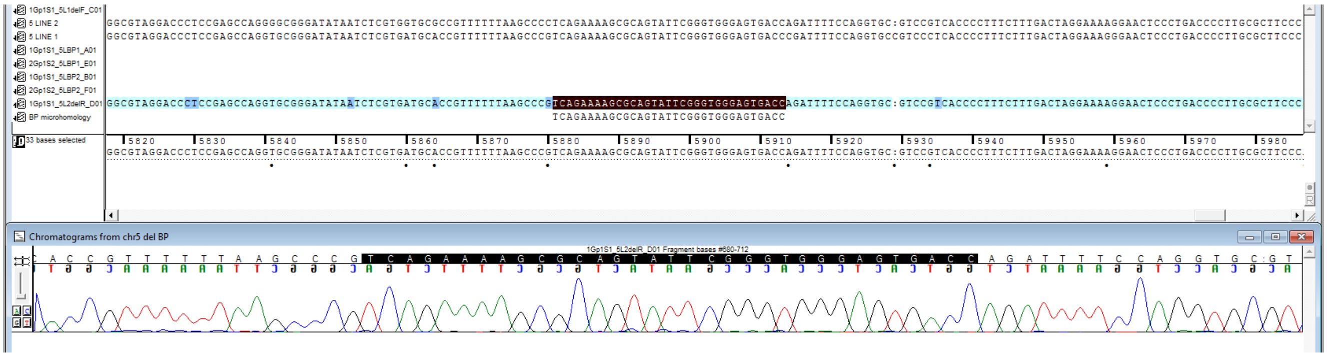

chr5 del patient 2

Distal BP: between 25,083,066 and 25,083,080
Proximal BP: between 25,383,939 and 25,383,953
Microhomology: AGTTAGGCTGCTC

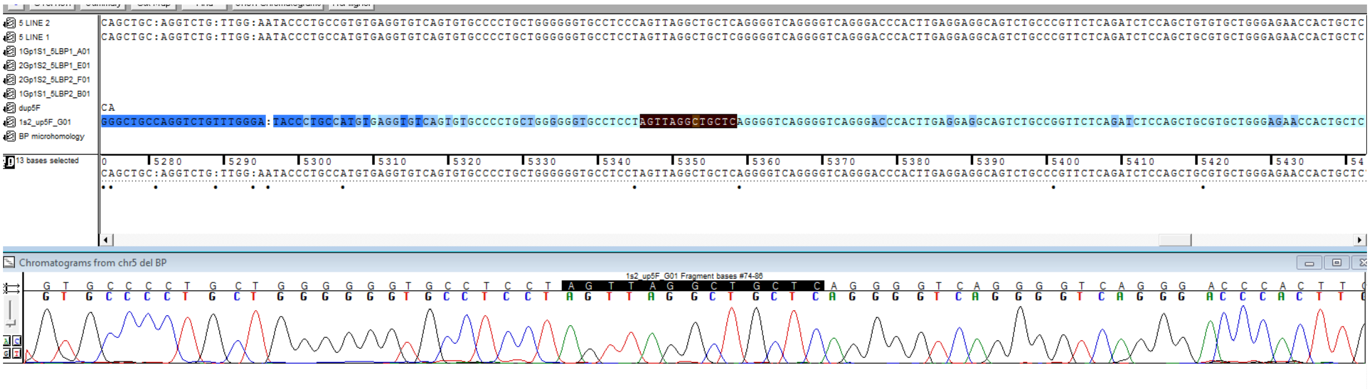

chr9 del patients 1-11

Distal BP: between 72,121,227 and 72,123,902
Proximal BP: between 72,092,280 and 72,094,955

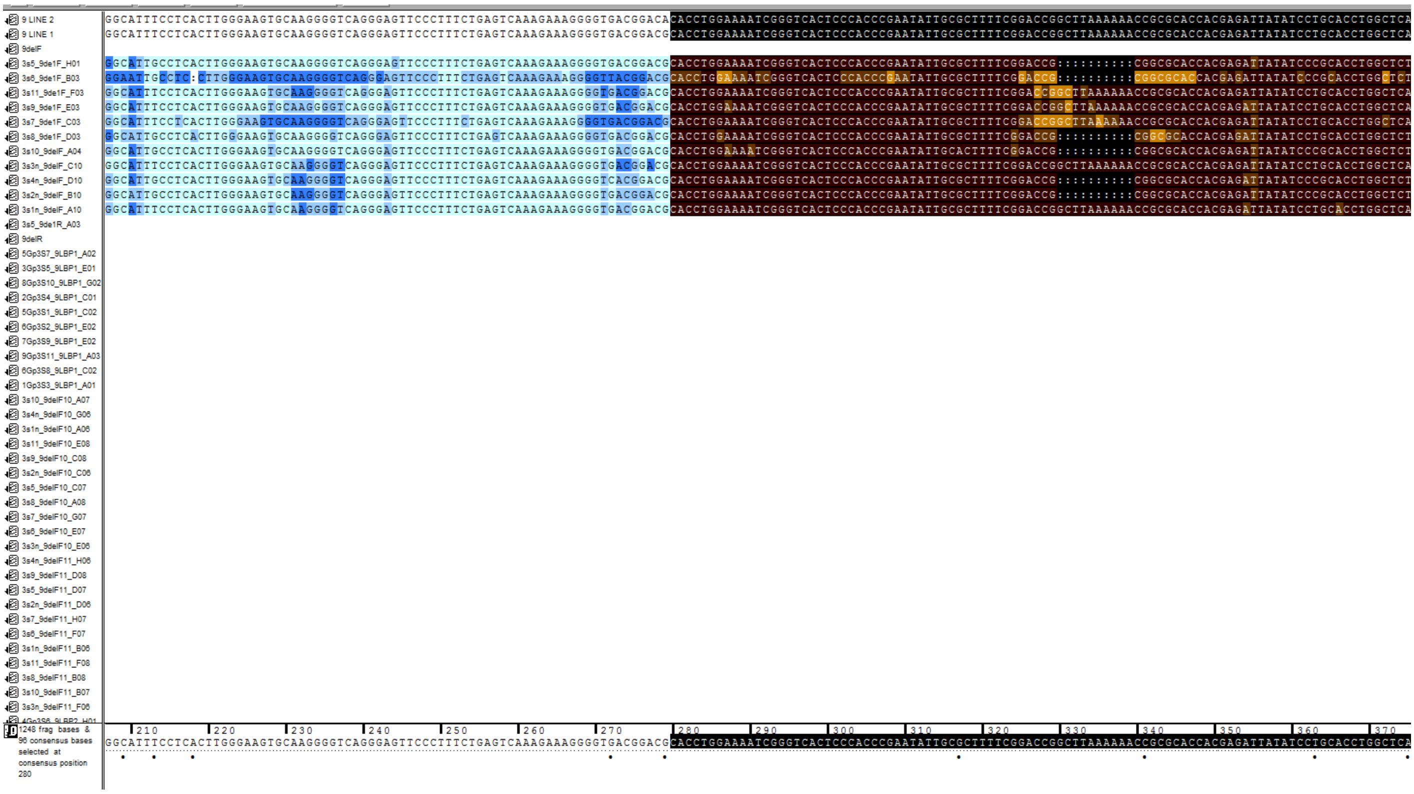

chr9 del patients 1-11

```
>Microhomology:
CACCTGGAAAATCGGGTCACTCCCACCCGAATATTGCGCTTTTCGGACCG
GCTTAAAAAACCGCGCACCACGAGATTATATCCTGCACCTGGCTCAGAGG
GTCCTACGCCCACGGAGTCTCACTGATTGCTAGCACAGCAGTCTGAGATC
AAACTGCAAGGCAGCAGCGAGGCTCGGGGAGGGGCGCCCGCCATTGCCCG
GGCTTGCTTAGGTAAACAAAGCAGCCTGGAAGCTCGAACTGGGTGGAGCC
CACCACAGCTCAAGGAGGCCTGCCTGCCTCTGTAGGCTCCACCTCTGGGG
GCAGGGCACAGACAAACAAAAGGCAGCAGTAGCCTCTGCAGACTTAAATG
TCCCTGTCTGACAGCTTTGAAGAGAGCAGTGGTTCTCCCAGCACGCAGCT
GGAGATCTGAGAACGGGCAGACTGCCTCCTCAAGTGGGTCCCTGACCCCT
GACCCCCGAGCAGCCTAACTGGGAGGCACCCCCCAGCAGGGGCACACTGA
CACCTCACACGGCAGGGTATTCCAACAGACCTGCAGCTGAGGGTCCTGTC
TGTTAGAAGGAAAACTAACAAACAGAAAGGACATCCACACCAAAAACCCA
TCTGTACATTACCATCATCAAAGACCAAAAGTAGATAAAACCACAAAGAT
GGGGAAAAAACAGAACAGAAAAACTGGAAACTCTAAAACGCAGAGCGCCT
CTCCTCCTCCAAAGGAACGCAGTTCCTCACCAGCAACAGAACAAAGCTGG
ATGGAGAATGACTTTGACGAGCTGAGAGAAGAAGGCTTCAGACGATCAAA
TTACTCTGAGCTACGGGAGGACATTCAAACCAAAGGCAAGGAAGTTGAAA
ACTTTGAAAAAAATTTAGAAGAATGTATAACTAGAATAACCAATACAGAG
AAGTGCTTAAAGGAGCTGATGGAGCTGAAAACCAAGGCTCGAGAACTACG
TGAAGAATGCAGAAGCCTCAGGAGCCGATGCGATCAACTGGAAGAAAGGG
TATCAGCGATGGAAGATGAAATGAATGAAATGAAGCGAGAAGGGAAGTTT
AGAGAAAAAAAGAATAAAAAGAAATGAGCAAAGCCTCCAAGAAGTATGGG
ACTATGTGAAAAGACCAAATCTACGTCTGATTGGTGTACCTGAAAGTGAT
GGGGAGAATGGAACCAAGTTGGAAAACACTCTGCAGGATATTATCCAGGA
GAACTTCCCCAATCTAGCAAGGCAGGCCAACGTTCAGATTCAGGAAATAC
AGAGAACGCCACAAAGATACTCCTTGAGAAGAGCAACTCCAAGACACATA
ATTGTCAGATTCACCAAGGTTGAAATGAAGGAAAAAATGTTAAGGGCAGC
CAGAGAGAAAGGTCGGGTTACCCTCAAAGGGAAGCCCATCAGACTAACAG
TGGATCTCTCAGCAGAAACCCTACAAGCCAGAAGAGAGTGGGGGCCAATA
TTCAACATTCTTAAAGAAAAGAATTTTCAACCCAGAATTTCATATCCAGC
CAAACTAAGCTTCATAAGTGAAGGAGAAATAAAATACTTTACAGACAAGC
AAATGCTGAGAGATTTTGTCACCACCAGGCCTGCCCTAAAAGAGCTCCTG
AAGGAAGCGCTAAACATGGAAAGGAACAACCGGTACCAGCCGCTGCAAAA
TCATGCCAAAATGTAAAGACCATCGAGGCTAGGAAGAAACTGCATCAACT
AACGAGCAAAATCACCAGCTAACATCATAATGACAGGATCAAATTCACAC
ATAACAATATTAACTTTAAATGTAAATGGACCAAATGCTCCAATTAAAAG
ACACAGACTGGCAAATTGGATAAAGAGTCAAGACCCATCAGTGTGCTGTA
TTCAGGAAACCCATCTCACGTGCAGAGACACACATAGGCTCAAAATAAAA
GGATGGAGGAAGATCTACCAGGCAAATGGAAAACAAAAAAAGGCAGGGGT
TGCAATCCTAGTCTCTGATAAAACAGACTTTAAACCAACAAAGATCAAAA
GAGACAAAGAAGGCCATTACATAATGGTAAAGGGATCAATTCAACAAGAA
GAGCTAACTATCCTAAATATATATGCACCCAATACAGGAGCACCCAGATT
CATAAAGCAAGTCCTGAGTGACCTACAAAGAGACTTAGACTCCCACACTT
TAATAATGGGAGACTTTAACACCCCACTGTCAACATTAGACAGATCAACG
AGACAGAAAGTCAACAAGGATACCCAGGAATTGAACTCAGCTCTGCACCA
GGTGGACCTAATTGACATCTACAGAACTCTCCACCCCAAATCAACAGAAT
ATACATTTTTTTCAGCACCACACCACAGCTATTCCAAAATTGACCACATA
CTTGGAAGTAAAGCTCTCCTCAGCAAATGTAAAAGAACAGACATTATAAC
AAACTATCTCTCAGACCACAGTGCTATCAAACTAGAACTCAGGATTAAGA
ATCTCACTCAAAACCGCTCAACTACATGGAAACTGAACAACCTGCTCCTG
AATGACTACTGGATACATAACGAAATGAAGGCAGAAATAAAGATGTTCTT
TGAAACCAACGAGAACAAAGACACAACATACCAGAATCTCTGGGACGCAC
TCAAAGCAGTGTGTAGAGGGAAATTTATAGCACTAAATGCCCACAAGAGA
AAGCAGGAAAGATCCAAAATTGAC
```

chr9 del patients 1-11

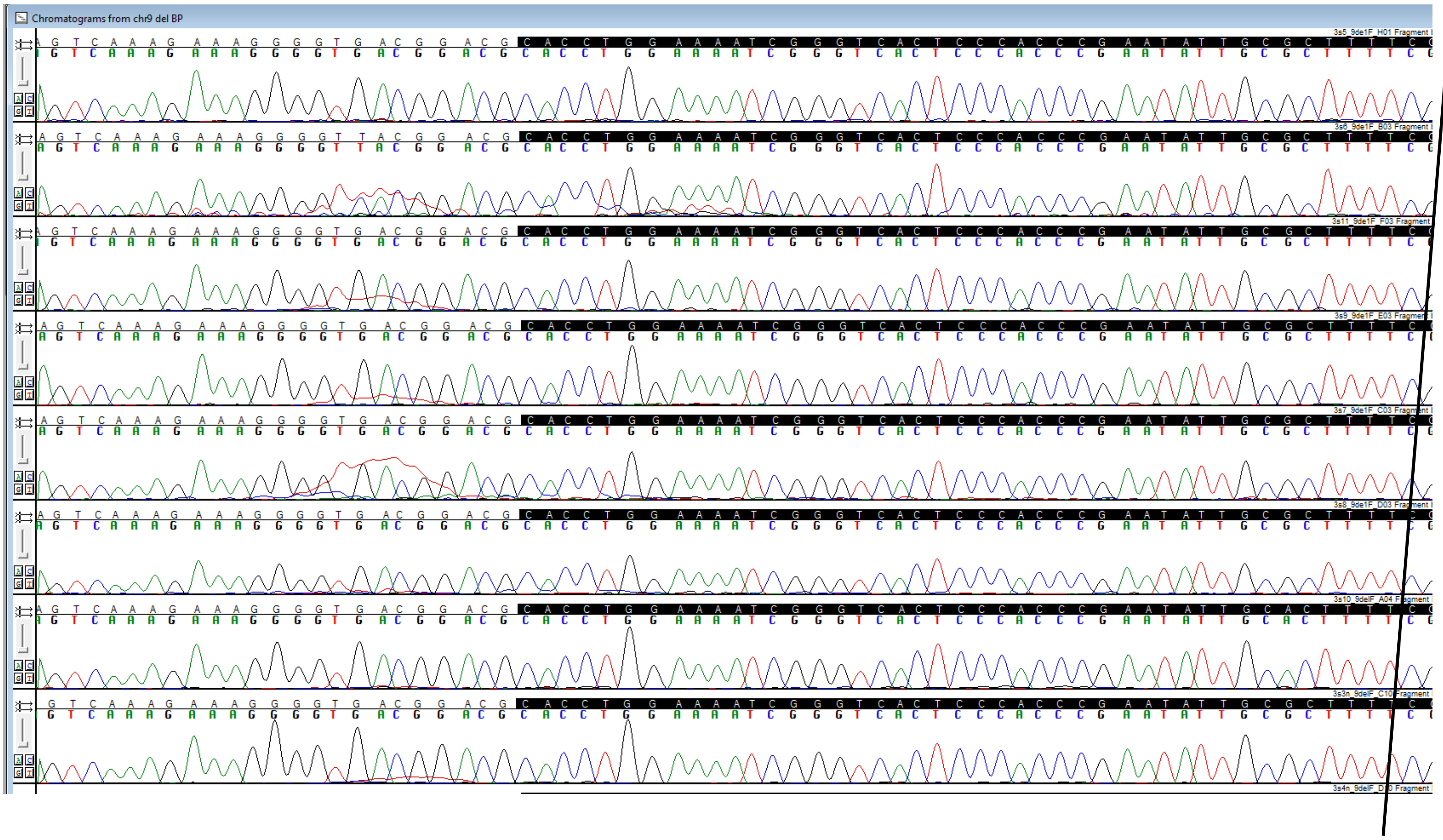

chr9 del patients 1-11

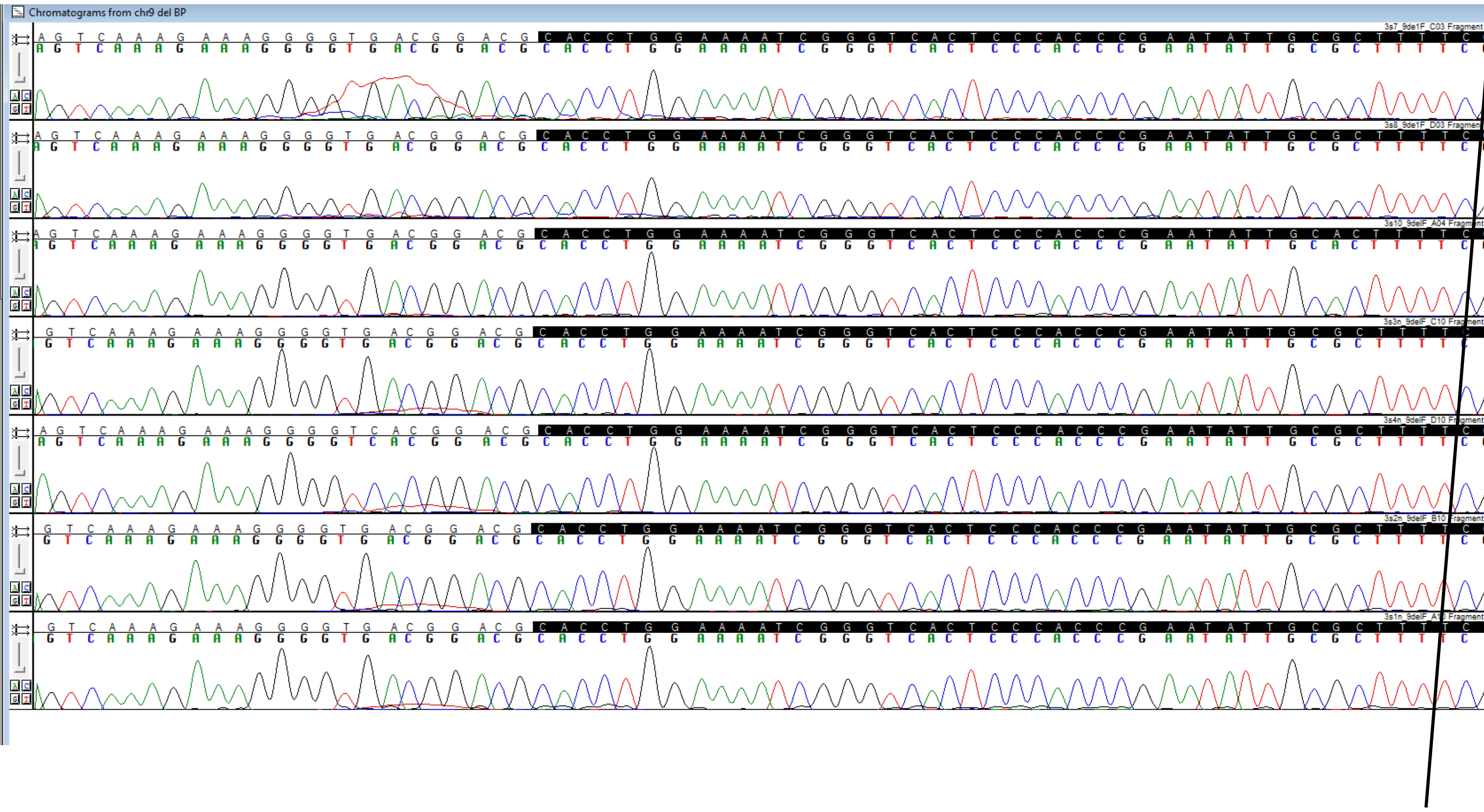

chr9 del patients 1-11

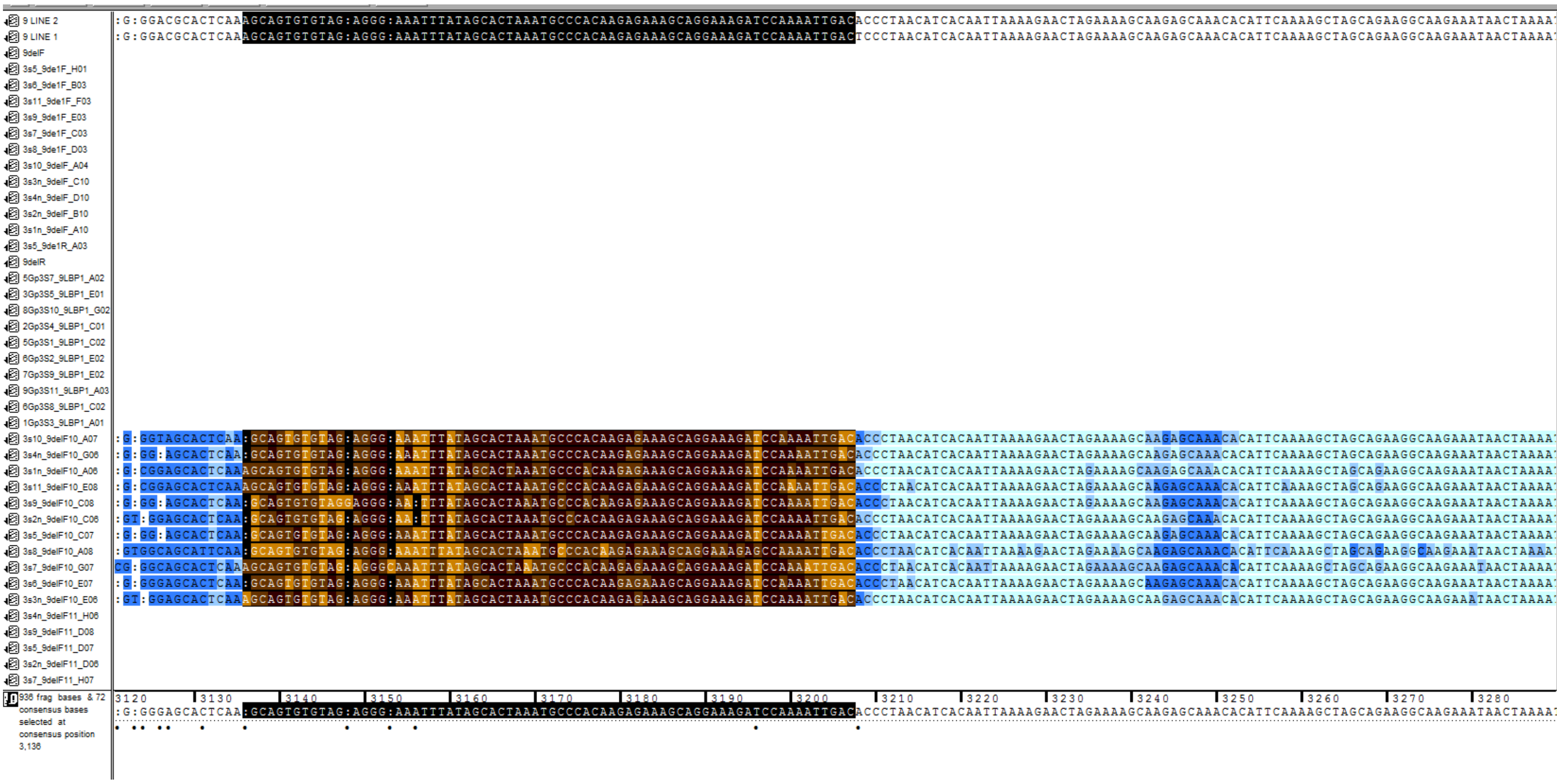

chr9 del patients 1-11

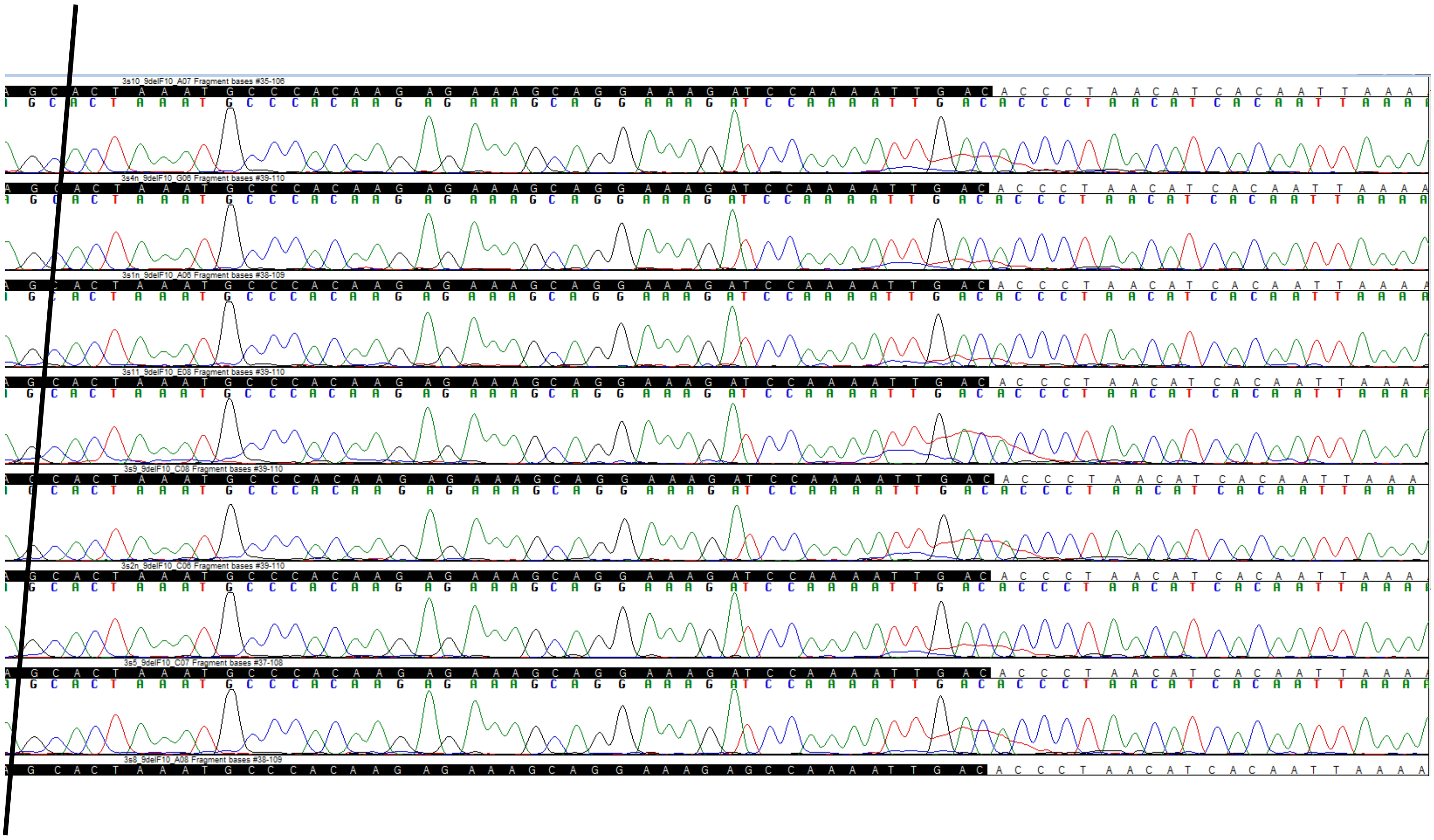

chr9 del patients 1-11

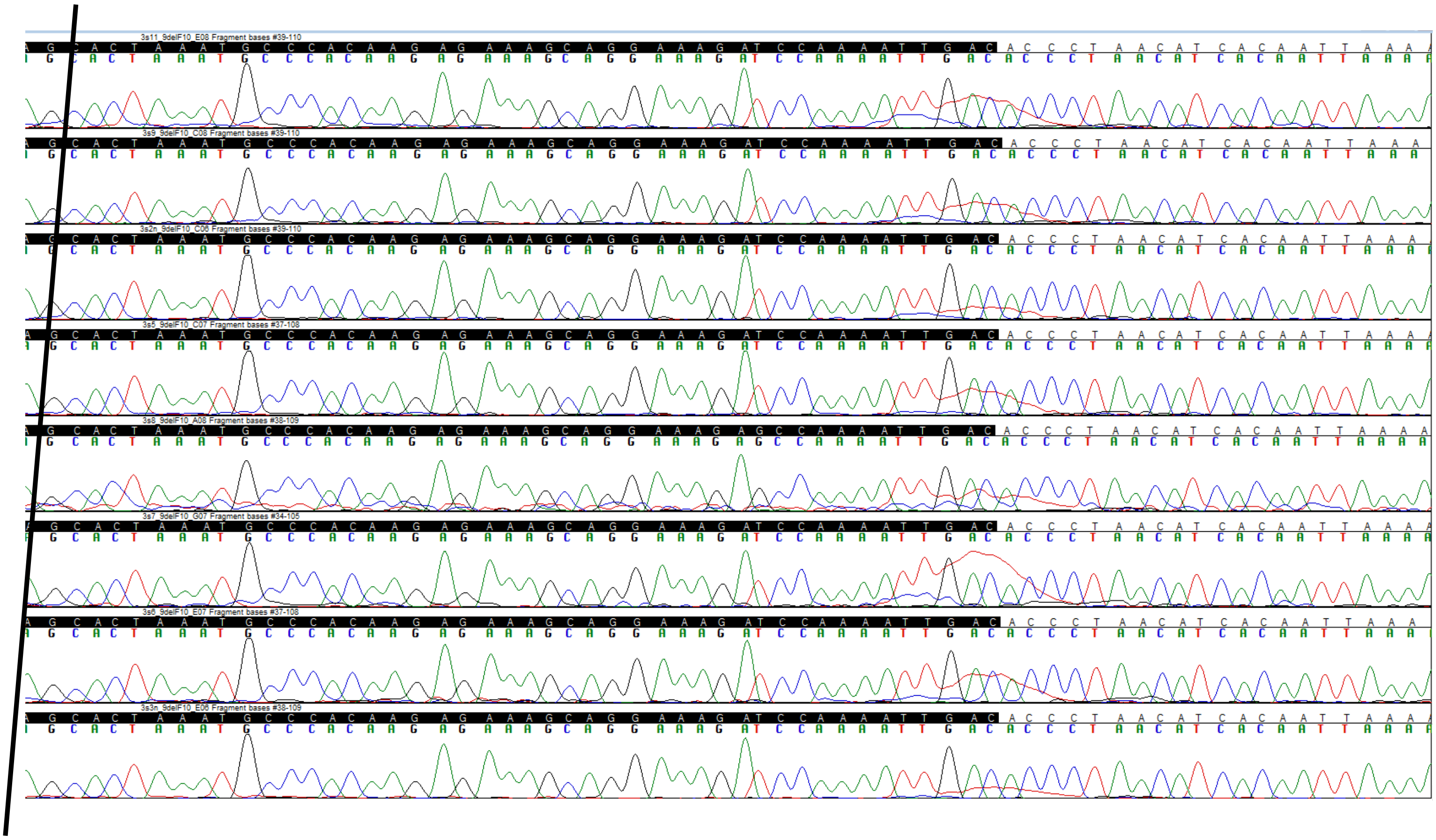

chr11 del patient 1

DistalBP: between 26,987,712 and 26,987,779
Proximal BP: between 27,242,299 and 27,242,366
Microhomology: GATCAAATTACTCTGAGCTACGGGAGGACATTCAAACCAAAGGCAAAGAAGTTGAAAACTTTGAAA

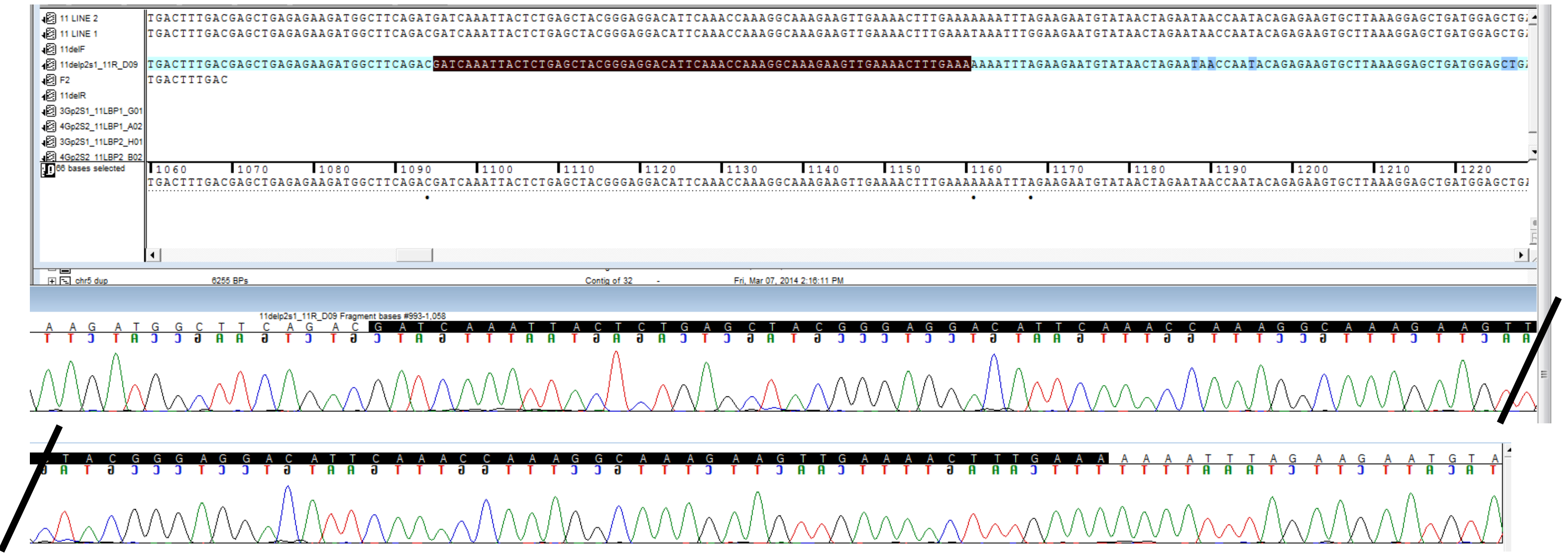

chr11 del patients 1 and 2

DistalBP: between 26,987,712 and 26,987,779
Proximal BP: between 27,242,299 and 27,242,366
Microhomology: GATCAAATTACTCTGAGCTACGGGAGGACATTCAAACCAAAGGCAAAGAAGTTGAAAACTTTGAAA

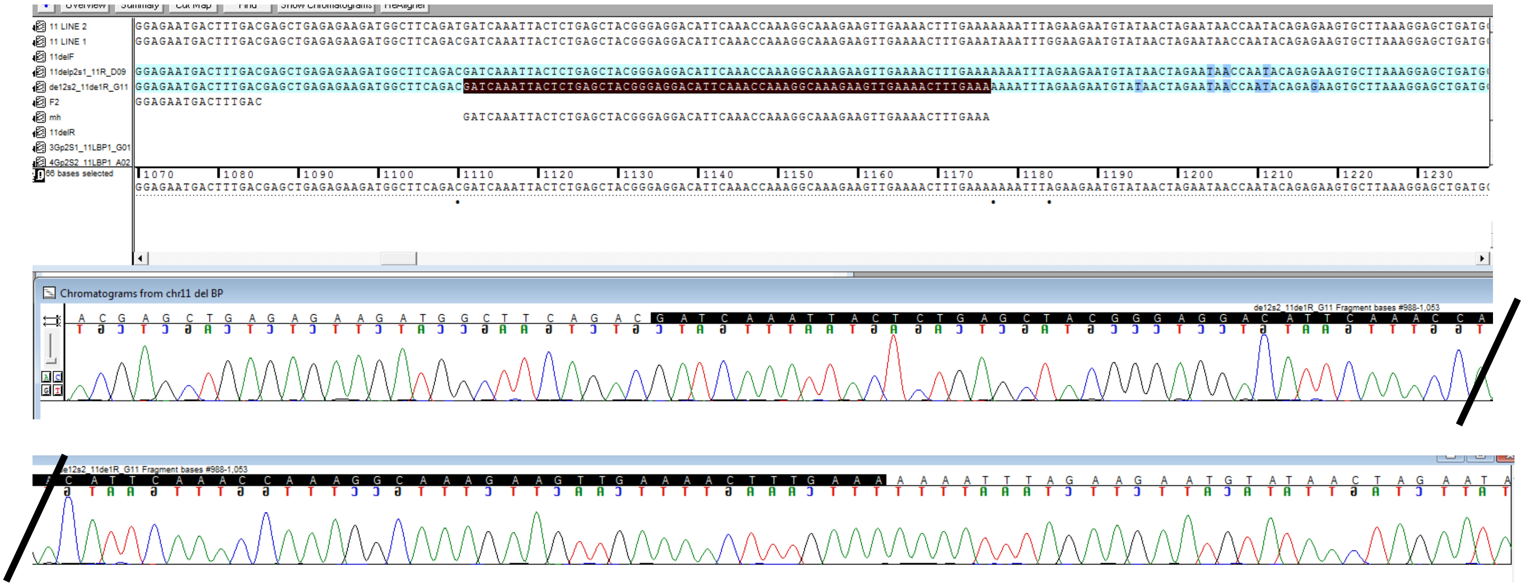

chr12 del patient 2

Distal BP: between 5,230,073 and 5,230,256
Proximal BP: between 5,398,600 and 5,398,783

Microhomology:
CTCGAGGAGTATCTTTGTGGCGTTCTCTGTATTTCCTGAATCTGAACGTTGGCCTGCCTTGCTAGATTGGGGAAGTTCTCCTGGATAATA
TCCTGCAGAGTGTTTTCCAACTTGGTTCCATTCTCCCCATCACTTTCAGGTACACCAATCAGACGTAGATTTGGTCTTTTCACATAGTCC
CA

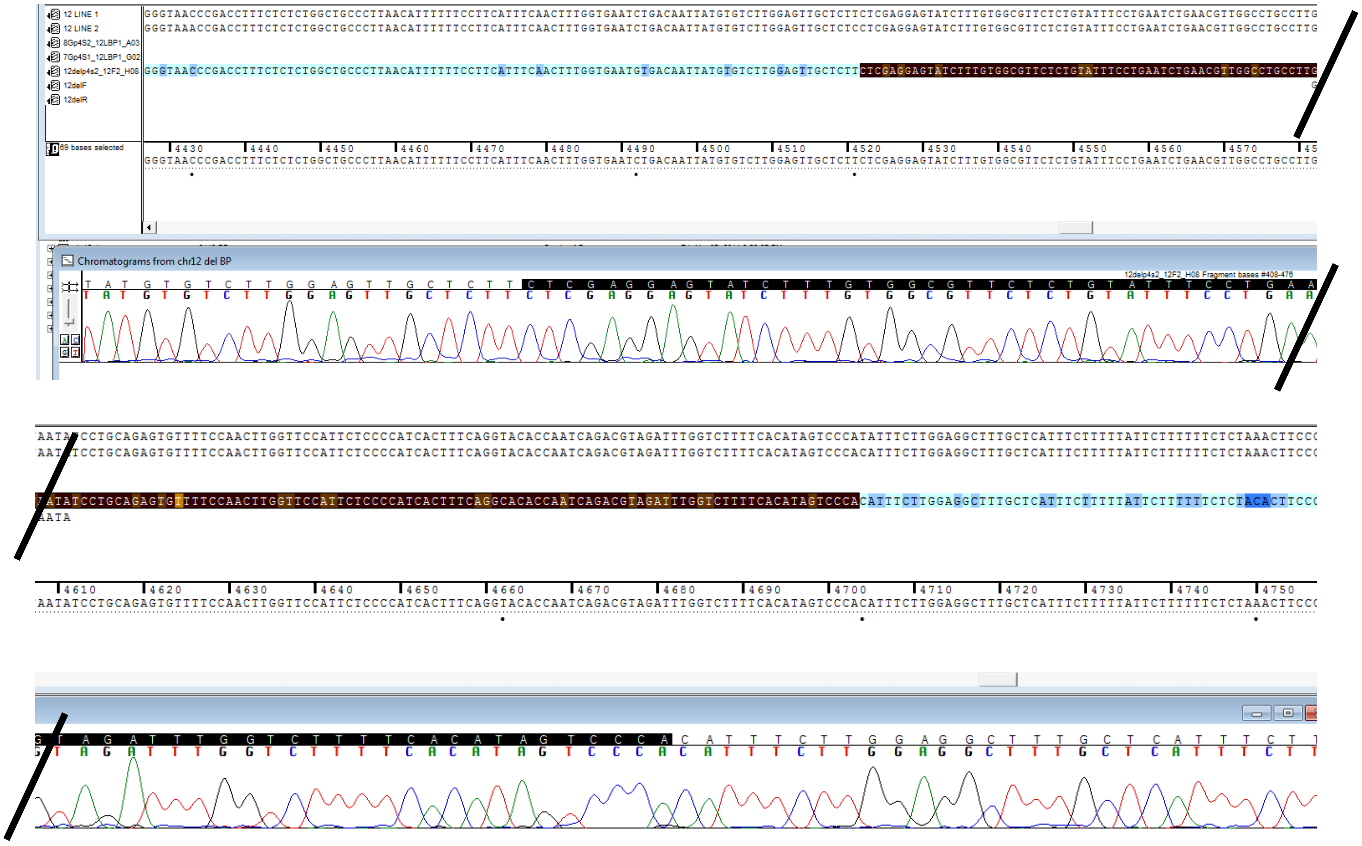

chr12 del patient 4

Distal BP: between 5,230,073 and 5,230,256
Proximal BP: between 5,398,600 and 5,398,783

Microhomology:
CTCGAGGAGTATCTTTGTGGCGTTCTCTGTATTTCCTGAATCTGAACGTTGGCCTGCCTTGCTAGATTGGGGAAGTTCTCCTGGATAATA
TCCTGCAGAGTGTTTTCCAACTTGGTTCCATTCTCCCCATCACTTTCAGGTACACCAATCAGACGTAGATTTGGTCTTTTCACATAGTCC
CA

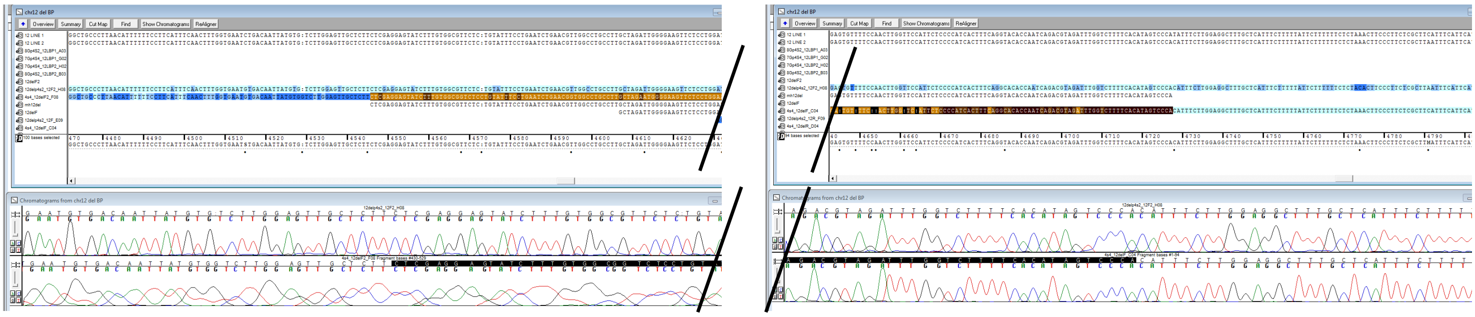

chr20 del patients 1 & 4

Distal BP: between 8,094,804 and 8,094,894
Proximal BP: between 8,581,012 and 8,581102
Microhomology:
GTGTCAGTGTGCCCCTGCTGGGGGGTGCCTCCCAGTTAGGCTGCTCGGGGGTCAGGGGTCAGGGACCCACTTGAGGAGGCAGTCTGCC
C

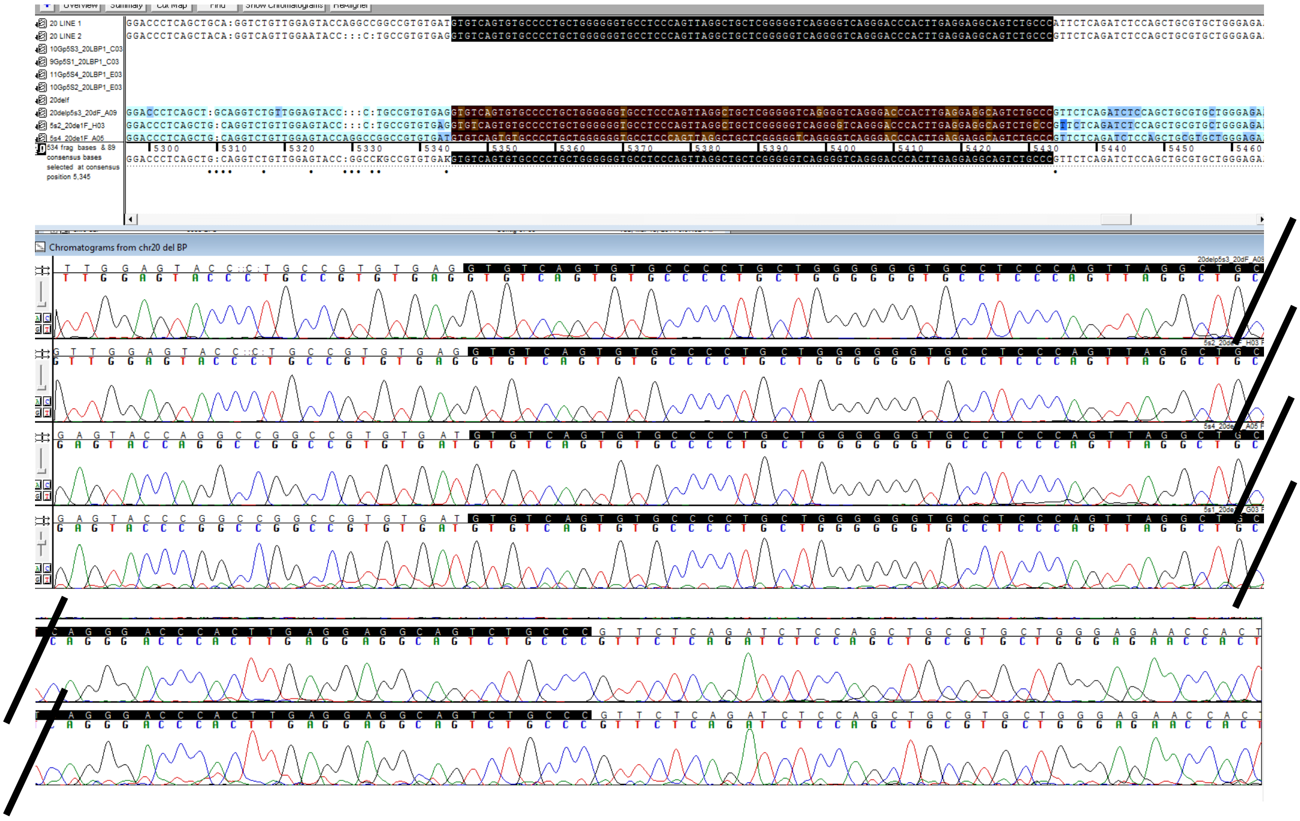

chr20 del patients 2 & 3

Distal BP: between 8,094,566 and 8,094,629
Proximal BP: between 8,580,778 and 8,580,841
Microhomology: GTTGCTGGTGAGGAACTGCGTTCCTTTGGAGGAGGAGAGGCGCTCTGCGTTTTAGAGTTTCCC

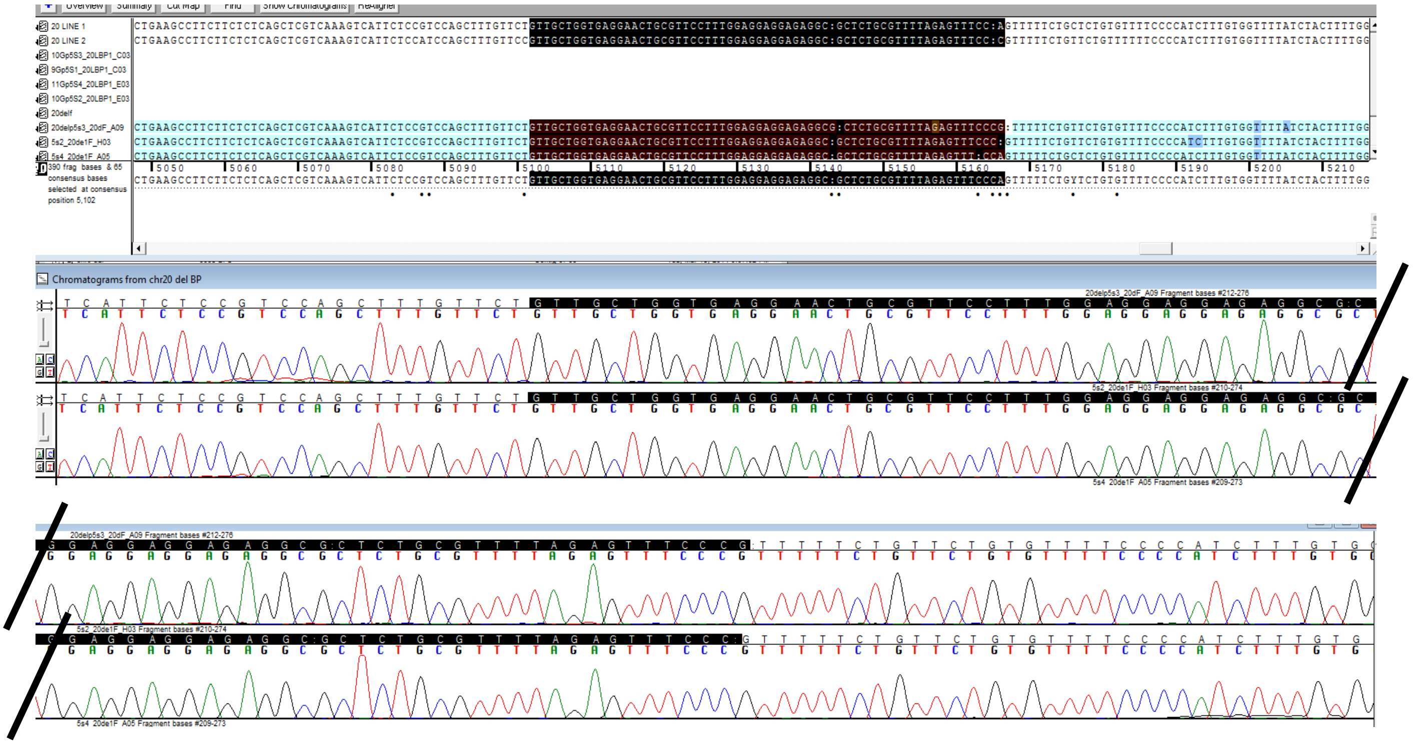

Duplications

chr5 del patient 1

proximal BP: between 25,384,363 and 25,384,397
distal BP: between 25,083,490 and 25,083,524
microhomology: TCAGAAAAGCGCAGTATTCGGGTGGGAGTGACC

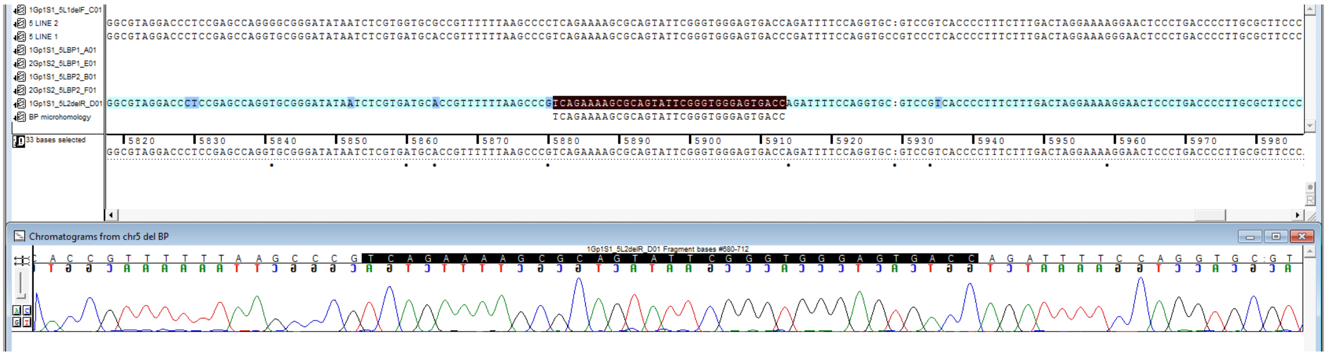

chr5 dup patients 2-15

Distal BP: between
Proximal BP: between
Microhomology: GGGGTCAGGGGTCAGGGACCCACTTGAGGAGGCAGTCTGCCCGTTCTCAGATCTCCAGCTG

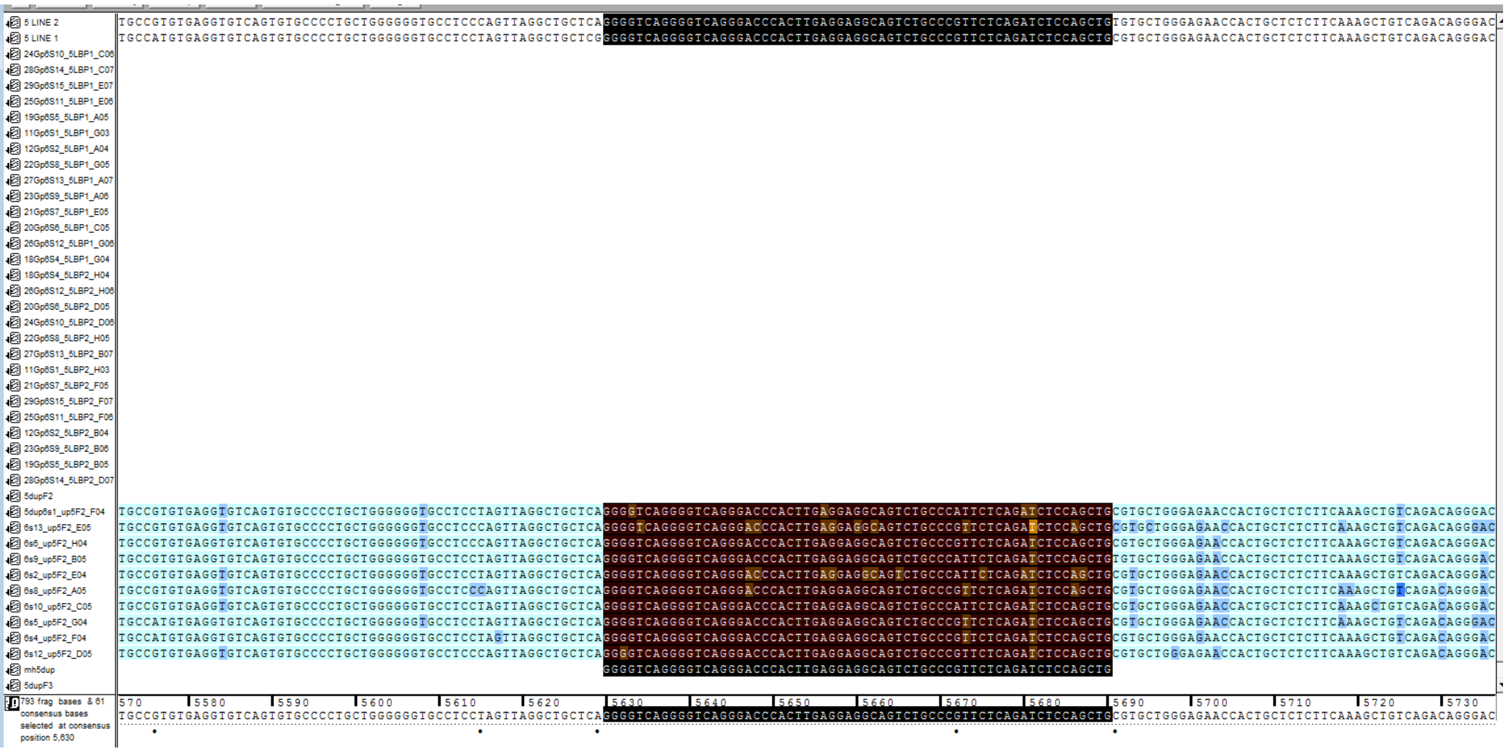

chr5 dup patients 2-15(for 14, see also next slide)

For all except 9
Distal BP: between 25,083,080 and 25,083,142
Proximal BP: between 25,383,953 and 25,384,015
Microhomology: GGGGTCAGGGGTCAGGGACCCACTTGAGGAGGCAGTCTGCCCGTTCTCAGATCTCCAGCTG

For 9
Distal BP: between 25,083,142-25,083,283
Proximal BP: between 25,384,015-25,384,156
Microhomology:GTGCTGGGAGAACCACTGCTCTCTTCAAAGCTGTCAGACAGGGACATTTAAGTCTGCAGAGGTTACTGC
TGTCTTTTTGTTTGTCTGTGCCCTGCCCCCAGAGGGGGAGCCTACAGAGGCAGGCAGGCCTCATTGAGCTG

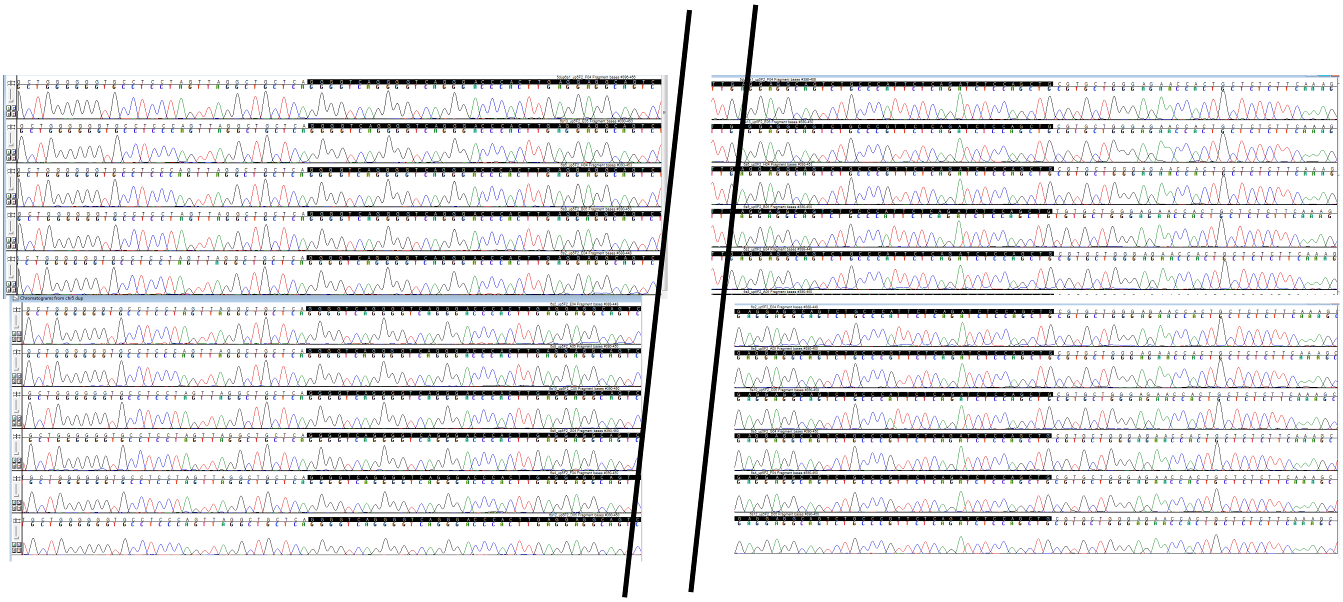

chr5 dup patient 14

Distal BP: between 25,083,080 and 25,083,142
Proximal BP: between 25,383,953 and 25,384,015
Microhomology: GGGGTCAGGGGTCAGGGACCCACTTGAGGAGGCAGTCTGCCCGTTCTCAGATCTCCAGCTG

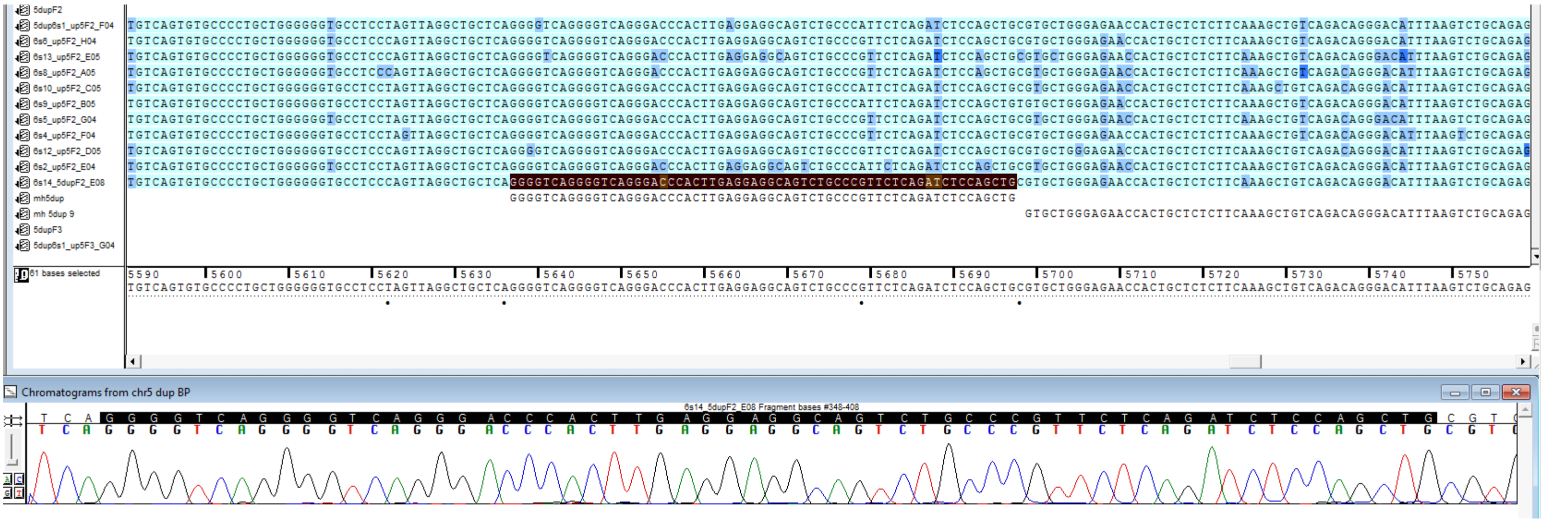

chr9 dup patient 1

Distal BP: between 72,124,084 and 72,124,633
Proximal BP: between 72,095,137 and 72,095,686
>Microhomology:
CTAGCAAGACTAATAAAGAAAAAAAGAGAGAGGAATCAAATAGACACAATAAAAAATGATAAAGGGGATATCACCACCGATCCCACAGAAATACAAACTACCATC
AGAGAATACTACAAACACCTCTACGCAAATAAACTAGAAAATCTAGAAGAAATGGATAAATTCCTCGACACATACACTCTCCCAAGACTAAACCAGGAAGAAGTT
GAATCTCTGAATAGACCAATAACGGGAGCTGAAATTGTGGCAATAATCAATAGTTTACCAACCAAAAAGAGTCCAGGACCAGATGGATTCACAGCCAAATTCTAC
CAGAGGTACAAGGAGGAACTGGTACCATTCCTTCTGAAACTATTCCAATCAAGAGAAAAAGAGGGAATCCTCCCTAACTCATTTTATGAGGCCAGCATCATTCTG
ATACCAAAGCCGGGCAGAGACACAACCAAAAAAGAGAATTTTAGACCAATATCCTTGATGAACATTGATGCAAAAATCCTCAATAAAATACTGGCAAAACGAATC
CAGCAGCACATCAAAAAGCTTAT

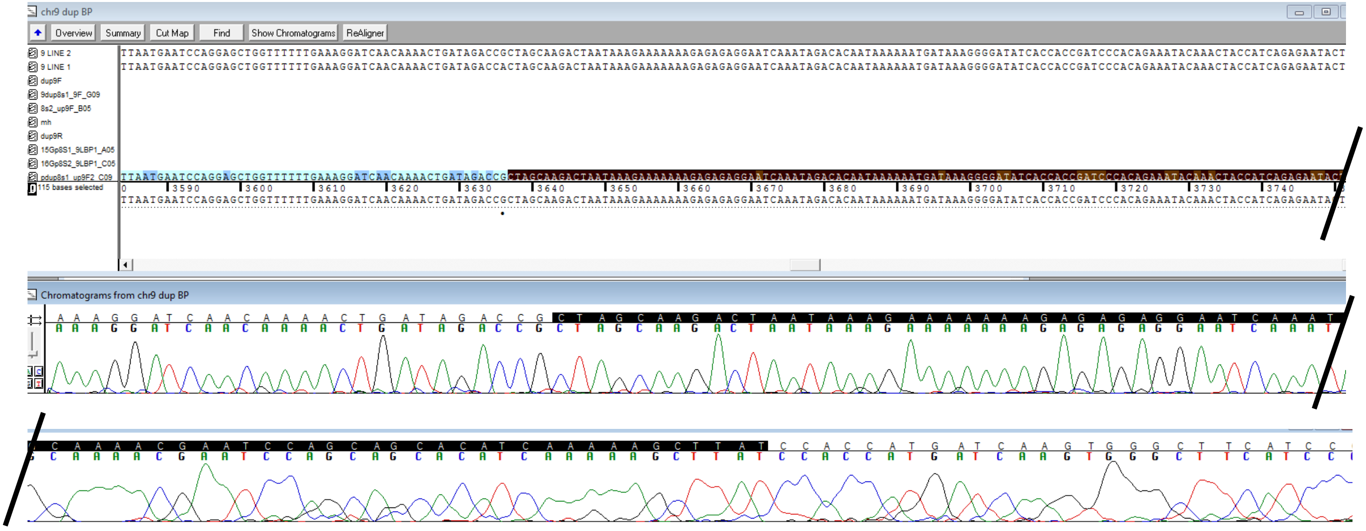

chr9 dup patient 2

Distal BP: between 72,124,084 and 72,124,633
Proximal BP: between 72,095,137 and 72,095,686
>Microhomology:
CTAGCAAGACTAATAAAGAAAAAAAGAGAGAGGAATCAAATAGACACAATAAAAAATGATAAAGGGGATATCACCACCGATCCCACAGAAATACAAACTACCATC
AGAGAATACTACAAACACCTCTACGCAAATAAACTAGAAAATCTAGAAGAAATGGATAAATTCCTCGACACATACACTCTCCCAAGACTAAACCAGGAAGAAGTT
GAATCTCTGAATAGACCAATAACGGGAGCTGAAATTGTGGCAATAATCAATAGTTTACCAACCAAAAAGAGTCCAGGACCAGATGGATTCACAGCCAAATTCTAC
CAGAGGTACAAGGAGGAACTGGTACCATTCCTTCTGAAACTATTCCAATCAAGAGAAAAAGAGGGAATCCTCCCTAACTCATTTTATGAGGCCAGCATCATTCTG
ATACCAAAGCCGGGCAGAGACACAACCAAAAAAGAGAATTTTAGACCAATATCCTTGATGAACATTGATGCAAAAATCCTCAATAAAATACTGGCAAAACGAATC
CAGCAGCACATCAAAAAGCTTAT

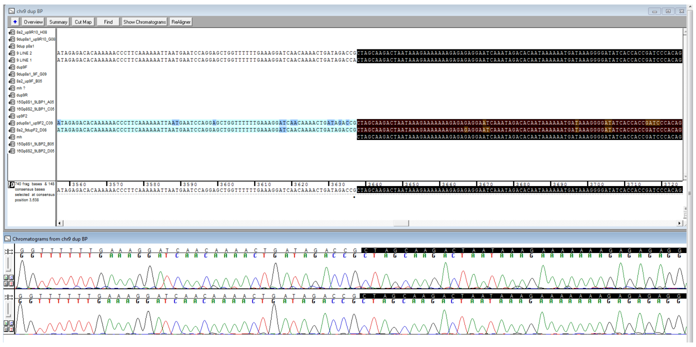

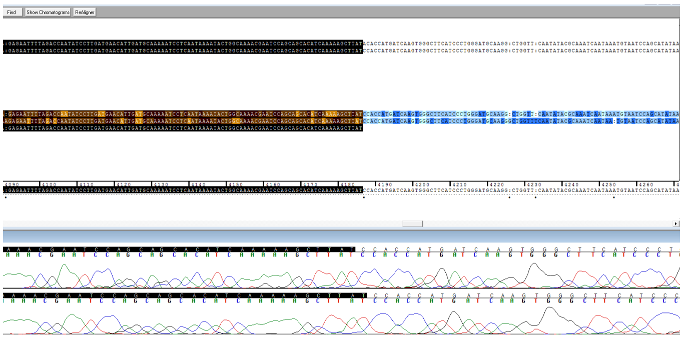

chr11 dup patient 1

proximal BP: between 27,241,553 and 27,241,763
distal BP: between 26,987,002 and 26,987,176
microhomology:
GCACCTGGCTCGGAGGGTCCTACGCCCACGGAGTCTCGCTGATTGCTAGCACAGCAGTCTGAGATCAAACTGCAAGGCGGCAGCGAGGCTGGG
GGAGGGGCGCCCGCCATTGCCCAGGCTTGATTAGGTAAACAAAGCAGCGGGGAAGCTCGAACTGGGTGGAGCCCACCACA

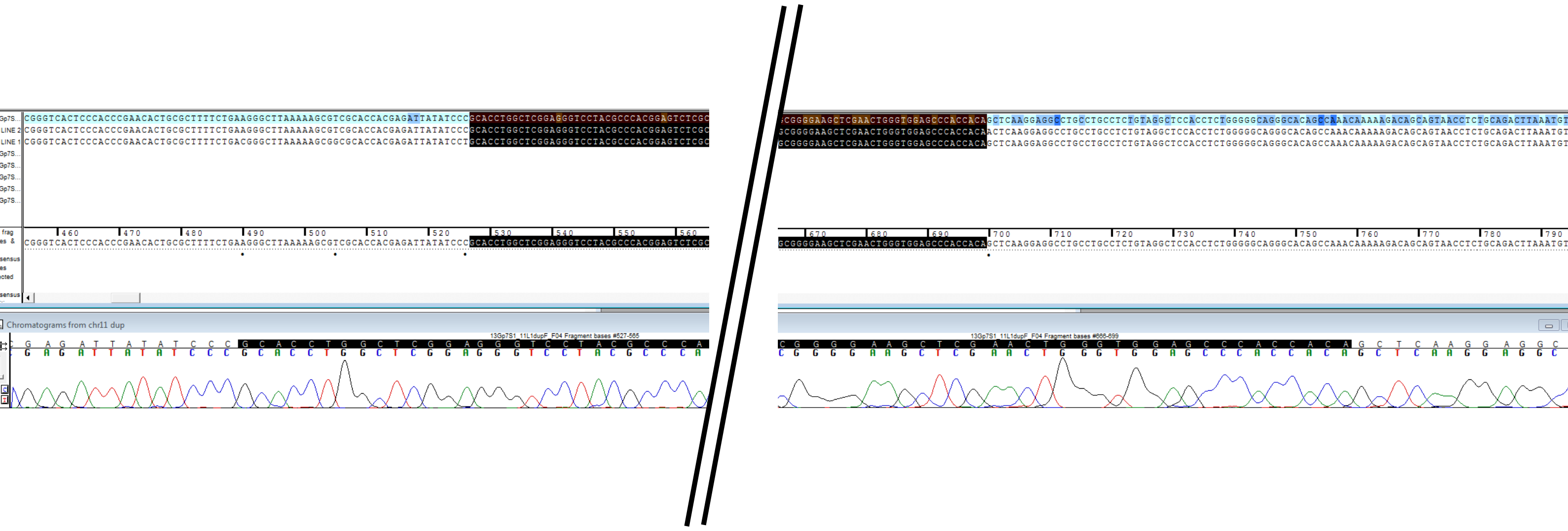

chr11 dup patient 2

Distal BP: between 27,241,366 and 27,241,553
Proximal BP: between 26,986,779 and 26,986,966
microhomology:
GGAGTGCCAGACAGTGGGCGCAGGTCAGCGGGTGCGCGCACCGTGCGCGAGCCGAAGCAGGGCGAGGCATTGCCTCACTTGGGAAGCGCAAG
GGGTCAGGGAGTTCCCTTTCTGAGTCAAAGAAAGGGGTGACGGGCGGCACCTGGAAAATCGGGTCACTCCCACCCGAACACTGCGCTTTTCT
GA

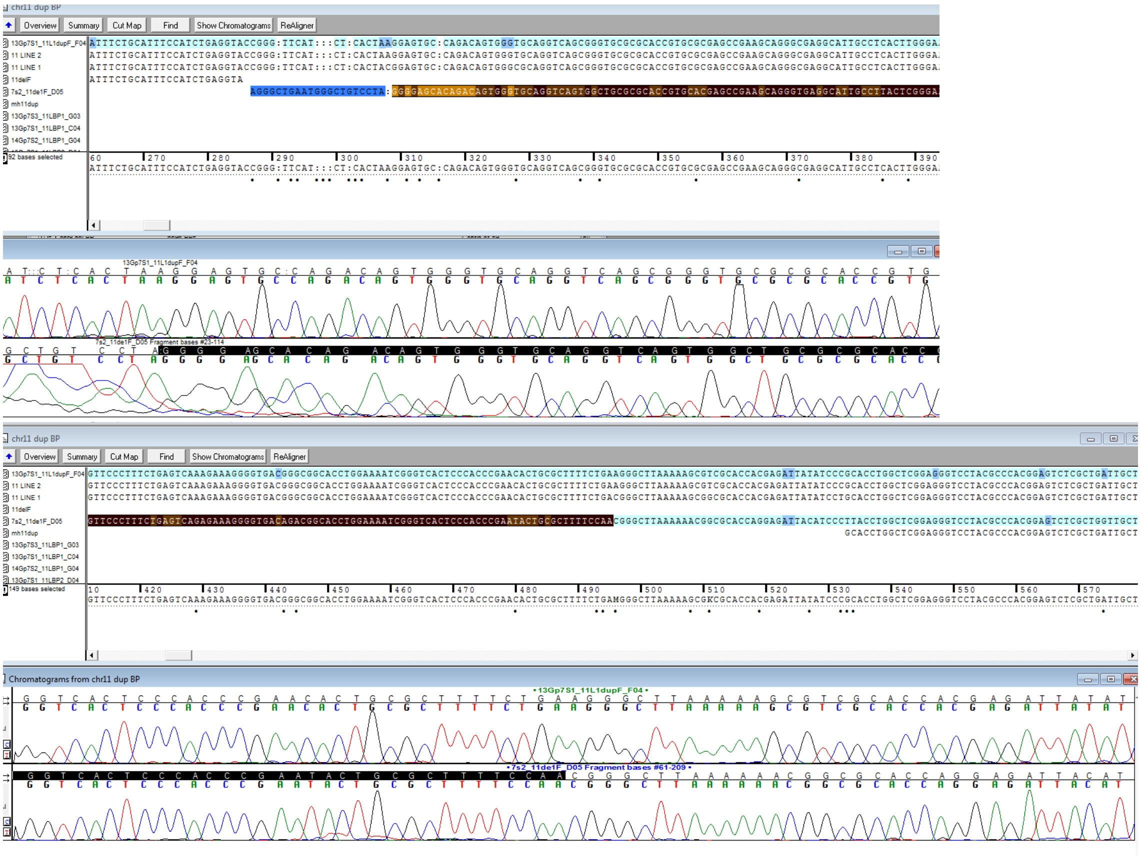

chr11 dup patients 3 & 4

proximal BP: between 27,241,366 and 27,241,385
distal BP: between 26,986,779 and 26,986,798
microhomology: GGAGTGCCAGACAGTGGG

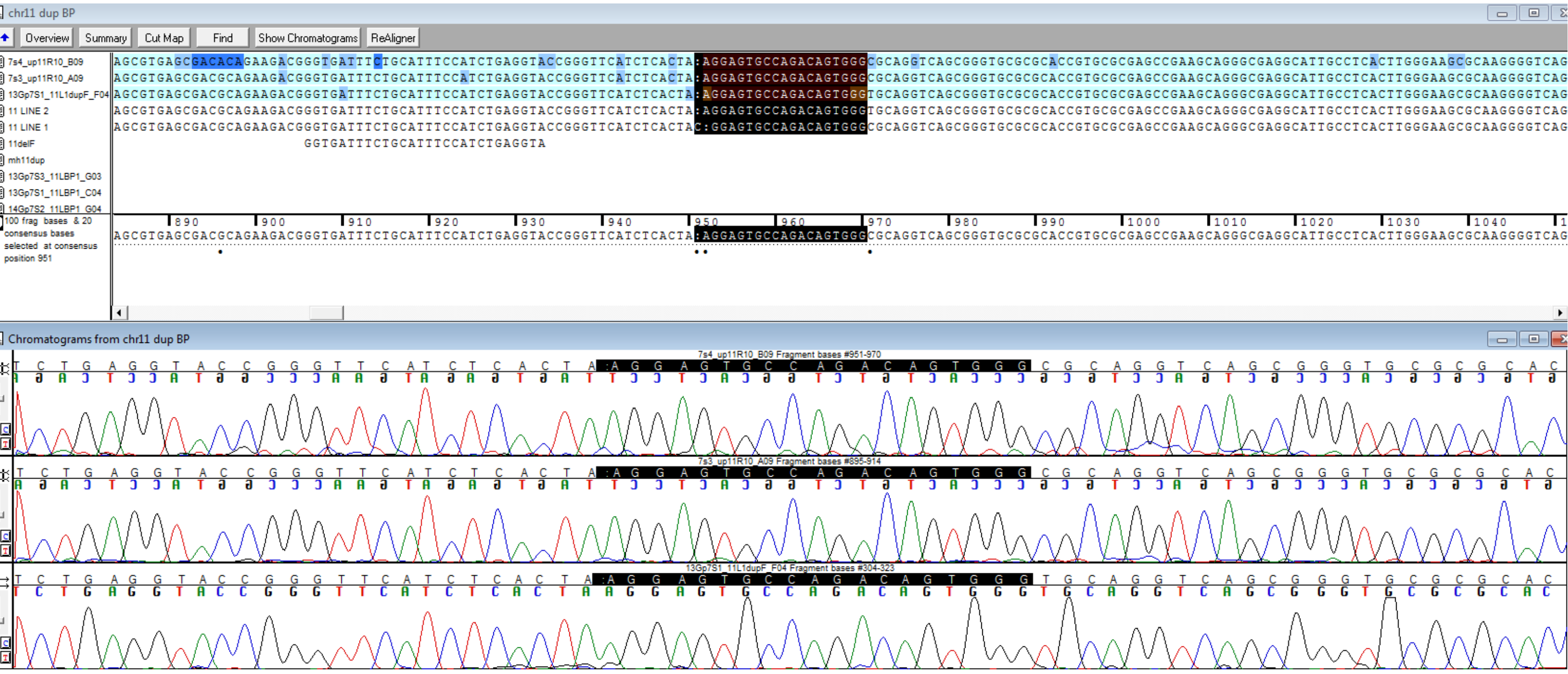

chr12 dup patient 2

Distal BP: between 5,230,858 and 5,231,021
Proximal BP: between 5,399,385 and 5,399,548
Microhomology:
CCCCTGCTGGGGGGTGCCTCCCAGTTAGGCTGCTCGGGGGTCAGGGGTCAGGGACCCACTTGAGGAGGCAGTCTGCCCGTT
CTCAGATCTCCAGCTGCGTGCTGGGAGAACCAGTGCTCTCTTCAAAGCTGTCAGACAGGGACATTTAAGTCTGCAGAGGTT

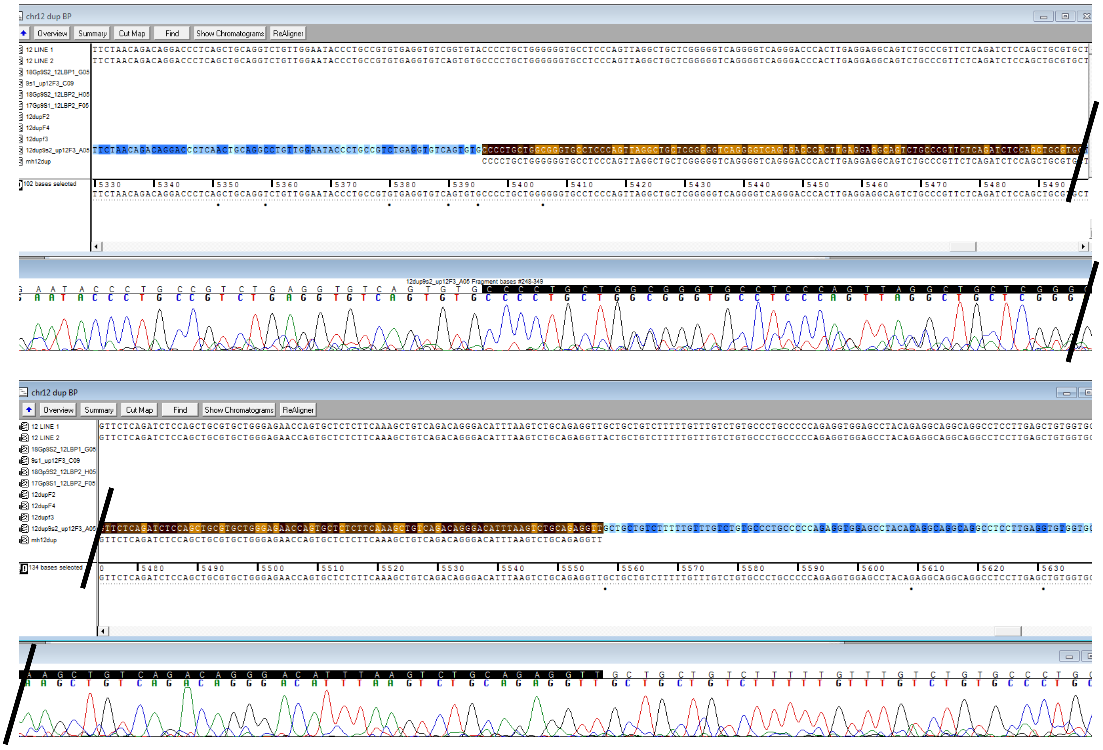

chr20 dup patient 1

Distal BP: 8,095,064-8,095,065
Proximal BP: 8,581,272-8,581,273

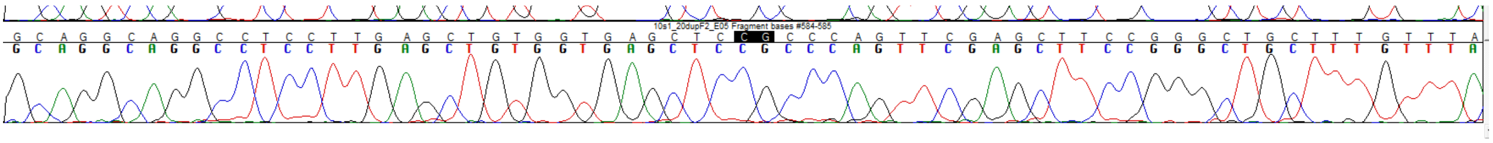

chr20 dup patient 2

```
Distal BP: between 8,094,511 and 8,094,552
Proximal BP: between 8,580,723 and 8,580,764
Microhomology: TCTGAAGCTTCTTCTCTCAGCTCGTCAAAGTCATTCTCC
```

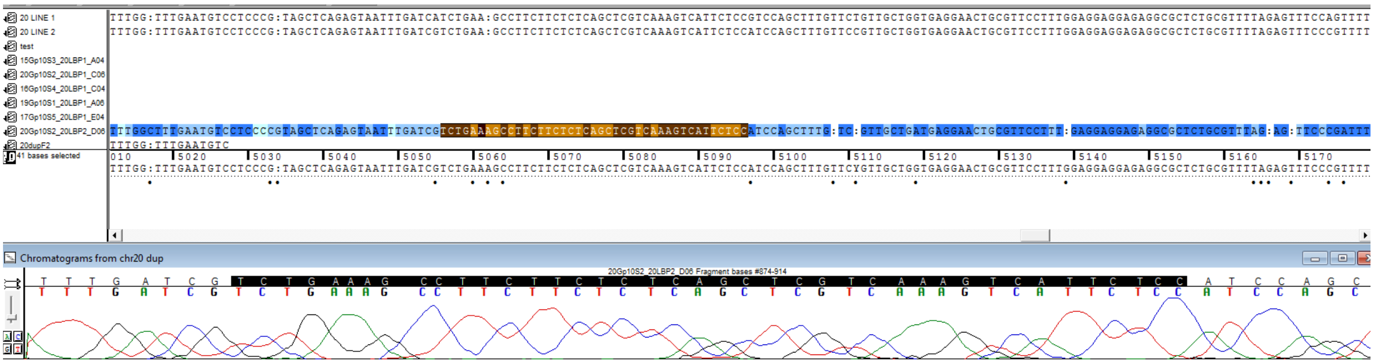

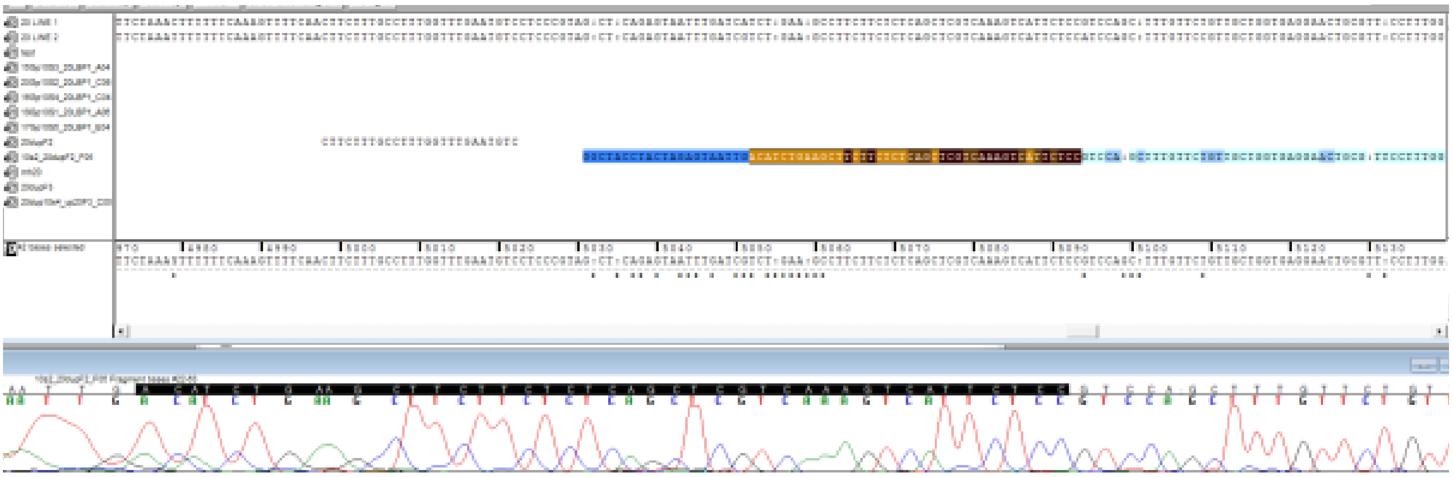

chr20 dup patients 3,4,5

Distal BP: between 8,094,645 and 8,094,725
Proximal BP: between 8,580,857 and 8,580,937
Microhomology: TTTTCCCCATCTTTGTGGTTTTATCTACTTTTGGTCTTTGATGATGG:TGATGTACAGATGGGTTTTTGGTGTGGATGTC

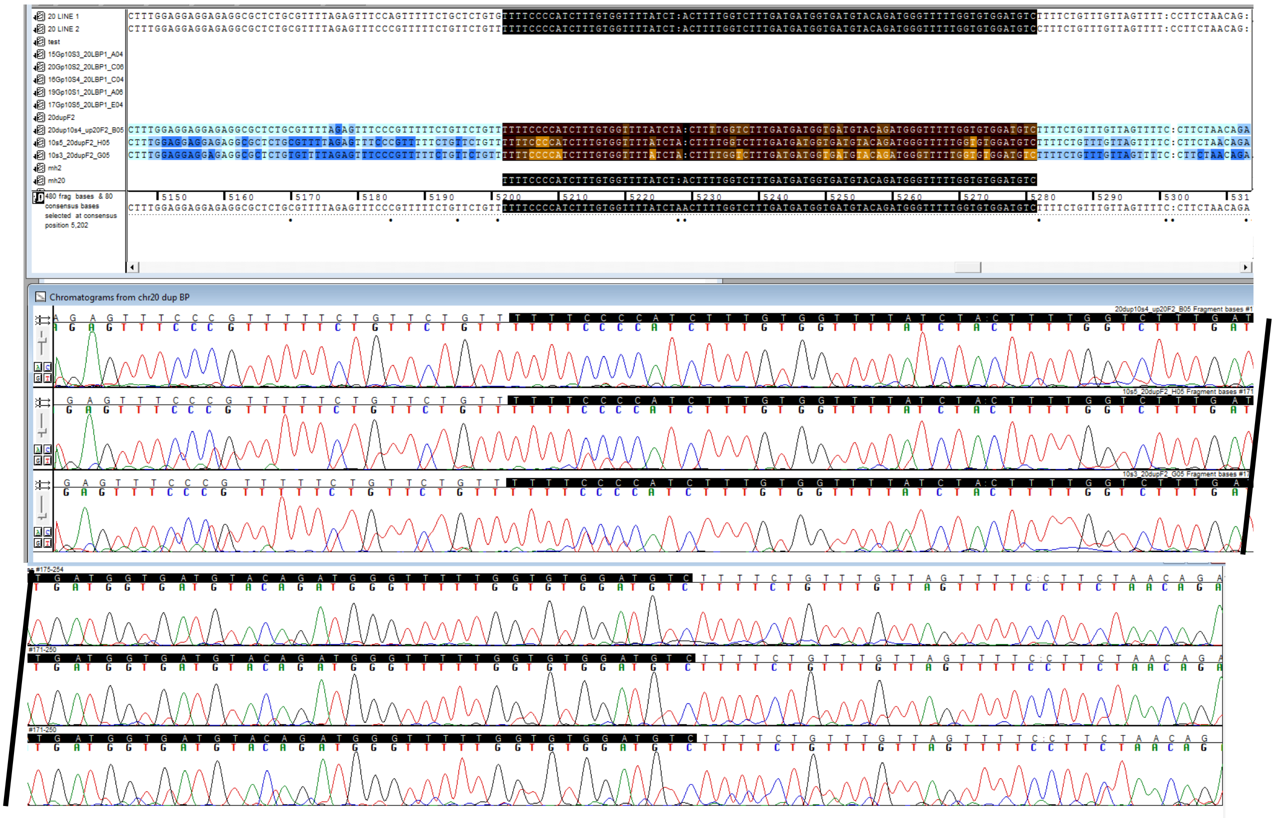

chr20 dup patient 4

Distal BP: between 8,094,645 and 8,094,725
Proximal BP: between 8,580,857 and 8,580,937
Microhomology: TTTTCCCCATCTTTGTGGTTTTATCTACTTTTGGTCTTTGATGATGG:TGATGTACAGATGGGTTTTTGGTGTGGATGTC

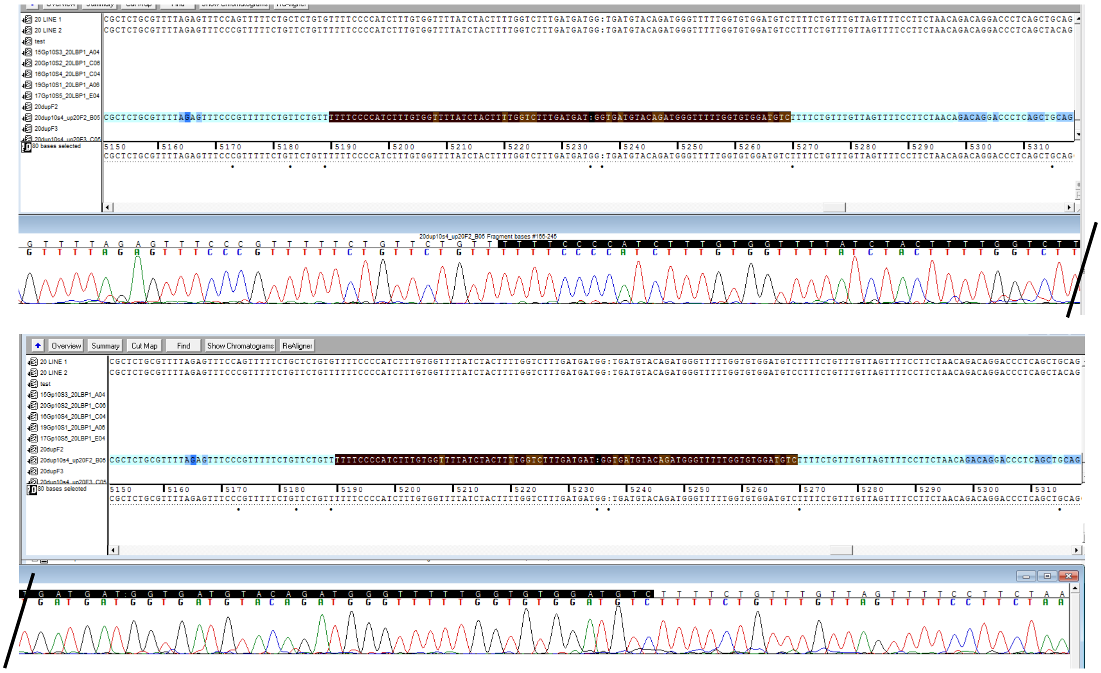

REFERENCES

1. Campbell, I. M., Gambin, T., Dittwald, P., Beck, C. R., Shuvarikov, A., Hixson, P., Patel, A., Gambin, A., Shaw, C. A., Rosenfeld, J. A. and Stankiewicz, P. (2014) Human endogenous retroviral elements promote genome instability via non-allelic homologous recombination. *BMC Biol.,* **12**, 74.
2. Campbell, I. M., Yuan, B., Robberecht, C., Pfundt, R., Szafranski, P., McEntagart, M. E., Nagamani, S. C., Erez, A., Bartnik, M., Wi?niowiecka-Kowalnik, B., Plunkett, K. S., Pursley, A. N., Kang, S. H., Bi, W., Lalani, S. R., Bacino, C. A., Vast, M., Marks, K., Patton, M., Olofsson, P., Patel, A., Veltman, J. A., Cheung, S. W., Shaw, C. A., Vissers, L. E., Vermeesch, J. R., Lupski, J. R. and Stankiewicz, P. (2014) Parental somatic mosaicism is underrecognized and influences recurrence risk of genomic disorders. *Am. J. Hum. Genet.,* **95**, 173–182.
3. Boone, P. M., Yuan, B., Campbell, I. M., Scull, J. C., Withers, M. A., Baggett, B. C., Beck, C. R., Shaw, C. J., Stankiewicz, P., Moretti, P., Goodwin, W. E., Hein, N., Fink, J. K., Seong, M. W., Seo, S. H., Park, S. S., Karbassi, I. D., Batish, S. D., Ordonez-Ugalde, A., Quintans, B., Sobrido, M. J., Stemmler, S. and Lupski, J. R. (2014) The Alu-rich genomic architecture of SPAST predisposes to diverse and functionally distinct disease-associated CNV alleles. *Am. J. Hum. Genet.,* **95**, 143–161.
4. Vissers, L. E., Bhatt, S. S., Janssen, I. M., Xia, Z., Lalani, S. R., Pfundt, R., Derwinska, K., de Vries, B. B., Gilissen, C., Hoischen, A., Nesteruk, M., Wisniowiecka-Kowalnik, B., Smyk, M., Brunner, H. G., Cheung, S. W., van Kessel, A. G., Veltman, J. A. and Stankiewicz, P. (2009) Rare pathogenic microdeletions and tandem duplications are microhomology-mediated and stimulated by local genomic architecture. *Hum. Mol. Genet.,* **18**, 3579–3593.
5. Szafranski, P., Dharmadhikari, A. V., Brosens, E., Gurha, P., Kolodziejska, K. E., Zhishuo, O., Dittwald, P., Majewski, T., Mohan, K. N., Chen, B., Person, R. E., Tibboel, D., de Klein, A., Pinner, J., Chopra, M., Malcolm, G., Peters, G., Arbuckle, S., Guiang, S. F., Hustead, V. A., Jessurun, J., Hirsch, R., Witte, D. P., Maystadt, I., Sebire, N., Fisher, R., Langston, C., Sen, P. and Stankiewicz, P. (2013) Small noncoding differentially methylated copy-number variants, including lncRNA genes, cause a lethal lung developmental disorder. *Genome Res.,* **23**, 23–33.
6. Stankiewicz, P., Sen, P., Bhatt, S. S., Storer, M., Xia, Z., Bejjani, B. A., Ou, Z., Wiszniewska, J., Driscoll, D. J., Maisenbacher, M. K., Bolivar, J., Bauer, M., Zackai, E. H., McDonald-McGinn, D., Nowaczyk, M. M., Murray, M., Hustead, V., Mascotti, K., Schultz, R., Hallam, L., McRae, D., Nicholson, A. G., Newbury, R., Durham-O'Donnell, J., Knight, G., Kini, U., Shaikh, T. H., Martin, V., Tyreman, M., Simonic, I., Willatt, L., Paterson, J., Mehta, S., Rajan, D., Fitzgerald, T., Gribble, S., Prigmore, E., Patel, A., Shaffer, L. G., Carter, N. P., Cheung, S. W., Langston, C. and Shaw-Smith, C. (2009) Genomic and genic deletions of the FOX gene cluster on 16q24.1 and inactivating mutations of FOXF1 cause alveolar capillary dysplasia and other malformations. *Am. J. Hum. Genet.,* **84**, 780–791.
7. Sanchez-Valle, A., Wang, X., Potocki, L., Xia, Z., Kang, S. H., Carlin, M. E., Michel, D., Williams, P., Cabrera-Meza, G., Brundage, E. K., Eifert, A. L., Stankiewicz, P., Cheung, S. W. and Lalani, S. R. (2010) HERV-mediated genomic rearrangement of EYA1 in an individual with branchio-oto-renal syndrome. *Am. J. Med. Genet. A,* **152A**, 2854–2860.
8. Erez, A., Patel, A. J., Wang, X., Xia, Z., Bhatt, S. S., Craigen, W., Cheung, S. W., Lewis, R. A., Fang, P., Davenport, S. L., Stankiewicz, P. and Lalani, S. R. (2009) Alu-specific microhomology-mediated deletions in CDKL5 in females with early-onset seizure disorder. *Neurogenetics,* **10**, 363–369.
9. Needleman, S. B. and Wunsch, C. D. (1970) A general method applicable to the search for similarities in the amino acid sequence of two proteins. *J. Mol. Biol.,* **48**, 443–453.
10. Eddy, S. R. (2004) What is a hidden Markov model? *Nat. Biotech.,* **22**, 1315–1316.
11. Welch, L. R. (2003) Hidden Markov Models and the Baum-Welch Algorithm. *IEEE Information Theory Society Newsletter,* **53**, 1.
12. Lawrence R. Rabiner, B. H. J. (1986) An introduction to hidden Markov models. *IEEE ASSP Magazine,* p. 1.
13. Ewing, B., Hillier, L., Wendl, M. C. and Green, P. (1998) Base-calling of automated sequencer traces using phred. I. Accuracy assessment. *Genome Res.,* **8**, 175–185.
14. Ewing, B. and Green, P. (1998) Base-calling of automated sequencer traces using phred. II. Error probabilities. *Genome Res.,* **8**, 186–194.
